# Supplementary material for: Flash glucose monitoring with the FreeStyle Libre 2 compared with self-monitoring of blood glucose in suboptimally controlled type 1 diabetes: the FLASH-UK randomised controlled trial protocol
Source: BMJ Open. 2021 Jul 14;11(7):e050713. doi: 10.1136/bmjopen-2021-050713 (PMC8280849; doi:10.1136/bmjopen-2021-050713)
Supplement: Supplementary data [file bmjopen-2021-050713supp001.pdf]

Supplementary File

Flash-glucose monitoring with the FreeStyle Libre 2 compared to self-monitoring of blood glucose in sub-optimally controlled type 1 diabetes: the FLASH-UK randomised controlled trial protocol

Contents

1. Model Participant Information Sheet (Page 2)
2. Model Consent form (Page 18)
3. Data Sharing Plan (Page 20)

Wilmot EG, *et al. BMJ Open* 2021; 11:e050713. doi: 10.1136/bmjopen-2021-050713

1. Model Participant Information Sheet

Flash-glucose monitoring in sub-optimally controlled Type 1 diabetes (FLASH-UK)

IRAS ID: 257593

Chief Investigator

Dr Lala Leelarathna

Lalantha.Leelarathna@mft.nhs.uk

Principal Investigators for local site

Flash-glucose monitoring in sub-optimally controlled Type 1 diabetes (FLASH-UK)

We would like to invite you to take part in a research study. Before you decide, it is important for you to understand why the research is being done and what it will involve. Please take time to read the following information carefully and discuss it with others if you wish.

- **Part 1 tells you the purpose of this study and what will happen to you if you take part.**
- **Part 2 gives you more detailed information about the conduct of the study.**

Ask us if there is anything that is not clear or if you would like more information. Take time to decide whether or not you wish to take part.

Part 1

Introduction

In type 1 diabetes controlling blood glucose levels is very important for good health. A number of research studies have shown that having high glucose levels in diabetes can lead to long-term problems. In order to lower the risk of these complications, maintaining glucose as close to normal range as possible is recommended. However, the achievement of near-normal glucose levels can be extremely difficult.

Currently the dose of prescribed insulin depends on the results of finger stick testing. The more frequent the testing, the easier it is to adjust the insulin dose. However many people struggle to perform frequent blood glucose tests due to pain and inconvenience.

Wilmot EG, *et al. BMJ Open* 2021; 11:e050713. doi: 10.1136/bmjopen-2021-050713

The FreeStyle Libre 2 flash glucose monitor is a device which makes monitoring of glucose levels easier. It continuously monitors the glucose levels and you are able to see the level and trend by scanning the disc, worn on your arm, with a hand held device.

What is the purpose of the study?

The FreeStyle Libre device has been shown as a safe replacement for blood glucose monitoring in people with well controlled diabetes. However, there are no randomised controlled studies in adults with type 1 diabetes demonstrating improvements in HbA1c levels.

The purpose of this study is to assess the benefit of using FreeStyle Libre 2 flash glucose monitoring system, in terms of improving glucose levels as measured by HbA1c. The study will also assess the acceptability and the impact of the system on daily living with diabetes.

Why have I been invited?

You have been invited because you have Type 1 diabetes and have an HbA1c level between 58mmol/mol (7.5%) and 97mmol/mol (11%) and use either multiple daily insulin injections or use an insulin pump for controlling your blood glucose. Up to 180 participants aged 16 and above with type 1 diabetes will be involved in this project.

If you agree to take part in the study then you will be randomly allocated to either continue usual finger-stick blood glucose monitoring or Flash glucose monitoring with the FreeStyle Libre 2 device for 6 months. During this time you will either attend 5-7 research clinic visits or participate in 5-7 virtual consultations using video calls (or a combination of the two with telephone calls allowed for some visits) for data collection, device training and support to manage your diabetes. You can decide whether to attend clinic visits or virtual assessments but this will also depend on whether your hospital is operating normal clinics because of the COVID-19 pandemic.

Do I have to take part in this research?

No. It is up to you to decide whether or not to take part. If you do, you will be given more detailed information and will be asked to sign a consent form. You are still free to withdraw at any time and

without giving any reason. A decision to withdraw at any time, or a decision not to take part, will not affect the standard of care you receive.

What will happen to me if I take part?

The study consists of 5-7 visits with the diabetes research team (either in person or by virtual consultation) over a 6 month period. During these visits you will be supported to improve your diabetes control. Blood tests are done three times during this 6 month period. If you attend virtual consultations the information sheet / consent form (for visit 1), necessary questionnaires or blood collection kit will be provided by post before these take place and they can be returned by pre-paid postal delivery.

The following sections describe the study visits in detail.

**Visit 1 - Recruitment visit**	**(up to 2 hours)**

Once you have had sufficient time to read this information sheet, if you would like to hear more about the study or would like to participate, you will be invited to attend the recruitment visit (either in person or by virtual consultation). You will be provided with detailed information about the study and any questions you have will be answered. If you decide to participate, you will be asked to sign a consent form. By signing the form you are telling us that you understand what you have read and discussed, consent to take part in the research project and consent to the use of your information as described.

If would like to consider joining the trial by virtual consultation, you will receive the consent form along with this information sheet by post before the recruitment visit is scheduled. Any questions you have can be answered by email, telephone or video consultation. If you decide to participate, you will need to provide initials to each declaration box on the consent form, sign it and return it to the research team in a pre-paid envelope. Once the research team receive your fully completed consent form, they will schedule your virtual recruitment consultation. At the start of the virtual recruitment consultation, the investigator will ask if you are happy to proceed and they will sign / date the consent form. A copy of the fully completed consent form will be returned to you. Once you receive this, you can let the research team know before or at your next visit.

At recruitment visit, if you consent to take part the following data will be captured:

History: medical and diabetes history, current medications and insulin therapy recorded.

Measurements: Height, weight (if possible).

Blood test : HbA1c, (less than 10 mls). If conducted as a virtual visit, we will send you a pre-paid pack to collect blood into a small tube from a finger-stick. This can be returned by post in the pre-paid envelop provided.

Questionnaires: to assess a range of measures which include diabetes treatment satisfaction, diabetes distress, quality of life, fear of injections/testing, eating behaviours, hypoglycaemia. If conducted as a virtual consultation, we will send you a pre-paid envelope to return any questionnaires that cannot be completed by video call.

Personal Data: Initials, Date of Birth, Gender & Full Postcode.

If you intend to become pregnant within the next six months then you should notify the research team. If you are pregnant at the recruitment visit, or should you become pregnant during the study, you will not be able to take any further part.

As with many research studies, in order to proceed with the study certain recruitment criteria need to be met. In the event you don't meet the recruitment criteria your participation in the study will end after visit 1.

Visit 2 (Or combined with visit 1) – Blinded flash glucose monitor insertion (30 minutes)

Within 1 to 2 weeks of visit 1 (or at the same time as visit 1 if easier and recruitment criteria are met), a glucose sensor will be inserted to collect information about glucose data. The sensor looks the same as the Freestyle Libre sensor and is placed in the same area. The purpose of this is to compare results at the end with the results at the beginning. In order to avoid any change in behaviour or insulin doses, glucose sensor data will not be visible to you (Blinded sensor) during these first 2 weeks. It is important that you do not try to obtain the blinded glucose data as this will affect the comparison of before and after data. You are asked to wear the sensor for 14 days. If this visit is conducted as a virtual consultation we will send you the sensor and reader for self-insertion and provide guidance on how to apply the sensor. After 14 days you will need to post the sensor and reader back to us in a pre-paid envelope provided.

Visit 3 – Randomisation, start of study treatment & education (up to 2 hours)

During Visit 3, we will check that you were comfortable wearing the flash glucose monitor for the 14 days. (To proceed with the study the blinded glucose sensor should have been worn for a minimum of 10 days'). If you are happy to proceed you will be randomly assigned to either flash glucose monitoring with the FreeStyle Libre 2 device for 6 months or continuing with finger-stick glucose tests for 6 months.

Visit 3:

Randomisation: to either flash glucose monitoring or finger-stick glucose tests for the next 6 months.

Education and support: If you are allocated to flash glucose monitoring, the research team will spend some time demonstrating how to insert the device, what the data means and how you safely use the device. This will include a discussion of how to use the device to review data and make adjustments to your insulin therapy. You will be shown how to download and review data at home. If you are continuing with finger-stick blood tests the study team will review your diabetes therapy and support you to make improvements. For those attending virtual consultations and who are assigned to the flash glucose monitoring arm, the research team will send you all the devices you need and easy to follow instructions and links to self-help videos. The Research team will conduct additional training through video consultation as required.

Given participant diary: to record information about insulin doses and carbohydrate intake in last 5 days before study visits 4, 5 and 7. If you are attending virtual consultations, the research team will send diaries for you to complete and these can be returned by pre-paid postal delivery or scanned and returned by email.

Downloading of devices: data from your glucose meter (and insulin pump if you are on one) will be downloaded where possible.

Visit 4 – Data review and education support (up to 1 hour)

Four weeks after visit 3 you will be asked to come to the clinic or attend a virtual clinic consultation, with your glucose meter (flash glucose reader or blood glucose meter) and the completed diary which captures your insulin doses and carbohydrate intake. The research team will work with you to review your insulin therapy, supporting you to adjust your insulin to improve your diabetes control. Data from your glucose meters (and insulin pump if you are on one) will be downloaded to secure web-based programmes similar to what is used in clinical practice.

Visit 5 - Data review and education support (up to 2 hours)

Eight weeks after visit 4 you will be seen in clinic or by virtual clinic consultation. The purpose of this visit is to provide ongoing support and training to help you improve your diabetes control. You are required to bring your glucose meters and diary with insulin doses. A HbA1c blood test will also be performed at this visit (blood sample collected at home and returned by pre-paid envelope for those attending virtual consultations). Data from your glucose meters (and insulin pump if you are on one) will be downloaded to secure web-based programmes similar to what is used in clinical practice.

Visit 6 – Finger-stick glucose testing group only: blinded sensor (<30 minutes)

If you are allocated to the finger-stick glucose testing group you will be asked to return to clinic or attend a virtual consultation 10 weeks after visit 5 to have a blinded sensor attached. If conducted as a virtual consultation, we will send you the devices you need and easy to follow instructions and self-help videos (like visit 1). The blinded FreeStyle Libre sensor will be worn for 2 weeks. This will allow the collection of glucose data to see how your diabetes control has changed compared to your glucose control when you entered the study. After 14 days you will need to post the sensor and reader back to us by pre-paid envelope if you attended a virtual consultation.

Visit 7 - End of study visit (upto 2 hours)

Approximately 12 weeks after Visit 5 (or 2 weeks after visit 6 for the finger-stick glucose testing group), you will be asked to attend the clinic or virtual consultation. This will be the final visit. Data from your glucose meters (and insulin pump if you are on one) will be downloaded to secure web-based programmes similar to what is used in clinical practice where possible. We will also collect paper diaries.

During visit 7 the following occurs:

Blood test: HbA1c (blood sample collected at home for virtual consultations).

Questionnaires: same as those completed at the start of the study. In addition if you were randomised to Flash glucose monitoring arm, you will be invited to complete two additional questionnaires to capture your experience and expectations during the study (If you attend a virtual consultation any questionnaires that cannot be completed will be provided for return in a pre-paid envelope).

Data download and review: to support you to improve glucose control.

Weight measured: where possible.

Communication with research team for advice and support by e-mail

If you choose to communicate with us by e-mail, we will use a dedicated e-mail account as a way to communicate with you. We will use reasonable means to protect the security and confidentiality of e-mail information sent and received. The research team will take every care to remove any identifying material from the responses you provide as early as possible. Likewise, individuals' responses will be kept confidential by the researcher and participants will not be identified in the reporting of the research. However, the researcher cannot guarantee the confidentiality or anonymity of material transferred by email or the internet. There are known and unknown risks that may affect privacy when using e-mail to communicate. These risks include, but are not limited, to:

- E-mail can be forwarded, printed, and stored in numerous paper and electronic forms and be received by many intended and unintended recipients without my knowledge or agreement.
- E-mail may be sent to the wrong address by any sender or receiver.
- E-mail is easier to forge than handwritten or signed papers.
- Copies of e-mail may exist even after the sender or the receiver has deleted his or her copy.
- E-mail service providers have a right to keep and inspect emails sent through their systems.
- E-mail may be intercepted and read during transmission without detection or authorisation.
- E-mail is not a secure way of corresponding and it is advisable not to transfer any sensitive information in this format.
- E-mail can spread computer viruses.

Choosing to participate in the trial by virtual consultation

If you choose to participate in the trial by use of audio / video communication, we will use a dedicated videoconferencing account as a way to communicate with you. We will use reasonable means to protect the security of the connection and virtual consultations will not be recorded. However, the researcher cannot guarantee the confidentiality or anonymity of material transferred over the internet and stored within devices. There is an increased security risk that your health information may be intercepted or disclosed to third parties when using video or audio communications tools. To help us keep your information safe and secure, you can:

- Understand that this method of communication is not secure in the same way as a private appointment in an exam room.
- Use a private computer / device (i.e., not an employer's or third party's computer / device) and a secure internet connection. For example, using a personal computer or tablet is more secure than at a library, and your access to the Internet on your home network will generally be more secure than an open guest Wi-Fi connections.

By providing your information, you agree to let us collect, use, or disclose your personal health information through video or audio communications (while following applicable privacy laws) in order to provide you with care. In particular, the following means of electronic communication may be used for videoconferencing, including Skype, Microsoft Teams and Zoom.

Will I be reimbursed?

There is no payment for taking part in the study. If you are randomised to the flash glucose monitoring arm then the sensors will be provided to you for 6 months, free of charge. The blinded sensors that will be provided at Visit 1 or 2 and at visit 6 (those assigned to finger-stick glucose testing) will also be provided free of charge. Reasonable travelling expenses will be reimbursed if you attend any visits in person at the study clinics.

What are the possible risks of taking part?

During this study, we will support you to improve your glucose control. Sometimes when improving glucose control you may experience a 'hypo', similar to the ones that happen normally in everyday life. We will ask you to treat it in the same way you normally treat a hypo. There is a risk of possible mild to moderate hyperglycaemia (high glucose) which is similar to the risk that a person with type 1 diabetes experiences on a daily basis. There is also a risk of hyperglycaemia leading to diabetic ketoacidosis (for example if the pump is blocked for a significant time or if you miss insulin doses or

become unwell); however this risk is low and similar to the risk that a person with type 1 diabetes experiences.

You will have to attach a small glucose sensor on the upper arm. The sensor needs to be changed every 14 days. Inserting the sensor has a low risk of developing a local skin reaction or infection. Some bruising, itchiness, redness and bleeding at the site of sensor insertion may occur.

In addition, when the blood samples are taken for the study, some bruising or minor discomfort may also occur at the site where the blood was taken.

What are the possible benefits of taking part?

You will have regular contact with the study team who will support you to improve your glucose control and this may have benefits beyond the study period. Results of the study may pave the way for better access for flash glucose monitoring for people living with type 1 diabetes.

Who should I talk to if I have any questions or concerns?

If you have any questions regarding this study, please contact one of the following people:

Contact details of the local clinical study team

On behalf of the Study Team, we would like to thank you for taking the time to consider participating in this important research programme.

This completes part 1 of the Information Sheet.

Wilmot EG, *et al. BMJ Open* 2021; 11:e050713. doi: 10.1136/bmjopen-2021-050713

If the information in Part 1 has interested you and you are considering participation, please continue to read the additional information in Part 2 before making any decision.

Part 2

What if relevant new information becomes available?

You will be informed as soon as possible if any new information becomes available during the course of the study that may affect your willingness to participate in the study. If you decide not to continue, the study doctor will arrange for your care to continue.

What will happen if I don't want to carry on with the study?

If you decide not to participate in this study, it will not affect your future management in any way. If you agree to take part, please remember that you are free to withdraw at any time without explanation. All you need to do is tell us. If you withdraw from the study, we will destroy all your identifiable samples, but we will need to use the data collected up to your withdrawal.

What if there is a problem?

If you have a concern about any aspect of this study, please ask to speak to the researchers who will do their best to answer your questions (see contact details above). If you are unhappy about the conduct of the study and wish to complain, you can do this through (name and contact details of the local patient advice and liaison service – institution specific).

In the unlikely event that something does go wrong and you are harmed during the research study, appropriate healthcare arrangements will be made. Healthcare arrangements may include advice from clinical members of the study team or your local diabetes clinic team, or use of emergency health services. There are no special compensation arrangements unless this was due to the negligence of one of the doctors or nurses. In this case you may have grounds for legal action for compensation but you may have to pay your legal costs. The normal hospital complaints mechanism will still be available to you.

Under what circumstances might the study be stopped?

The study may be stopped, or you will be asked to withdraw from the study, under the following circumstances:

- Serious harm to any participant in the study
- You have significant difficulties using the study devices during the training period(s)
- You develop significant allergy to the plaster holding the sensor
- Failure to follow the instructions of the study team and misuse of the study devices
- Decision by the study team, or the sponsor, that stopping the study is in your best medical interest
- Pregnancy, planned pregnancy, or breast feeding
- Technical reasons (e.g. moving home outside the region)

Should the study be stopped for any reason, then you will be informed as soon as possible and your doctor will arrange for your care to continue.

Will my GP be informed?

We will inform your GP of your participation in this study after having gained your consent for this.

Will my taking part in the study be kept confidential?

Strict confidentiality will be maintained at all times. All collected written data will be kept in a locked cupboard, accessible only by the research team.

Only the researchers who are directly involved in the study, the study sponsor (Manchester University NHS Foundation Trust), the study monitor and regulatory authority who will be involved in monitoring and auditing of the project will have access to the identifiable data, as permitted by applicable laws and regulations. By signing the written informed consent form, you are authorising such access. Anonymised data collected during the study may also be sent to associated researchers in EU and USA where the laws do not protect your privacy to the same extent as the law in the UK. We will however take all reasonable steps to protect your privacy. Data will be stored for 15 years and will be disposed of securely thereafter.

Compliance with General Data Protection (GDPR) Rules

Manchester University NHS Foundation Trust is the sponsor for this study based in the United Kingdom. We will be using information from you and/or your medical records in order to undertake this study and will act as the data controller for this study. This means that we are responsible for looking after your information and using it properly. Manchester University NHS Foundation Trust will keep identifiable information about you [for three years after the study has finished].

Your rights to access, change or move your information are limited, as we need to manage your information in specific ways in order for the research to be reliable and accurate. If you withdraw from the study, we will keep the information about you that we have already obtained. To safeguard your rights, we will use the minimum personally-identifiable information possible.

You can find out more about how we use your information at https://research.cmft.nhs.uk/getting-involved/gdpr-and-research and by contacting our Data Protection Officer at dpo@mft.nhs.uk.

[Name of NHS hospital] will collect information from you and/or your medical records for this research study in accordance with our instructions.

[Name of NHS hospital] will keep your name, [NHS number] and contact details [add other identifiers] confidential and will not pass this information to Manchester University NHS Foundation Trust. [Name of NHS hospital] will use this information as needed, to contact you about the research study, and make sure that relevant information about the study is recorded for your care, and to oversee the quality of the study. Certain individuals from Manchester University NHS Foundation trust, Manchester Clinical Trials Unit and regulatory organisations may look at your medical and research records to check the accuracy of the research study. Manchester University NHS Foundation Trust will only receive information without any identifying information. The people who analyse the information

will not be able to identify you and will not be able to find out your name, [NHS number] or contact details.

[Name of NHS hospital] will keep identifiable information about you from this study for three years after the study has finished.

What will happen to any samples I give?

As above, blood samples collected during this study will be labelled and sent to the lab for processing, in line with standard accredited practice. Once the measurements have been completed the samples will be disposed of securely.

What will happen to the data collected?

After each visit fully anonymised data will be entered onto a computer and will be used to analyse the results once the study is finished. These study results may be presented at scientific meetings or published in a scientific journal. In addition, fully anonymised data may be shared with researchers and collaborating partners.

Your personal details will be kept for up to 3 years after the end of the study so that we can inform you the results of the study.

Who is organising and funding the research?

The study will be coordinated from the Manchester Clinical Trials Unit, University of Manchester . The research study has been funded by Diabetes UK charity. The sponsor of the study is the Manchester University NHS Foundation Trust.

Who has reviewed the study?

Before any research goes ahead it has to be checked by an Ethics Committee. This project has been reviewed by the Greater Manchester West Research Ethics Committee.

Wilmot EG, *et al. BMJ Open* 2021; 11:e050713. doi: 10.1136/bmjopen-2021-050713

If I agree to join the study

The study will be explained to you in more detail during the recruitment visit. During this visit you will be able to ask questions and voice any queries. Once you have agreed to take part we will ask you to sign a consent form. The study team will then arrange the dates for your training and hospital visits.

What will happen at the end of the study?

At the end of this study you will go back to using your usual glucose monitoring device. Your diabetes care will then be looked after by your own diabetes team.

This completes part 2 of the Information Sheet. Thank you for your time and interest in our research.

(site/institution address)

Wilmot EG, *et al. BMJ Open* 2021; 11:e050713. doi: 10.1136/bmjopen-2021-050713

2. Consent form

PARTICIPANT CONSENT FORM

Title of Project: Flash-glucose monitoring in sub-optimally controlled type 1 diabetes (FLASH-UK), IRAS ID 257593

	**Please Provide Initials in Each Box**
Name of Principal Investigator: Dr ………………………………………………	
I confirm that I have read and understood the information sheet version .... dated .... for the above study and have had the opportunity to consider the information and I am satisfied with the answers I have been given.	☐
I understand that my participation is voluntary and that I am free to withdraw at any time, without giving any reason, without my medical care or legal rights being affected in any way.	☐
I give consent for the taking of blood samples.	☐
If I want to change my mind in the future and withdraw my consent for these studies, then I understand that, if I request it, the identifiable data collected will be destroyed. Unidentifiable data would be retained.	☐
I give consent to my GP being informed of my participation in this study.	☐
I give consent to the sharing of anonymised research data arising from this study with other study partners in Europe and USA.	☐
If I choose to communicate with the research team via email / virtual consultation, I acknowledge the associated risks as outlined in the information sheet.	☐

I understand that relevant sections of my medical notes and data collected during the study may be looked at by individuals from the NHS Trust, the sponsor (or their delegate) and regulatory bodies for audit or monitoring purposes. ☐

I agree to return the study devices at the end of the study or earlier if consent is withdrawn. ☐

I agree to follow instructions from the study team and take part in the study. ☐

____________________________	__________________	_______________________
Name of patient	Date	Signature

___________________________	_________________	_____________________
Name of Researcher taking consent	Date	Signature

Copies: *1 for patient; 1 for researcher; 1 to be kept with hospital notes*

3. Data Sharing plan

Data Sharing Plan – FLASH-UK study	
Individual participant data availability (anonymised)	Yes
What data will be shared	Individual participant data that underlie the results reported in the primary study manuscript after deidentification (text, tables, figures and appendices)
What other documents will be available	The study protocol, Statistical analysis plan, Informed consent form, data dictionary and statistical code.
Data availability	Beginning 6 months and ending 3 years following article publication
With whom	Researchers who provide a methodologically sound proposal not overlapping with any planned secondary publications from the research team.
For what type of analyses	To achieve aims in the approved proposal
By what mechanism will data be available	Proposals should be directed to Chief Investigator who will discuss such requested with the Trial Management Group (TMG). Lalantha.leelarathna@mft.nhs.uk.<br><br>To gain access, data requesters will need to sign a data access agreement. Data will be available for 3 years.
