# Supplementary material for: Flash glucose monitoring with the FreeStyle Libre 2 compared with self-monitoring of blood glucose in suboptimally controlled type 1 diabetes: the FLASH-UK randomised controlled trial protocol
Source: BMJ Open. 2021 Jul 14;11(7):e050713. doi: 10.1136/bmjopen-2021-050713 (PMC8280849; doi:10.1136/bmjopen-2021-050713)
Supplement: Supplementary data [file bmjopen-2021-050713supp002.pdf]

Appendix 1: Data sharing plan. Clinicaltrials.gov record also updated.

Data Sharing Plan – FLASH-UK study	
Individual participant data availability (anonymised)	Yes
What data will be shared	Individual participant data that underlie the results reported in the primary study manuscript after deidentification (text, tables, figures and appendices)
What other documents will be available	The study protocol, Statistical analysis plan, informed consent form, data dictionary and statistical code.
Data availability	Beginning 6 months and ending 3 years following article publication
With whom	Researchers who provide a methodologically sound proposal not overlapping with any planned secondary publications from the research team.
For what type of analyses	To achieve aims in the approved proposal
By what mechanism will data be available	Proposals should be directed to Chief Investigator who will discuss such requested with the Trial Management Group (TMG). Lalantha.leelarathna@mft.nhs.uk.<br><br>To gain access, data requesters will need to sign a data access agreement. Data will be available for 3 years at University Of Manchester website.

Wilmot EG, *et al. BMJ Open* 2021; 11:e050713. doi: 10.1136/bmjopen-2021-050713
