# Supplementary material for: Flash glucose monitoring with the FreeStyle Libre 2 compared with self-monitoring of blood glucose in suboptimally controlled type 1 diabetes: the FLASH-UK randomised controlled trial protocol
Source: BMJ Open. 2021 Jul 14;11(7):e050713. doi: 10.1136/bmjopen-2021-050713 (PMC8280849; doi:10.1136/bmjopen-2021-050713)
Supplement: Author's manuscript [file bmjopen-2021-050713.draft_revisions.pdf]

BMJ Open

BMJ Open is committed to open peer review. As part of this commitment we make the peer review history of every article we publish publicly available.

When an article is published we post the peer reviewers' comments and the authors' responses online. We also post the versions of the paper that were used during peer review. These are the versions that the peer review comments apply to.

The versions of the paper that follow are the versions that were submitted during the peer review process. They are not the versions of record or the final published versions. They should not be cited or distributed as the published version of this manuscript.

BMJ Open is an open access journal and the full, final, typeset and author-corrected version of record of the manuscript is available on our site with no access controls, subscription charges or pay-per-view fees (http://bmjopen.bmj.com).

If you have any questions on BMJ Open's open peer review process please email info.bmjopen@bmj.com

Flash-glucose monitoring with the FreeStyle Libre 2 compared to self-monitoring of blood glucose in sub-optimally controlled type 1 diabetes: the FLASH-UK randomised controlled trial protocol

Journal:	*BMJ Open*
Manuscript ID	bmjopen-2021-050713
Article Type:	Protocol
Date Submitted by the Author:	01-Mar-2021
Complete List of Authors:	Wilmot, Emma; University Hospitals of Derby and Burton NHS Foundation Trust; University of Nottingham Faculty of Medicine and Health Sciences<br>Evans, Mark; Cambridge University, Institute of Metabolic Science<br>Barnard-Kelly, Katharine; Bournemouth University<br>Burns, M; The University of Manchester<br>Cranston, Iain; Portsmouth Hospitals NHS Trust<br>Elliott, Rachel; Manchester Centre for Health Economics<br>Gkountouras, G; The University of Manchester<br>Kanumilli, N<br>Krishan, A; The University of Manchester<br>Kotonya, C; University Hospitals of Derby and Burton NHS Foundation Trust<br>Lumley, S<br>Narendran, P; University of Birmingham College of Medical and Dental Sciences<br>Neupane, Sankalpa; Norfolk and Norwich University Hospital NHS Trust, Elsie Bertram Diabetes Centre<br>Rayman, Gerry; Ipswich Hospital NHS Trust, The Ipswich Diabetes Centre and Research Unit<br>Sutton, Christopher; University of Manchester, Division of Population Health, Health Services Research & Primary Care<br>Taxiarchi, V P; The University of Manchester<br>Thabit, H; Manchester University NHS Foundation Trust; The University of Manchester<br>Leelarathna, L; Manchester University NHS Foundation Trust; The University of Manchester
Keywords:	General diabetes < DIABETES & ENDOCRINOLOGY, HEALTH ECONOMICS, Clinical trials < THERAPEUTICS, Diabetes & endocrinology < INTERNAL MEDICINE

For peer review only - http://bmjopen.bmj.com/site/about/guidelines.xhtml

For peer review only - http://bmjopen.bmj.com/site/about/guidelines.xhtml

I, the Submitting Author has the right to grant and does grant on behalf of all authors of the Work (as defined in the below author licence), an exclusive licence and/or a non-exclusive licence for contributions from authors who are: i) UK Crown employees; ii) where BMJ has agreed a CC-BY licence shall apply, and/or iii) in accordance with the terms applicable for US Federal Government officers or employees acting as part of their official duties; on a worldwide, perpetual, irrevocable, royalty-free basis to BMJ Publishing Group Ltd ("BMJ") its licensees and where the relevant Journal is co-owned by BMJ to the co-owners of the Journal, to publish the Work in this journal and any other BMJ products and to exploit all rights, as set out in our licence.

The Submitting Author accepts and understands that any supply made under these terms is made by BMJ to the Submitting Author unless you are acting as an employee on behalf of your employer or a postgraduate student of an affiliated institution which is paying any applicable article publishing charge ("APC") for Open Access articles. Where the Submitting Author wishes to make the Work available on an Open Access basis (and intends to pay the relevant APC), the terms of reuse of such Open Access shall be governed by a Creative Commons licence – details of these licences and which Creative Commons licence will apply to this Work are set out in our licence referred to above.

Other than as permitted in any relevant BMJ Author's Self Archiving Policies, I confirm this Work has not been accepted for publication elsewhere, is not being considered for publication elsewhere and does not duplicate material already published. I confirm all authors consent to publication of this Work and authorise the granting of this licence.

For peer review only

Flash-glucose monitoring with the FreeStyle Libre 2 compared to self-monitoring of blood glucose in sub-optimally controlled type 1 diabetes: the FLASH-UK randomised controlled trial protocol

Wilmot E G[1], Evans M L[2], Barnard-Kelly K[3], Burns M[4], Cranston I[5], Elliott R A[6] , Gkountouras G[6], Kanumilli N[7] Krishan A[6], Kotonya C[8], Lumley S[9] , Narendran P[10], Neupane S[11], Rayman G[12], Sutton C J[6], Taxiarchi V P[6], Thabit H[13, 14] , Leelarathna L[13, 14].

[1]University Hospitals of Derby and Burton NHS Foundation Trust, Royal Derby Hospital, Derby, UK.
[2]Wellcome Trust-MRC Institute of Metabolic Science, NIHR Cambridge Biomedical Research Centre, Cambridge University Hospitals and University of Cambridge, Cambridge, UK
[3]Barnard Health, BHR Limited, Hampshire, UK
[4]Manchester Clinical Trials Unit, University of Manchester, Manchester, UK
[5]Academic Department of Diabetes & Endocrinology, Queen Alexandra Hospital, Cosham, Portsmouth, UK
[6]Manchester Centre for Health Economics, Division of Population Health, Health Service Research & Primary Care, University of Manchester, Manchester, UK
[7]Northenden Group Practice, 489 Palatine Road, Northernden, Manchester, UK
[8]University Hospitals of Derby and Burton NHS Foundation Trust, Queen's Hospital Burton, Burton-on-Trent, UK
[9]The Adam Practice, Upton, Poole, Dorset, UK
[10]Institute of Immunology and Immunotherapy, College of Medical and Dental Sciences, University of Birmingham, Edgbaston, Birmingham, UK
[11] Elsie Bertram Diabtes Centre ,Norfolk and Norwich University Hospitals NHS Foundation Trust, Colney Lane, Norwich, UK
[12]Diabetes Research Office, East Suffolk and North Essex NHS Foundation Trust, Ipswich, UK
[13]Manchester Diabetes Centre, Manchester Royal Infirmary, Manchester University NHS Foundation Trust, Manchester Academic Health Science Centre, Manchester, UK
[14]Division of Diabetes, Endocrinology and Gastroentrology, Faculty of Biology, Medicine and Health, University of Manchester, Manchester, UK

Corresponding authors:

Dr Lalantha Leelarathna

Email: Lalantha.Leelarathna@mft.nhs.uk

And

Dr Emma Wilmot

Email: emma.g.wilmot@gmail.com

Word count: 3990 excluding Abstract, Box and Tables (max 4000)
Abstract: 300
Figures: 1, Boxes: 1, Tables 2

For peer review only - http://bmjopen.bmj.com/site/about/guidelines.xhtml

Abstract (max 300 words)

Introduction Optimising glycaemic control in Type 1 diabetes (T1D) remains challenging. Flash glucose monitoring with FreeStyle Libre 2 (FSL2) is a novel alternative to the current standard of care self-monitoring of blood glucose (SMBG). No randomised controlled trials to date have explored the potential benefits of FSL2 in T1D. We aim to assess the impact of FSL2 in people with sub-optimal glycaemic control T1D in comparison with SMBG.

Methods This open-label, multi-centre, randomised (via stochastic minimisation), parallel design study conducted at 8 UK secondary and primary care centres will aim to recruit 180 people age ≥16 years with T1D for >1 year and HbA1c 7.5%-11%. Eligible participants will be randomised to 24 weeks of FSL2 (intervention) or SMBG (control) periods, after two week of blinded sensor wear. Participants will be assessed virtually or in-person owing to the COVID-19 pandemic. HbA1c will be measured at baseline, 12 and 24 weeks (primary outcome). Participants will be contacted at 4 and 12 weeks for glucose optimisation. Control participants will wear a blinded sensor during the last 2 weeks. Psychosocial outcomes will be measured at baseline and 24 weeks. Secondary outcomes include sensor-based metrics, insulin doses, adverse events and self-report psychosocial measures. Utility, acceptability, expectations and experience of using FSL2 will be explored. Data on health service resource utilisation will be collected.

Analysis Efficacy analyses will follow intention-to-treat principle. Outcomes will be analysed using Analysis of Covariance, adjusted for the baseline value of the corresponding outcome, minimisation factors and other known prognostic factors. Both within-trial and life-time economic evaluations, informed by modelling from the perspective of the NHS setting, will be performed.

Ethics and trial registration The study was approved by Greater Manchester West Research Ethics Committee (reference 19/NW/0081). Informed consent will be sought from all participants. The study is registered at ClinicalTrials.gov (NCT03815006).

For peer review only

For peer review only - http://bmjopen.bmj.com/site/about/guidelines.xhtml

Trial registration number: ClinicalTrials.gov NCT03815006 (23/01/2019)

The study website: https://sites.manchester.ac.uk/flash/

Protocol version: 4.0 dated 29/06/2020

Trial Sponsor:

Manchester University NHS Foundation Trust
Division of Research & Innovation
Manchester Royal Infirmary,
Oxford Road
Manchester
M13 9WL

For peer review only

For peer review only - http://bmjopen.bmj.com/site/about/guidelines.xhtml

Strengths and limitations of this study

- Flash-UK is a multi-centre randomised controlled trial of the novel FreeStyle Libre 2 flash glucose monitor over a 6 month follow up period assessing the impact on people living with type 1 diabetes and sub-optimal glucose control, in comparison to self-monitoring of blood glucose, with a primary outcome of change in HbA1c.

- It is the first randomised study to assess the clinical efficacy & health economic benefits of the Libre 2 device providing high quality data for UK policy makers.

- The integration of a virtual assessment pathway into the trial design ensures eligible candidates are not restricted from participation owing to the COVID-19 pandemic.

- A wide range of secondary outcomes including continuous glucose monitoring data and psychosocial outcomes will provide detailed insight into the impact of this technology on people living with Type 1 diabetes.

- The study is open (unblinded) and conducted in UK National Health Service only. Some findings from the study may only be applicable to UK NHS setting.

For peer review only - http://bmjopen.bmj.com/site/about/guidelines.xhtml

Introduction

Type 1 diabetes mellitus (T1D) is one of the most common endocrine conditions. It is estimated that approximately 415 million adults (5-15% type 1 diabetes) and 520,000 children (95% type 1 diabetes) worldwide suffer from diabetes (1). Despite the availability of therapeutic options such as self-monitoring of blood glucose (SMBG), structured education, rapid-acting insulin analogues and insulin pump therapy, glycaemic control in the majority of people with Type 1 diabetes remains suboptimal (2) and they therefore remain prone to complications associated with high glucose levels, such as kidney failure and blindness (3). In England less than one third of people with type 1 diabetes achieve a HbA1c level <7.5% (4). Studies have shown a strong relationship between the frequency of SMBG and HbA1c, with the National Institute of Clinical Excellence recommending 4-10 checks per day (5, 6). However due to pain, inconvenience and the limited information a moment-in-time glucose value provides, finger-stick glucose monitoring remains a key barrier in achieving near normal glucose levels.

In 2014, the FreeStyle Libre Flash Glucose Monitoring System (FSL) (Abbott Diabetes Care, Oxon, UK) became available as a potential alternative to SMBG. The sensor utilises wired enzyme technology (7) to continuously measure interstitial glucose levels. The arm-worn sensor is scanned using a reader or mobile phone app and provides information on current and previous glucose levels and trends. The IMPACT randomised controlled multicentre European trial was the largest study to evaluate the FSL (8) in 328 participants with well-controlled (HbA1c <= 7.5%, 59 mmol/mol) Type 1 diabetes, a third of whom used CSII therapy. FSL use was associated with improvement in a range of glucose-related outcomes: a 38% reduction in time spent in hypoglycaemia (<3.9 mmol/l). There was an increase in glucose time in range but HbA1c was unchanged. The impact of FSL was also assessed in those with Type 2 diabetes on intensive insulin therapy in a large multi-centre randomised European study of 224 participants (9). Time in hypoglycaemia (<3.9mmol/l) reduced compared with controls by 43% but HbA1c was unchanged. In both randomised controlled trials treatment satisfaction was higher in FSL users and no device-related serious adverse events were reported, suggesting that flash glucose monitoring also offers a safe replacement to SMBG in those with diabetes on intensive insulin therapy.

Subsequently a range of observational studies have demonstrated benefits of FSL use for HbA1c and hypoglycaemia (10-15). Campbell et al. evaluated the use of FSL as a replacement for SMBG in young people (4-17 years) (n=76, 58% CSII users, mean (SD) age 10.3(4.0) years, baseline HbA1c 7.9 (1.0)% (63 mmol/mol), T1D duration 5.4(3.7) years) with Type 1 diabetes in a single-arm European multi-centre trial (16). After 2 weeks' baseline masked wear, participants used FSL for 8 weeks. HbA1c significantly improved vs. baseline, -0.4±0.6%, p<0.0001. However, a subsequent 6-month randomised

For peer review only - http://bmjopen.bmj.com/site/about/guidelines.xhtml

controlled parallel-arm trial of the FSL in 64 participants aged 13-20 years with type 1 diabetes and glycated haemoglobin (HbA_{1c}) ≥9% (≥75 mmol/mol) demonstrated no statistically significant difference between groups for changes in HbA_{1c} at 6 months (adjusted mean -0.2% greater improvement for FSL [95% CI -0.9 to 0.5] [-2.1 mmol/mol (95% CI -9.6 to 5.4)]; p= 0.58) (17). The discrepancy in findings between the observational and randomised controlled trials in the paediatric population highlights the importance of high-quality randomised trials to investigate the benefits of this technology. To date, although some are planned, there has been no published randomised controlled trial to demonstrate the impact of the FSL in adults with Type 1 diabetes and high HbA1c levels (18,19).

More recently, in 2020, the Freestyle Libre 2 (FSL2) launched in Europe. This is very similar to FSL except for an optional additional alarm to alert users when the glucose level falls outside their target range, in addition to improved senor accuracy (20). The FSL2 was launched in the United Kingdom in January 2021. To the best of our knowledge no randomised controlled trials to assess the efficacy of the FSL2 system has been conducted or in progress. No economic evaluation assessing relative costs and benefits has been carried out in this patient group, nor has there been an assessment of patient acceptability. The purpose of the Flash UK study is to address this gap in the evidence and determine whether use of flash glucose monitoring with the FSL2 device will improve HbA1c over a 24-week randomised period compared to SMBG in adults with type 1 diabetes and sub-optimal glycaemic control.

Methods and analysis

Trial design

Flash-UK is an open, multi-centre, randomised (1:1), parallel-group superiority trial, in adults and adolescents (16 years and older) with type 1 diabetes and sub-optimal glycaemic control (HbA1c 7.5% to 11% (59 to 97 mmol/mol), either on continuous subcutaneous insulin infusion (CSII) or multiple daily injections (MDI), contrasting flash glucose monitoring using the FSL2 device with traditional finger-stick SMBG for 24 weeks. The study flow chart is outlined in Figure 1.

Study setting

Eight (one primary and seven secondary) care diabetes services from across England, UK.

1. Diabetes Centres within Manchester University Foundation Trust
2. Diabetes Centres within University Hospitals of Derby and Burton NHS Foundation Trust

For peer review only - http://bmjopen.bmj.com/site/about/guidelines.xhtml

3. University Hospitals Birmingham NHS Foundation Trust
4. Wolfson Adult Diabetes Endocrine Clinic, Cambridge Universities Hospitals, Cambridge
5. Norfolk and Norwich University Hospital, Norwich
6. Queen Alexandra Hospital, Portsmouth
7. Ipswich Hospital, East Suffolk and North Essex NHS Foundation Trust, Ipswich
8. Wareham Surgery (Wareham) and The Adam Practice (Poole), NHS England Primary Care GP Practices

Eligibility criteria

The major eligibility criteria are age ≥16 years with Type 1 diabetes for at least 1 year and a HbA1c between 7.5 and 11%. The full criteria are available in Box 1.

Interventions

FreeStyle Libre 2

This intervention is the CE marked FreeStyle Libre 2 (FSL2) flash glucose monitoring device (Abbott Diabetes Care, Oxon, UK). The FSL2 glucose sensor is an arm worn sensor intended to last for 14 days. The component not directly attached to the patient is the handheld reader and/or mobile phone app which display current and historical glucose data. Education and training about insertion and initiation of the sensor as well as how to use flash-glucose monitoring data for treatment optimisation is also provided. Encouragement is also provided to download data at home to identify pattern recognition. This session, designed to meet the needs of the individual, is conducted by a professional diabetes educator or a member of the study team.

Finger-stick SMBG

This is continuation of usual treatment. Additionally, encouragement will be given to use finger-stick glucose levels to optimise therapy and education about insulin dose adjustments using finger-stick glucose levels will also be provided. Participants in both arms will also receive training on sick day rules and dealing with hypo and hyperglycaemia as required.

Participants will be provided with an information leaflet following the training session, although the information provided will be tailored to the respective intervention. The leaflet provided in conjunction with the FSL2 will include sign-posting to educational videos provided by the Association of British Clinical Diabetologists (ABCD) (https://abcd.care/dtn/education) and Bertie online (www.bertieonline.org.uk) in conjunction with the finger-stick SMBG.

For peer review only - http://bmjopen.bmj.com/site/about/guidelines.xhtml

Outcomes

The FLASH-UK study is designed to assess an extensive array of clinical, psychosocial and usability outcomes (Table 1). An outline of a dedicated health economic evaluation is provided below. The primary end point is HbA1c level at 24 weeks post-randomisation. Sensor based outcomes comparing the FSL2 with SMBG will be assessed for the last 2 weeks of the intervention period. Prespecified sensor based outcomes include time in the target range (3.9 to 10mM), duration of hypoglycaemia < 3.5 mmol/l (63 mg/dl); < 3.0 mmol/l (54mg/dl); < 2.8 mmol/l (50 mg/dl), duration of hyperglycaemia (>10 mmol/l (180 mg/dL), and >16.7mM (>300 mg/dL), and glucose variability (Standard deviation & coefficient of variation). All the sensor based metrics will also be analysed separately for daytime (7:00-23:00 hours) and night-time (23:00-7:00 hours) in addition to 24 hour period. Insulin usage data and change from the baseline will be compared between the two arms. Harms outcomes include the frequency of severe hypoglycaemic episodes as defined by American Diabetes Association, Frequency of significant ketosis events (plasma ketones >3mmol/l) and nature and severity of other adverse events. Questionnaire data as outlined in the Table 1 will be collected at baseline and study end.

Participant timeline & data collection

The study will consist of 6 visits for those in the FSL2 arm and 7 visits in the SMBG arm. Visits 1 and 2 can be conjoined. The study flow chart is shown in Figure 1. Key activities undertaken during each study visit are shown in Table 2.

Due to the COVID-19 pandemic all study visits can be conducted either face to face or virtually (supported by telephone and videoconferencing as appropriate) as indicated in the approved study advertisement and participant information sheet. At visit 1, following informed written consent by trained members of the research team, medical and diabetes history will be recorded including presence of diabetes complications, hypoglycaemia burden including the use of Clarke and Gold questionnaires, ethnicity, body weight and height measurement; demographic data, insulin therapy, occupation and educational attainment, any history of disordered eating or needle phobia, previous participation in structured education, carbohydrate counting status, use of bolus calculator and patient self-report psychosocial questionnaires will be completed. Blood samples will be taken to measure HbA1c.

At visit 2 the FreeStyle Libre Pro blinded CGM device will be inserted, to be worn for 2 weeks. For participants on the virtual pathway, participant will be taught to self insert the sensor. At Visit 3, participant adherence / tolerance of using the flash-CGM over the preceding 14 days will be assessed.

For peer review only - http://bmjopen.bmj.com/site/about/guidelines.xhtml

To proceed to randomisation at least 10 of 14 days blinded CGM data must be available. Those who have <10 days data will be provided with a new FreeStyle Libre pro sensor and reader to obtain the minimum data requirements prior to randomisation or discontinue from trial participation if there is a continued adverse reaction to the sensor adhesive.

Participants will be followed up at 4, 12 and 24 weeks post randomisation (+/- 2 weeks). At these visits glucose data will be downloaded and participant diaries which collect information on insulin doses and carbohydrate intake in last 5 days will be collected. Participants in the virtual pathway will be encouraged to upload data from home. Blood tests for HbA1c will also be performed at 12 and 24 weeks after randomisation. Those in the SMBG group will attend an additional visit at 22 weeks to have the blinded CGM sensor fitted to be worn from 22-24 weeks. At 24 weeks, body weight measurement will be made where possible and the participant will be asked to complete questionnaires. In addition, participants in the FSL2 arm will be asked to complete a questionnaire exploring expectations and experience of using FSL2 during the study. Healthcare resource use in primary and secondary healthcare settings will also be collected at 24 weeks.

Adverse events will be routinely collated at study visits. The independent data monitoring committee (IDMC) will be informed of all serious adverse events and any unanticipated adverse device effects that occur during the study and will review compiled adverse event data at periodic intervals.

Sample size

The target effect size (minimally clinically important HbA1c difference) of 0.4% was chosen as this is consistent with other relevant trials (0.5% in REPOSE (21), 0.4% in DIAMOND (22), 0.3% in GOLD (23)). To achieve 90% power using an independent-sample t-test for 24-week HbA1c values (2-tail alpha=0.05, power=0.80, effect size=0.4%, SD=0.8% (23) i.e. Standardised Effect Size = 0.5), 128 participants with primary outcome data are required. This is inflated to a target of 150 randomised (assuming maximum 15% attrition) and up to 180 recruited (to allow for pre-randomisation losses). The use of ANCOVA with adjustment for baseline values of HbA1c and other minimisation factors should increase power.

Recruitment

Participants will be identified by treating clinicians in each centre and participant information sheet provided. The first study site was activated on the 20/12/2019 and first participant was recruited on the 09/01/2020. Each site will each aim to recruit between 14 and 30 participants up to a total of 180 participants. The study has been advertised via social media and through the official trial website

For peer review only - http://bmjopen.bmj.com/site/about/guidelines.xhtml

(https://sites.manchester.ac.uk/flash/), providing those who do not usually attend study centres to have the opportunity to participate. As of 31/01/2021, 109 participants had been randomised. It is anticipated that recruitment will be completed by 30/04/2021.

Allocation

Sequence generation

Allocation to one of the two intervention arms (24-weeks use of flash-glucose monitoring or 24 weeks use of conventional finger-stick SMBG) will use stochastic minimisation (factors: study centre, baseline HbA1c (7.5%-9.0%; >9.0%-11%), treatment modality (MDI;CSII), prior participation in structured education course (yes; no) and current use of bolus calculator (yes; no)).

Allocation concealment mechanism and its implementation

Allocation will be implemented using the web-based Sealed Envelope™ software (Sealed Envelope Ltd, London, UK) randomisation system. This will be independently managed by Manchester CTU (MCTU) staff. Participants will have their pseudonymised registration details entered into the randomisation system by delegated recruitment site research teams who will also confirm participant eligibility; only then will the system generate an email confirmation with their allocation.

Blinding (masking)

The trial is fully open. It is not possible to blind investigators or participants to the delivery or receipt of the intervention; data collectors and statisticians are also unblinded to treatment allocations.

Laboratory analysis

Blood samples for the measurement of HbA1c levels will be taken at three different time points: screening, 12 and 24 weeks. This can be completed using the local laboratory (face to face clinic) or at home where the participant will collect a blood sample using a home self-test kit (TDL TINY™) and send in a pre-paid envelope to The Doctors Laboratory Ltd (London, UK).

Data management

Confidentiality of participant data shall be observed at all times during the study. Personal details for each participant taking part in the research study and linking them to a unique identification number will be held locally on a study screening log in the Investigator Site File at each of the investigation centres. The study identification number will be used on the case report forms,on all the blood samples that are collected throughout the study and FreeStyle Libre 2 and FreeStyle Libre Pro data submitted to study specific Libreview database (Libreview.com). Names and full addresses will not be

For peer review only - http://bmjopen.bmj.com/site/about/guidelines.xhtml

used. Electronic data will be stored on password-protected computers. Only members of the research team and collaborating institutions will have password access to the anonymised electronic data. Paper copies of the data will be stored for 15 years. Direct access to the source data will be provided for monitoring, audits, REC review during and after the study. The fully anonymised data may be shared with third parties (EU or non-EU based) for the purposes of advancing management and treatment of diabetes. Standard procedures agreed by the MCTU, Chief Investigator and Clinical Principal Investigators are in place for data review, database cleaning and issuing and resolving data queries.

Analysis

Statistical Methods

All efficacy and safety analyses will be conducted following the intention to treat (ITT) principle in which all randomised participants are analysed in their allocated treatment group whether or not they receive their randomised treatment. All baseline, 12-week and 24-week outcome data will be presented descriptively, both overall and within treatment group, using mean (SD), median (IQR) or frequency (percentage), as appropriate. All statistical tests will use a 2-sided significance level of 5% (unless otherwise specified). All confidence intervals will be presented at a level of 95% and will be two sided. All statistical analyses will be performed using Stata (StataCorp, College Station TX, USA).

The primary outcome analysis will evaluate between group differences in HbA1c levels at the end of the 24-week treatment period. An analysis of covariance (ANCOVA) model will be used, with 24-week HbA1c as the outcome and trial arm effect as the focus, and with adjustment for baseline HbA1c, the other baseline variables included in the minimisation allocation algorithm, and other prognostic factors (sex, age, duration of diabetes, BMI and deprivation index quintile) as covariates. If more than 10% HbA1c data are missing at 24 weeks (or a >10% difference between missing data percentages in the two arms) multiple imputation will be used in order to implement a more complete ITT analysis of the substantive ANCOVA model (otherwise this will be performed as a sensitivity analysis, with a complete-case analysis used as the primary analysis). The imputation model will include baseline and 12-week HbA1c, all the baseline variables used in the outcome model and any other recorded variables found to be predictive of missingness in the 24-week outcome in exploratory analyses (via a logistic regression model, with terms included using a 10% significance level). Sensitivity analyses will be performed to (i) examine robustness of how missing data are handled, (ii) examine impact of data collected outside the visit window, and (iii) examine potential impact of Covid-19 on the primary outcome.

For peer review only - http://bmjopen.bmj.com/site/about/guidelines.xhtml

Quantitative secondary outcomes will also be analysed using ANCOVA and binary secondary outcomes will be analysed using logistic regression. In each case, the analysis will be adjusted for the baseline value of the outcome and the adjustment factors used in the model for HbA1C. Harms data will be reported descriptively as frequencies and percentages (%), both overall and by intervention arm.

Subgroup analysis will be performed by the addition of interactions between intervention arm and the subgrouping factor. All subgroup analyses will be performed separately. Planned subgroups are: baseline HbA1c; treatment modality; prior participation in structured education course; age group; education level; hypoglycaemia unawareness; deprivation index quintile; sex; ethnic group.

Further sensitivity and/or subgroup analyses will be performed as appropriate. These together with fuller details of the analyses proposed above, and any additional analyses, will be included in a full Statistical Analysis Plan that will be approved by the Trial Steering Committee (TSC) prior to any analysis of the outcome data.

Economic analysis

The economic evaluation will determine the difference in costs and outcomes generated by the FSL2 device compared with SMBG. The economic evaluation will be conducted from the perspective of NHS/Personal Social Services (PSS) following standard quality design and reporting criteria (24).

A within-trial cost-utility analysis will compare differences in total costs and differences in quality of life using QALYs derived from the EQ-5D-5L. QALYs will be calculated by attaching available utility weights to the health states generated from the EQ-5D-5L, using area under the curve methods with an assumption of a linear change between time points, controlling for baseline. Person-level costs will be generated for each person in the FSL2 device and SMBG arms from a combination of trial-based resource use with published unit costs, allowing comparison in terms of costs to NHS and PSS (25). Costs will be compared between the two groups using non-parametric methods.

Modelling the potential effect of the intervention on costs and outcomes beyond the trial period will provide a better idea of overall impact as the benefits of controlling HbA1c are likely to be seen after the endpoint of the trial. Therefore, we will carry out an economic evaluation informed by modelling to estimate longer-term benefits and costs, from an NHS/PSS perspective.

A commercially available cost-effectiveness model, the IMS Centre for Outcomes Research and Effectiveness diabetes model version 8.5 (IMS Health, Danbury, CT, USA), will be used for this economic evaluation. The model consists of 15 submodels designed to simulate diabetes-related

For peer review only - http://bmjopen.bmj.com/site/about/guidelines.xhtml

complications, non-specific mortality and costs over time. It also incorporates the costs and effects of hypoglycaemia, so is particularly well-suited to this study. Two major validation papers on the IMS CDM have been published to date. (26,27) The IMS Core Diabetes Model has also been used in a UK-based recent health technology assessment of CGM commissioned by NICE.(28)

Incremental cost-effectiveness ratios (ICERs) will be calculated in the event of the intervention having either higher costs and better outcomes or lower costs and worse outcomes (no scenarios of dominance, based on QALYs and trial primary outcome). The base case analysis will estimate the mean costs and QALYs across the treatment arms. The overall impact of uncertainty will be assessed by generating cost-effectiveness planes from bootstrapped resamples, and distributional assumptions about the transition probabilities, costs and utility values will be made. Cost-effectiveness acceptability curves (CEACs) will be constructed to show the probability that the intervention is cost-effective for different willingness to pay (WTP) thresholds. Incremental economic analysis using the IMS CORE model will require the model's time horizon to be set to 80 years, which approximates to a lifetime horizon. All costs and effects will be discounted by 3.5%, as per NICE guidance(25,29).

Process evaluation

The process evaluation will be undertaken to explain discrepancies between expected and observed outcomes, to understand how context influences outcomes, and to provide insights to aid implementation. Specifically, we will investigate whether treatment is consistent with the behaviour change theories, which underpin it and contextual factors have affected implementation. Process evaluation will use a pipeline logic model, showing causal links between resources, activities and outcomes, integrating the National Institute for Health Behaviour Change Consortium's (NIHBCC's) approach to treatment fidelity (30) and a modified version of Linnan and Steckler's framework for process evaluation.(31) We will describe context qualitatively and take a mixed methods approach to characterising recruitment, reach, dose delivered/received and fidelity, with triangulation between data sources.(32) Free-text response questionnaires will be completed by intervention designers, health professionals and trial participants, and analyses combined with trial data, including FSL2 device utilization and SMBG data which will be analysed descriptively (including the use of appropriate graphical representation), within arms where appropriate, will be synthesised and findings triangulated appropriately.

Study Management

Trial Management Group (TMG)

For peer review only - http://bmjopen.bmj.com/site/about/guidelines.xhtml

For peer review only

TMG comprising the Chief Investigator, Co-Investigators, Trial Managers, Trial Statistician, Trial Health Economist, Monitor, and Data Manager will meet quarterly to discuss the operational aspects of the study.

Trial Steering Committee

TSC with an independent chair has been appointed. Independent membership of the TSC includes two clinical members (including the chair), two service users, health economist and statistician; non-independent members are Chief investigator (CI) and Statistician Co-Investigator (only Statistician voting). Other members of the study team including the Sponsor are invited to TSC meetings as observers only.

Independent Data Monitoring Committee

An IDMC comprising a clinical chair, another clinical expert and a statistician has been appointed. The IDMC will be informed of all serious adverse events and any unanticipated adverse device effects/events that occur during the study. The IDMC will review compiled adverse event data at periodic intervals. The IDMC will report to the TSC any safety concerns and recommendations for suspension or early termination of the investigation.

Study Monitoring

A detailed risk assessment was completed by MCTU and approved by the Sponsor. The procedures, source data transfer modalities and anticipated frequency for monitoring are documented in the Monitoring Plan. Copies of the risk assessment and the Monitoring Plan will be stored in the Trial Master File. The MCTU study monitor will be fully independent of both the Sponsor and Site Principal Investigators.

Authorised representatives of Sponsor, regulatory authority, or an Ethics Committee may perform audits or inspections at the recruiting centres, including source data verification.

Patient and public involvement

During the grant application process the study protocol received input from several patient groups from Manchester, Derby and Cambridge as well as the INPUT patient advocacy group. One of the study investigators also has Type 1 diabetes as do two members of the Trial Steering Committee.

Ethics and dissemination

For peer review only - http://bmjopen.bmj.com/site/about/guidelines.xhtml

The study will be conducted in accordance with the Declaration of Helsinki Ethical Principles for Medical Research involving Human Subjects (October 2000). It was approved by Greater Manchester West Research Ethics Committee on 21/03/2019REC reference 19/NW/0081. All participants will be provided with oral and written information about the trial, including the most common AEs, and the procedures involved in the study before obtaining written informed consent. The study results will be communicated to trial participants and disseminated in peer-review publications and through conference presentations.

For peer review only

For peer review only - http://bmjopen.bmj.com/site/about/guidelines.xhtml

Box 1. Key Inclusion and Exclusion Criteria

**Inclusion Criteria**
1. The participant is ≥16 years old<br>2. The participant has type 1 diabetes, as defined by WHO for at least 1 year or is confirmed C-peptide negative if duration of diabetes is < 1 years<br>3. Participant is treated with insulin pump or multiple daily injection for at least 12 weeks and no plans to change treatment modality during next 28 weeks<br>4. The participant is literate in English for safe study conduct<br>5. Screening HbA1c ≥ 7.5% (58.5mmol/mol) and ≤ 11% (97 mmol/mol) based on analysis from local, central or third party external laboratory<br>6. The participant is willing to wear study glucose sensor and scan for glucose levels at regular intervals,<br>7. The participant is willing to follow study specific instructions and improve glucose control<br>8. Female participants of child bearing age should be on effective contraception or not sexually active / no plans for pregnancy
**Exclusion Criteria**
1. Non-type 1 diabetes mellitus including those secondary to chronic disease<br>2. Any other physical disease or people with known severe mental illness (psychotic disorder, bipolar disorder, dementia, substance and alcohol dependence, learning disabilities, depression with active suicidal ideation) which are likely to interfere with the normal conduct of the study and interpretation of the study results as judged by the investigator<br>3. Current users of real-time glucose monitoring sensors or flash-glucose monitoring for more than 4 weeks within last 12 weeks<br>4. Initiation of medications/treatments known to interfere with glucose metabolism (e.g: metformin, SGLT2 inhibitors, GLP-1 agonists, Pramlinatide) within the last 6 weeks or planning to start these medications within the next 6 months (patients on stable treatment is not an exclusion) or current or planned glucocorticoid use other than inhaled/ topical use<br>5. Known or suspected allergy against insulin<br>6. Severe visual impairment<br>7. Complete loss of hypoglycaemia awareness<br>8. Patient receiving dialysis / pre-dialysis based on history<br>9. More than one episode of severe hypoglycaemia as defined by American Diabetes Association (30) in preceding 24 weeks<br>10. Pregnancy, Planned Pregnancy in the next 8 months or breast feeding

For peer review only

For peer review only

Table 1: Secondary Outcomes

Category	Outcomes	Assessment Time-point
**HbA1c based**	HbA1c<br>HbA1c ≤ 53 mmol/mol (7.0%) [yes/no];<br><br>HbA1c ≤ 59 mmol/mol (7.5%) [yes/no];<br>Reduction in HbA1c ≥5.5 mmol/mol (0.5%) from baseline [yes/no];<br>Reduction in HbA1c ≥ 11 mmol/mol (1.0%) from baseline [yes/no];	Baseline and 12 weeks<br>Baseline, 12 weeks and 24 weeks<br>Baseline, 12 weeks and 24 weeks<br>Baseline, 12 weeks and 24 weeks<br>Baseline, 12 weeks and 24 weeks
**Sensor based (glucose)**	Time spent in the target glucose range 3.9 to 10.0 mmol/l (70 to 180mg/dl),<br>Time spent below target glucose (<3.9mmol/l) (<70mg/dl), < 3.5 mmol/l (63 mg/dl); < 3.0 mmol/l (54mg/dl); < 2.8 mmol/l (50 mg/dl)<br>Time spent above target glucose (10.0 mmol/l) (180 mg/dl), > 16.7 mmol/l) (300mg/dl).<br>Average glucose , Standard deviation, Coefficient of variation<br>AUC of glucose below 3.0mmol/l (54mg/dl)	Baseline and 24 weeks
**Non-sensor based (clinical)**	Daily average total insulin dose<br>Daily average basal insulin dose<br>Daily average bolus dose<br>Average number of boluses of rapid acting insulin per day<br>Frequency of severe hypoglycaemic episodes as defined by American Diabetes Association<br>Frequency of significant ketosis events (plasma ketones >3mmol/l)<br>Nature and severity of other adverse events	Baseline, 12 weeks and 24 weeks
**Psychosocial**	Type 1 Diabetes Distress Scale (T1-DDS)<br>Quality of Life (EQ-5D-5L)<br>Patient Health Questionnaire (PHQ-9)<br>Diabetes fear of injecting and self-testing questionnaire (D-FISQ)<br>The revised Diabetes Eating Problem Survey (DEPS-R)	Baseline and 24 weeks
**Process evaluation (utility and acceptability)**	FSL2 device utilization data, including: average number of scans per day (7:00-23:00 hours), per night (23:00-7:00 hours) and over the full 24-hour period; average number of days of usage per week for the full 24 week intervention<br><br>Number of finger-stick glucose level tests per day<br><br>Diabetes Treatment Satisfaction Questionnaire (DTSQ)<br>Glucose Monitoring Satisfaction Survey (GMSS)	Continuous (FSL arm only)<br><br><br><br>Continuous<br><br>Baseline and 24 weeks

For peer review only

For peer review only - http://bmjopen.bmj.com/site/about/guidelines.xhtml

Table 2: Schedule of study visits:

**Visit/ contact**	**Description**	**Time since randomisation**	**Start relative to previous / next Visit / Activity****
Visit 1	Recruitment & Screening visit: Consent<br>HbA1c, baseline bloods, baseline questionnaires	-2 to -3 weeks	-
Visit 2	Blinded flash glucose monitor insertion	-2 weeks	Within 1 to 2 weeks of Visit 1. Can coincide with Visit 1
Visit 3	Adherence assessment &<br>Randomisation<br>FSL2 / Self- monitoring of glucose initiation<br>- Education	0 weeks	After 2 weeks of Visit 2
Visit 4	Review data /optimisation<br>Collect participant diary	+4 weeks	After 4 weeks of Visit 3
Visit 5	Review data /optimisation. Data download<br>- HbA1c<br>- Collect participant diary	+12 weeks	After 8 weeks of Visit 5
Visit 6	Blinded flash glucose monitor insertion (Extra visit in SMBG arm)	+22 weeks	After 10 weeks of Visit 5
Visit 7	End of self-monitoring intervention arm<br>- HbA1c.<br>- Questionnaires<br>- Collect participant diary	+24 weeks	2 weeks after Visit 6

For peer review only - http://bmjopen.bmj.com/site/about/guidelines.xhtml

Authors' contributions

LL, EW and MLE conceptualised the study. LL, EW, MLE, IC, PN, SN, RE, CS, HT contributed to the grant application. MB is the Lead Clinical Trial Manager. CS, AK and VT are responsible for statistical analysis. All authors contributed to protocol development at various stages. LL, EW, MLE, IC, PN, SN, HT, GR, SL provides site oversight and are responsible for study conduct at each site. RE & GG are responsible for the health economic analysis. KB is responsible for the process evaluation. EGW & LL wrote the first draft of the manuscript and all authors reviewed and had the opportunity to comment on the content prior to submission.

Competing interests statement

EGW has received personal fees from Abbott Diabetes Care, Dexcom, Eli Lilly, Insulet, Medtronic, Novo Nordisk, Sanofi Diabetes Care. LL has received personal fees from Abbott Diabetes Care, Dexcom, Insulet, Medtronic, Novo Nordisk, Sanofi Diabetes Care. ME has received personal fees from Abbott Diabetes Care, Eli Lilly, Medtronic, Novo Nordisk, Astra Zeneca, Zucara.SN has received personalised fees from QUIN,Roche. NK has received personal fees from Abbott,Eli Lilly,Novo Nordisk, Astra Zeneca, Napp, Sanofi. PN has acted as a clinical expert for NICE Medtech innovation briefing MIB110 relating to FreeStyle Libre system. GR has received lecture and consultancy fees from Abbott Diabetes UK. MB, KBK, GG, CS, CK, VT, AK, SN, RAE-No competing interests.

Funding statement

This work was supported by Diabetes UK grant number 18/0005836. The device manufacturer played no part in design, conduct or any other aspects of the study. The study devices were paid by the National Health Service (NHS) UK. Work was supported by the NIHR Cambridge Biomedical Research Centre. The University of Cambridge has received salary support for MLE from the National Health Service in the East of England through the Clinical Academic Reserve.

For peer review only

For peer review only - http://bmjopen.bmj.com/site/about/guidelines.xhtml

References

1. International Diabetes Federation, The Diabetes Atlas, Seventh Edition. Brussels:2015. http://www.diabetesatlas.org/, 2015
2. McKnight JA, Wild SH, Lamb MJ, Cooper MN, Jones TW, Davis EA, Hofer S, Fritsch M, Schober E, Svensson J, Almdal T, Young R, Warner JT, Delemer B, Souchon PF, Holl RW, Karges W, Kieninger DM, Tigas S, Bargiota A, Sampanis C, Cherubini V, Gesuita R, Strele I, Pildava S, Coppell KJ, Magee G, Cooper JG, Dinneen SF, Eeg-Olofsson K, Svensson AM, Gudbjornsdottir S, Veeze H, Aanstoot HJ, Khalangot M, Tamborlane WV, Miller KM: Glycaemic control of Type 1 diabetes in clinical practice early in the 21st century: an international comparison. Diabet Med 2015;32:1036-1050
3. Nathan DM, Cleary PA, Backlund JY, Genuth SM, Lachin JM, Orchard TJ, Raskin P, Zinman B, Diabetes Control and Complications Trial / Epidemiology of Diabetes Interventions and Complications (DCCT/EDIC) Study Research Group: Intensive diabetes treatment and cardiovascular disease in patients with type 1 diabetes. N Engl J Med 2005;353:2643-2653
4. NHS Digital: National Diabetes Audit - 2015-2016: Report 1, Care Processes and Treatment Targets. http://digitalnhsuk/catalogue/PUB23241 2017;
5. Miller KM, Beck RW, Bergenstal RM, Goland RS, Haller MJ, McGill JB, Rodriguez H, Simmons JH, Hirsch IB, Network TDEC: Evidence of a Strong Association Between Frequency of Self-Monitoring of Blood Glucose and Hemoglobin A1c Levels in T1D Exchange Clinic Registry Participants. Diabetes Care 2013;36:2009-2014
6. NICE guidance NG17. Type 1 diabetes in adults: diagnosis and management https://www.nice.org.uk/guidance/ng17 lst accessed 24/2/21
7. Danne T, de Valk HW, Kracht T, Walte K, Geldmacher R, Sölter L, von dem Berge W, Welsh ZK, Bugler JR, Lange K, Kordonouri O: Reducing glycaemic variability in type 1 diabetes self-management with a continuous glucose monitoring system based on wired enzyme technology. Diabetologia 2009;52:1496-1503 *FLASH-UK Protocol Version 4.0 29th June 2020: IRAS No.: 257593 Page 110 of 111*
8. Bolinder J, Antuna R, Geelhoed-Duijvestijn P, Kroger J, Weitgasser R: Novel glucose-sensing technology and hypoglycaemia in type 1 diabetes: a multicentre, non-masked, randomised controlled trial. Lancet 2016;388:2254-2263
9. Haak T, Hanaire H, Ajjan R, Hermanns N, Riveline JP, Rayman G: Use of Flash Glucose-Sensing Technology for 12 months as a Replacement for Blood Glucose Monitoring in Insulin-treated Type 2 Diabetes. Diabetes therapy : research, treatment and education of diabetes and related disorders 2017;8:573-586
10. Dover AR, Stimson RH, Zammitt NN, Gibb FW: Flash Glucose Monitoring Improves Outcomes in a Type 1 Diabetes Clinic. J Diabetes Sci Technol 2017;11:442-443
11. McKnight JA, Gibb FW: Flash Glucose Monitoring is associated with improved glycaemic control but use is largely limited to more affluent people in a UK diabetes centre. Diabet Med 2017;34:732
12. Ish-Shalom M, Wainstein J, Raz I, Mosenzon O: Improvement in Glucose Control in Difficult-to-Control Patients With Diabetes Using a Novel Flash Glucose Monitoring Device. J Diabetes Sci Technol 2016;10:1412-1413
13. Leelarathna L, Wilmot EG. Flash forward: a review of flash glucose monitoring. Diabet Med. 2018 Apr;35(4):472-482. doi: 10.1111/dme.13584. Epub 2018 Feb 27.
14. Deshmukh H, Wilmot EG, Gregory R, Barnes D, Narendran P, Saunders S, Furlong N, Kamaruddin S, Banatwalla R, Herring R, Kilvert A, Patmore J, Walton C, Ryder REJ, Sathyapalan T. Effect of Flash Glucose Monitoring on Glycemic Control, Hypoglycemia, Diabetes-Related Distress, and Resource Utilization in the Association of British Clinical Diabetologists (ABCD) Nationwide Audit. Diabetes Care. 2020 Sep;43(9):2153-2160.
15. Rose L, Klausmann G, Seibold A. Improving HbA1c Control in Type 1 or Type 2 Diabetes Using Flash Glucose Monitoring: A Retrospective Observational Analysis in Two German Centres. Diabetes Ther. 2020 Dec 11. doi: 10.1007/s13300-020-00978-9.
16. Campbell F, Kordonouri O, Murphy N, Stewart C: FreeStyle Libre Use for Self-Management of Diabetes in Children and Adolescents. Diabetes 2017;66 Suppl. 1A:LB28-LB28
17. Boucher SE, Gray AR, Wiltshire EJ, de Bock MI, Galland BC, Tomlinson PA, Rayns JA, MacKenzie KE, Chan H, Rose S, Wheeler BJ. Effect of 6 Months of Flash Glucose Monitoring in Youth With Type 1 Diabetes and High-Risk Glycemic Control: A Randomized Controlled Trial. Diabetes Care. 2020 Oct;43(10):2388-2395. doi: 10.2337/dc20-0613.
18. Secher AL, Pedersen-Bjergaard U, Svendsen OL, Gade-Rasmussen B, Almdal TP, Dørflinger L, Vistisen D, Nørgaard K. Study protocol for optimising glycaemic control in type 1 diabetes treated with multiple daily

For peer review only - http://bmjopen.bmj.com/site/about/guidelines.xhtml

insulin injections: intermittently scanned continuous glucose monitoring, carbohydrate counting with automated bolus calculation, or both? A randomised controlled trial.
BMJ Open. 2020 Apr 27;10(4):e036474.
19. Zhou Y, Deng H, Liu H, et al. Effects of novel flash glucose monitoring system on glycaemic control in adult patients with type 1 diabetes mellitus: protocol of a multicentre randomised controlled trialBMJ Open 2020;10:e039400. doi: 10.1136/bmjopen-2020-039400
20. Alva S, Bailey T, Brazg R, Budiman ES, Castorino K, Christiansen MP, Forlenza G, Kipnes M, Liljenquist DR, Liu H. Accuracy of a 14-Day Factory-Calibrated Continuous Glucose Monitoring System With Advanced Algorithm in Pediatric and Adult Population With Diabetes. J Diabetes Sci Technol. 2020 Sep 19:1932296820958754. doi: 10.1177/1932296820958754. Online ahead of print.#
21. REPOSE Study Group: Relative effectiveness of insulin pump treatment over multiple daily injections and structured education during flexible intensive insulin treatment for type 1 diabetes: cluster randomised trial (REPOSE). BMJ. 2017 Mar 30;356:j1285. doi: 10.1136/bmj.j1285.
22. Beck RW, Riddlesworth T, Ruedy K, Ahmann A, Bergenstal R, Haller S, Kollman C, Kruger D, McGill JB, Polonsky W, Toschi E, Wolpert H, Price D, Group DS: Effect of Continuous Glucose Monitoring on Glycemic Control in Adults With Type 1 Diabetes Using Insulin Injections: The DIAMOND Randomized Clinical Trial. JAMA 2017;317:371-378
23. Lind M, Polonsky W, Hirsch IB, Heise T, Bolinder J, Dahlqvist S, Schwarz E, Olafsdottir AF, Frid A, Wedel H, Ahlen E, Nystrom T, Hellman J: Continuous Glucose Monitoring vs Conventional Therapy for Glycemic Control in Adults With Type 1 Diabetes Treated With Multiple Daily Insulin Injections: The GOLD Randomized Clinical Trial. JAMA 2017;317:379-387
24. Husereau D, Drummond M, Petrou S, Carswell C, Moher D, Greenberg D, Augustovski F, Briggs AH,Mauskopf J, Loder E: Consolidated Health Economic Evaluation Reporting Standards (CHEERS)—Explanation and Elaboration: A Report of the ISPOR Health Economic Evaluation Publication Guidelines Good Reporting. Practices Task Force. Value in Health 2013;16:231-250
25. HM Treasury. The green book: appraisal and evaluation in central government. 2003. TSO: London. 2014.
26. Palmer AJ, Roze S, Valentine WJ, Minshall ME, Foos V, Lurati FM, Lammert M, Spinas GA: Validation of theCORE Diabetes Model against epidemiological and clinical studies. Curr Med Res Opin 2004;20 Suppl 1:S27-40
27. McEwan P, Foos V, Palmer JL, Lamotte M, Lloyd A, Grant D: Validation of the IMS CORE Diabetes Model.Value in Health 17:714-724
28. Riemsma R, Corro Ramos I, Birnie R, Büyükkaramikli N, Armstrong N, Ryder S, Duffy S, Worthy G, Al M, Severens J, Kleijnen J: Integrated sensor-augmented pump therapy systems [the MiniMed® Paradigm™ Veo system and the Vibe™ and G4® PLATINUM CGM (continuous glucose monitoring) system] for managing blood glucose levels in type 1 diabetes: a systematic review and economic evaluation. Health Technology Assessment (Winchester, England) 2016;20:1-252
29. National Institute for Health and Care Excellence. Guide to the Methods of Technology Appraisal. National Institute for Clinical Excellence; 2013.
30. Bellg AJ, Borrelli B, Resnick B, Hecht J, Minicucci DS, Ory M, et al. Enhancing treatment fidelity in health behavior change studies: best practices and recommendations from the NIH Behavior Change Consortium. Health Psychol [Internet]. 2004 [cited 2012 Mar 19];23(5):443–51
31. Linnan L, Steckler A. Process evaluation for public health interventions and research: an overview. In: Linnan L, Steckler A, editors. Process evaluation for public health interventions and research. 1st ed. San Francisco: Jossey-Bass; 2002. p. 1–23.
32. Farmer T, Robinson K, Elliott SJ, Eyles J. Developing and implementing a triangulation protocol for qualitative health research. Qual Health Res. 2006;16(3):377–94.

Figure 1. Flash UK study flow chart

For peer review only - http://bmjopen.bmj.com/site/about/guidelines.xhtml

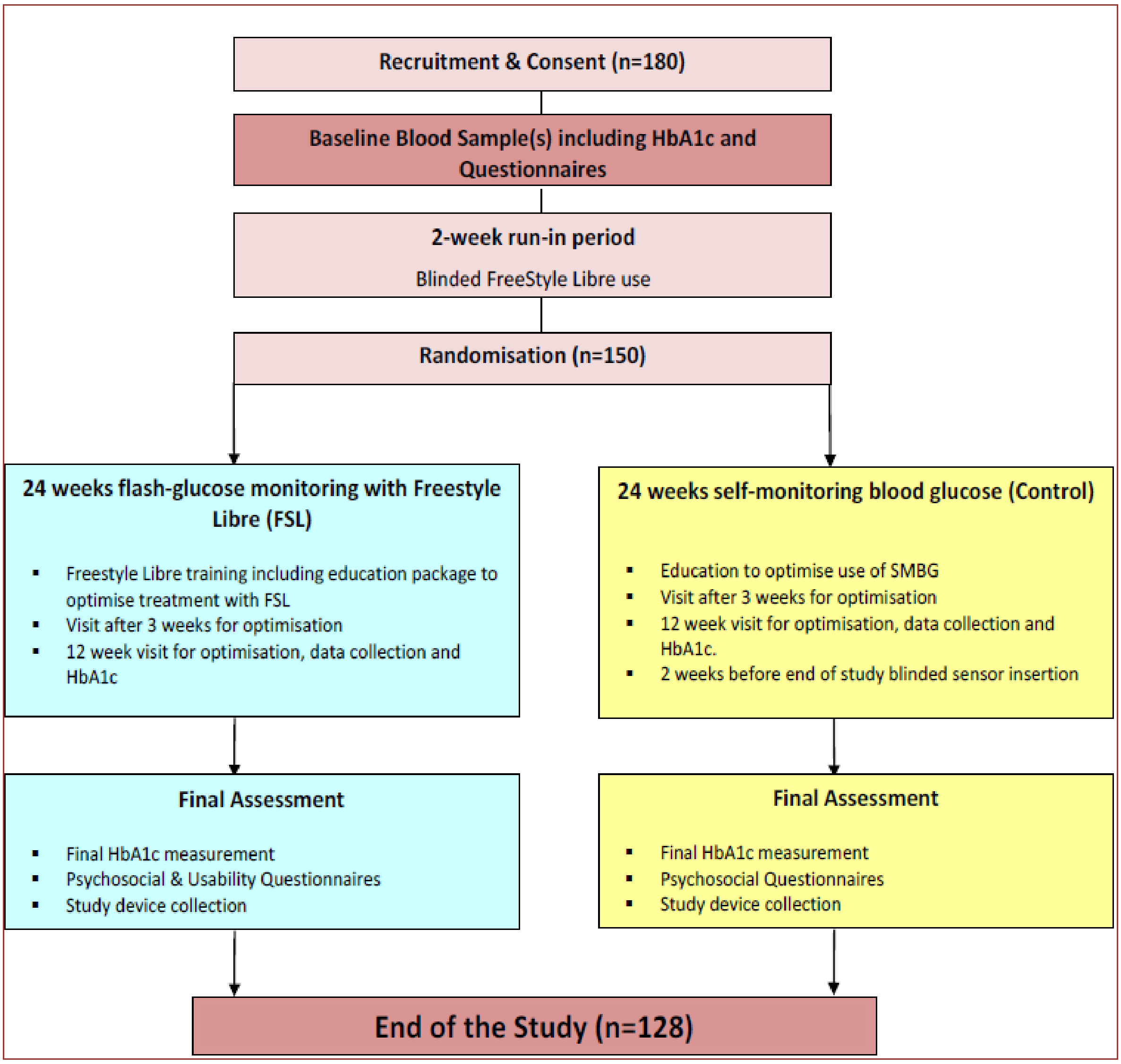
Recruitment & Consent (n=180)
Baseline Blood Sample(s) including HbA1c and Questionnaires
2-week run-in period
Blinded FreeStyle Libre use
Randomisation (n=150)
24 weeks flash-glucose monitoring with Freestyle Libre (FSL)
Freestyle Libre training including education package to optimise treatment with FSL
Visit after 3 weeks for optimisation
12 week visit for optimisation, data collection and HbA1c
24 weeks self-monitoring blood glucose (Control)
Education to optimise use of SMBG
Visit after 3 weeks for optimisation
12 week visit for optimisation, data collection and HbA1c.
2 weeks before end of study blinded sensor insertion
Final Assessment
Final HbA1c measurement
Psychosocial & Usability Questionnaires
Study device collection
Final Assessment
Final HbA1c measurement
Psychosocial Questionnaires
Study device collection
End of the Study (n=128)

only

SPIRIT

STANDARD PROTOCOL ITEMS: RECOMMENDATIONS FOR INTERVENTIONAL TRIALS

SPIRIT 2013 Checklist: Recommended items to address in a clinical trial protocol and related documents*

Section/item	Item No	Description	Page number
**Administrative information**			
Title	1	Descriptive title identifying the study design, population, interventions, and, if applicable, trial acronym	1
Trial registration	2a	Trial identifier and registry name. If not yet registered, name of intended registry	2
	2b	All items from the World Health Organization Trial Registration Data Set	
Protocol version	3	Date and version identifier	3
Funding	4	Sources and types of financial, material, and other support	22
Roles and responsibilities	5a	Names, affiliations, and roles of protocol contributors	3, 21
	5b	Name and contact information for the trial sponsor	3
	5c	Role of study sponsor and funders, if any, in study design; collection, management, analysis, and interpretation of data; writing of the report; and the decision to submit the report for publication, including whether they will have ultimate authority over any of these activities	22
	5d	Composition, roles, and responsibilities of the coordinating centre, steering committee, endpoint adjudication committee, data management team, and other individuals or groups overseeing the trial, if applicable (see Item 21a for data monitoring committee)	14
**Introduction**			
Background and rationale	6a	Description of research question and justification for undertaking the trial, including summary of relevant studies (published and unpublished) examining benefits and harms for each intervention	5-6
	6b	Explanation for choice of comparators	na

For peer review only

Objectives	7	Specific objectives or hypotheses	6
Trial design	8	Description of trial design including type of trial (eg, parallel group, crossover, factorial, single group), allocation ratio, and framework (eg, superiority, equivalence, noninferiority, exploratory)	6
**Methods: Participants, interventions, and outcomes**			
Study setting	9	Description of study settings (eg, community clinic, academic hospital) and list of countries where data will be collected. Reference to where list of study sites can be obtained	6-7
Eligibility criteria	10	Inclusion and exclusion criteria for participants. If applicable, eligibility criteria for study centres and individuals who will perform the interventions (eg, surgeons, psychotherapists)	17
Interventions	11a	Interventions for each group with sufficient detail to allow replication, including how and when they will be administered	7
	11b	Criteria for discontinuing or modifying allocated interventions for a given trial participant (eg, drug dose change in response to harms, participant request, or improving/worsening disease)	9
	11c	Strategies to improve adherence to intervention protocols, and any procedures for monitoring adherence (eg, drug tablet return, laboratory tests)	8,19, table 2
	11d	Relevant concomitant care and interventions that are permitted or prohibited during the trial	17
Outcomes	12	Primary, secondary, and other outcomes, including the specific measurement variable (eg, systolic blood pressure), analysis metric (eg, change from baseline, final value, time to event), method of aggregation (eg, median, proportion), and time point for each outcome. Explanation of the clinical relevance of chosen efficacy and harm outcomes is strongly recommended	8,18
Participant timeline	13	Time schedule of enrolment, interventions (including any run-ins and washouts), assessments, and visits for participants. A schematic diagram is highly recommended (see Figure)	19

For peer review only

For peer review only - http://bmjopen.bmj.com/site/about/guidelines.xhtml

Sample size	14	Estimated number of participants needed to achieve study objectives and how it was determined, including clinical and statistical assumptions supporting any sample size calculations	9
Recruitment	15	Strategies for achieving adequate participant enrolment to reach target sample size	9
**Methods: Assignment of interventions (for controlled trials)**			
Allocation:			
Sequence generation	16a	Method of generating the allocation sequence (eg, computer-generated random numbers), and list of any factors for stratification. To reduce predictability of a random sequence, details of any planned restriction (eg, blocking) should be provided in a separate document that is unavailable to those who enrol participants or assign interventions	10
Allocation concealment mechanism	16b	Mechanism of implementing the allocation sequence (eg, central telephone; sequentially numbered, opaque, sealed envelopes), describing any steps to conceal the sequence until interventions are assigned	10
Implementation	16c	Who will generate the allocation sequence, who will enrol participants, and who will assign participants to interventions	10
Blinding (masking)	17a	Who will be blinded after assignment to interventions (eg, trial participants, care providers, outcome assessors, data analysts), and how	10
	17b	If blinded, circumstances under which unblinding is permissible, and procedure for revealing a participant's allocated intervention during the trial	na
**Methods: Data collection, management, and analysis**			
Data collection methods	18a	Plans for assessment and collection of outcome, baseline, and other trial data, including any related processes to promote data quality (eg, duplicate measurements, training of assessors) and a description of study instruments (eg, questionnaires, laboratory tests) along with their reliability and validity, if known. Reference to where data collection forms can be found, if not in the protocol	protocol
	18b	Plans to promote participant retention and complete follow-up, including list of any outcome data to be collected for participants who discontinue or deviate from intervention protocols	NA

For peer review only

Data management	19	Plans for data entry, coding, security, and storage, including any related processes to promote data quality (eg, double data entry; range checks for data values). Reference to where details of data management procedures can be found, if not in the protocol	10
Statistical methods	20a	Statistical methods for analysing primary and secondary outcomes. Reference to where other details of the statistical analysis plan can be found, if not in the protocol	11
	20b	Methods for any additional analyses (eg, subgroup and adjusted analyses)	11
	20c	Definition of analysis population relating to protocol non-adherence (eg, as randomised analysis), and any statistical methods to handle missing data (eg, multiple imputation)	11
**Methods: Monitoring**			
Data monitoring	21a	Composition of data monitoring committee (DMC); summary of its role and reporting structure; statement of whether it is independent from the sponsor and competing interests; and reference to where further details about its charter can be found, if not in the protocol. Alternatively, an explanation of why a DMC is not needed	14
	21b	Description of any interim analyses and stopping guidelines, including who will have access to these interim results and make the final decision to terminate the trial	Na
Harms	22	Plans for collecting, assessing, reporting, and managing solicited and spontaneously reported adverse events and other unintended effects of trial interventions or trial conduct	9,14
Auditing	23	Frequency and procedures for auditing trial conduct, if any, and whether the process will be independent from investigators and the sponsor	14
**Ethics and dissemination**			
Research ethics approval	24	Plans for seeking research ethics committee/institutional review board (REC/IRB) approval	15
Protocol amendments	25	Plans for communicating important protocol modifications (eg, changes to eligibility criteria, outcomes, analyses) to relevant parties (eg, investigators, REC/IRBs, trial participants, trial registries, journals, regulators)	
Consent or assent	26a	Who will obtain informed consent or assent from potential trial participants or authorised surrogates, and how (see Item 32)	8
	26b	Additional consent provisions for collection and use of participant data and biological specimens in ancillary studies, if applicable	na

For peer review only

Confidentiality	27	How personal information about potential and enrolled participants will be collected, shared, and maintained in order to protect confidentiality before, during, and after the trial	10-11
Declaration of interests	28	Financial and other competing interests for principal investigators for the overall trial and each study site	22
Access to data	29	Statement of who will have access to the final trial dataset, and disclosure of contractual agreements that limit such access for investigators	11
Ancillary and post-trial care	30	Provisions, if any, for ancillary and post-trial care, and for compensation to those who suffer harm from trial participation	Main protocol
Dissemination policy	31a	Plans for investigators and sponsor to communicate trial results to participants, healthcare professionals, the public, and other relevant groups (eg, via publication, reporting in results databases, or other data sharing arrangements), including any publication restrictions	15
	31b	Authorship eligibility guidelines and any intended use of professional writers	na
	31c	Plans, if any, for granting public access to the full protocol, participant-level dataset, and statistical code	na
**Appendices**			
Informed consent materials	32	Model consent form and other related documentation given to participants and authorised surrogates	Na
Biological specimens	33	Plans for collection, laboratory evaluation, and storage of biological specimens for genetic or molecular analysis in the current trial and for future use in ancillary studies, if applicable	na

*It is strongly recommended that this checklist be read in conjunction with the SPIRIT 2013 Explanation & Elaboration for important clarification on the items. Amendments to the protocol should be tracked and dated. The SPIRIT checklist is copyrighted by the SPIRIT Group under the Creative Commons "Attribution-NonCommercial-NoDerivs 3.0 Unported" license.

For peer review only - http://bmjopen.bmj.com/site/about/guidelines.xhtml

BMJ Open

Flash-glucose monitoring with the FreeStyle Libre 2 compared to self-monitoring of blood glucose in sub-optimally controlled type 1 diabetes: the FLASH-UK randomised controlled trial protocol

Journal:	*BMJ Open*
Manuscript ID	bmjopen-2021-050713.R1
Article Type:	Protocol
Date Submitted by the Author:	20-May-2021
Complete List of Authors:	Wilmot, Emma; University Hospitals of Derby and Burton NHS Foundation Trust; University of Nottingham Faculty of Medicine and Health Sciences<br>Evans, Mark; Cambridge University, Institute of Metabolic Science<br>Barnard-Kelly, Katharine; Bournemouth University<br>Burns, M; The University of Manchester<br>Cranston, Iain; Portsmouth Hospitals NHS Trust<br>Elliott, Rachel; Manchester Centre for Health Economics<br>Gkountouras, G; The University of Manchester<br>Kanumilli, N<br>Krishan, A; The University of Manchester<br>Kotonya, C; University Hospitals of Derby and Burton NHS Foundation Trust<br>Lumley, S<br>Narendran, P; University of Birmingham College of Medical and Dental Sciences<br>Neupane, Sankalpa; Norfolk and Norwich University Hospital NHS Trust, Elsie Bertram Diabetes Centre<br>Rayman, Gerry; Ipswich Hospital NHS Trust, The Ipswich Diabetes Centre and Research Unit<br>Sutton, Christopher; University of Manchester, Division of Population Health, Health Services Research & Primary Care<br>Taxiarchi, V P; The University of Manchester<br>Thabit, H; Manchester University NHS Foundation Trust; The University of Manchester<br>Leelarathna, L; Manchester University NHS Foundation Trust; The University of Manchester
**Primary Subject Heading**:	Diabetes and endocrinology
Secondary Subject Heading:	Diabetes and endocrinology
Keywords:	General diabetes < DIABETES & ENDOCRINOLOGY, HEALTH ECONOMICS, Clinical trials < THERAPEUTICS, Diabetes & endocrinology < INTERNAL MEDICINE

SCHOLARONE™
Manuscripts

I, the Submitting Author has the right to grant and does grant on behalf of all authors of the Work (as defined in the below author licence), an exclusive licence and/or a non-exclusive licence for contributions from authors who are: i) UK Crown employees; ii) where BMJ has agreed a CC-BY licence shall apply, and/or iii) in accordance with the terms applicable for US Federal Government officers or employees acting as part of their official duties; on a worldwide, perpetual, irrevocable, royalty-free basis to BMJ Publishing Group Ltd ("BMJ") its licensees and where the relevant Journal is co-owned by BMJ to the co-owners of the Journal, to publish the Work in this journal and any other BMJ products and to exploit all rights, as set out in our licence.

The Submitting Author accepts and understands that any supply made under these terms is made by BMJ to the Submitting Author unless you are acting as an employee on behalf of your employer or a postgraduate student of an affiliated institution which is paying any applicable article publishing charge ("APC") for Open Access articles. Where the Submitting Author wishes to make the Work available on an Open Access basis (and intends to pay the relevant APC), the terms of reuse of such Open Access shall be governed by a Creative Commons licence – details of these licences and which Creative Commons licence will apply to this Work are set out in our licence referred to above.

Other than as permitted in any relevant BMJ Author's Self Archiving Policies, I confirm this Work has not been accepted for publication elsewhere, is not being considered for publication elsewhere and does not duplicate material already published. I confirm all authors consent to publication of this Work and authorise the granting of this licence.

For peer review only

Flash-glucose monitoring with the FreeStyle Libre 2 compared to self-monitoring of blood glucose in sub-optimally controlled type 1 diabetes: the FLASH-UK randomised controlled trial protocol

Wilmot E G[1], Evans M L[2], Barnard-Kelly K[3], Burns M[4], Cranston I[5], Elliott R A[6] , Gkountouras G[6], Kanumilli N[7] Krishan A[6], Kotonya C[8], Lumley S[9] , Narendran P[10], Neupane S[11], Rayman G[12], Sutton C J[6], Taxiarchi V P[6], Thabit H[13, 14] , Leelarathna L[13, 14].

[1]University Hospitals of Derby and Burton NHS Foundation Trust, Royal Derby Hospital, Derby, UK.
[2]Wellcome Trust-MRC Institute of Metabolic Science, NIHR Cambridge Biomedical Research Centre, Cambridge University Hospitals and University of Cambridge, Cambridge, UK
[3]Barnard Health, BHR Limited, Hampshire, UK
[4]Manchester Clinical Trials Unit, University of Manchester, Manchester, UK
[5]Academic Department of Diabetes & Endocrinology, Queen Alexandra Hospital, Cosham, Portsmouth, UK
[6]Manchester Centre for Health Economics, Division of Population Health, Health Service Research & Primary Care, University of Manchester, Manchester, UK
[7]Northenden Group Practice, 489 Palatine Road, Northernden, Manchester, UK
[8]University Hospitals of Derby and Burton NHS Foundation Trust, Queen's Hospital Burton, Burton-on-Trent, UK
[9]The Adam Practice, Upton, Poole, Dorset, UK
[10]Institute of Immunology and Immunotherapy, College of Medical and Dental Sciences, University of Birmingham, Edgbaston, Birmingham, UK
[11] Elsie Bertram Diabtes Centre ,Norfolk and Norwich University Hospitals NHS Foundation Trust, Colney Lane, Norwich, UK
[12]Diabetes Research Office, East Suffolk and North Essex NHS Foundation Trust, Ipswich, UK
[13]Manchester Diabetes Centre, Manchester Royal Infirmary, Manchester University NHS Foundation Trust, Manchester Academic Health Science Centre, Manchester, UK
[14]Division of Diabetes, Endocrinology and Gastroentrology, Faculty of Biology, Medicine and Health, University of Manchester, Manchester, UK

Corresponding authors:

Dr Lalantha Leelarathna

Email: Lalantha.Leelarathna@mft.nhs.uk

And

Dr Emma Wilmot

Email: emma.g.wilmot@gmail.com

Word count: 4210 excluding Abstract, Box and Tables (max 4000)
Abstract: 300
Figures: 1, Boxes: 1, Tables 2

For peer review only - http://bmjopen.bmj.com/site/about/guidelines.xhtml

Abstract (max 300 words)

Introduction Optimising glycaemic control in Type 1 diabetes (T1D) remains challenging. Flash glucose monitoring with FreeStyle Libre 2 (FSL2) is a novel alternative to the current standard of care self-monitoring of blood glucose (SMBG). No randomised controlled trials to date have explored the potential benefits of FSL2 in T1D. We aim to assess the impact of FSL2 in people with sub-optimal glycaemic control T1D in comparison with SMBG.

Methods This open-label, multi-centre, randomised (via stochastic minimisation), parallel design study conducted at 8 UK secondary and primary care centres will aim to recruit 180 people age ≥16 years with T1D for >1 year and HbA1c 7.5%-11%. Eligible participants will be randomised to 24 weeks of FSL2 (intervention) or SMBG (control) periods, after two week of blinded sensor wear. Participants will be assessed virtually or in-person owing to the COVID-19 pandemic. HbA1c will be measured at baseline, 12 and 24 weeks (primary outcome). Participants will be contacted at 4 and 12 weeks for glucose optimisation. Control participants will wear a blinded sensor during the last 2 weeks. Psychosocial outcomes will be measured at baseline and 24 weeks. Secondary outcomes include sensor-based metrics, insulin doses, adverse events and self-report psychosocial measures. Utility, acceptability, expectations and experience of using FSL2 will be explored. Data on health service resource utilisation will be collected.

Analysis Efficacy analyses will follow intention-to-treat principle. Outcomes will be analysed using Analysis of Covariance, adjusted for the baseline value of the corresponding outcome, minimisation factors and other known prognostic factors. Both within-trial and life-time economic evaluations, informed by modelling from the perspective of the NHS setting, will be performed.

Ethics and trial registration The study was approved by Greater Manchester West Research Ethics Committee (reference 19/NW/0081). Informed consent will be sought from all participants. The study is registered at ClinicalTrials.gov (NCT03815006).

For peer review only

For peer review only - http://bmjopen.bmj.com/site/about/guidelines.xhtml

Trial registration number: ClinicalTrials.gov NCT03815006 (23/01/2019)

The study website: https://sites.manchester.ac.uk/flash/

Protocol version: 4.0 dated 29/06/2020

Trial Sponsor:

Manchester University NHS Foundation Trust
Division of Research & Innovation
Manchester Royal Infirmary,
Oxford Road
Manchester
M13 9WL

For peer review only

For peer review only - http://bmjopen.bmj.com/site/about/guidelines.xhtml

Strengths and limitations of this study

- Flash-UK is a multi-centre randomised controlled trial of the novel FreeStyle Libre 2 flash glucose monitor over a 6 month follow up period assessing the impact on people living with type 1 diabetes and sub-optimal glucose control, in comparison to self-monitoring of blood glucose, with a primary outcome of HbA1c.

- It is the first randomised study to assess the clinical efficacy & health economic benefits of the Libre 2 device providing high quality data for UK policy makers.

- The integration of a virtual assessment pathway into the trial design ensures eligible candidates are not restricted from participation owing to the COVID-19 pandemic.

- A wide range of secondary outcomes including continuous glucose monitoring data and psychosocial outcomes will provide detailed insight into the impact of this technology on people living with Type 1 diabetes.

- The study is open (unblinded) and conducted in UK National Health Service only. Some findings from the study may only be applicable to UK NHS setting.

For peer review only

Introduction

Type 1 diabetes mellitus (T1D) is one of the most common endocrine conditions. It is estimated that approximately 415 million adults (5-15% type 1 diabetes) and 520 thousand children (95% type 1 diabetes) worldwide suffer from diabetes (1). Despite the availability of therapeutic options such as self-monitoring of blood glucose (SMBG), structured education, rapid-acting insulin analogues and insulin pump therapy, glycaemic control in the majority of people with Type 1 diabetes remains suboptimal (2) and they therefore remain prone to complications associated with high glucose levels, such as kidney failure and blindness (3). In England less than one third of people with type 1 diabetes achieve a HbA1c level <7.5% (4). Studies have shown a strong relationship between the frequency of SMBG and HbA1c, with the National Institute of Clinical Excellence recommending 4-10 checks per day (5, 6). However due to pain, inconvenience and the limited information a moment-in-time glucose value provides, finger-stick glucose monitoring remains a key barrier in achieving near normal glucose levels.

In 2014, the FreeStyle Libre Flash Glucose Monitoring System (FSL) (Abbott Diabetes Care, Oxon, UK) became available as a potential alternative to SMBG. The sensor utilises wired enzyme technology (7) to continuously measure interstitial glucose levels. The arm-worn sensor is scanned using a reader or mobile phone app and provides information on current and previous glucose levels and trends. The IMPACT randomised controlled multicentre European trial was the largest study to evaluate the FSL (8) in 328 participants with well-controlled (HbA1c <= 7.5%, 59 mmol/mol) Type 1 diabetes, a third of whom used CSII therapy. FSL use was associated with improvement in a range of glucose-related outcomes: a 38% reduction in time spent in hypoglycaemia (<3.9 mmol/l). There was an increase in glucose time in range but HbA1c was unchanged. The impact of FSL was also assessed in those with Type 2 diabetes on intensive insulin therapy in a large multi-centre randomised European study of 224 participants (9). Time in hypoglycaemia (<3.9mmol/l) reduced compared with controls by 43% but HbA1c was unchanged. In both randomised controlled trials treatment satisfaction was higher in FSL users and no device-related serious adverse events were reported, suggesting that flash glucose monitoring also offers a safe replacement to SMBG in those with diabetes on intensive insulin therapy.

Subsequently a range of observational studies have demonstrated benefits of FSL use for HbA1c and hypoglycaemia (10-15). Campbell et al. evaluated the use of FSL as a replacement for SMBG in young people (4-17 years) (n=76, 58% CSII users, mean (SD) age 10.3(4.0) years, baseline HbA1c 7.9 (1.0)% (63 mmol/mol), T1D duration 5.4(3.7) years) with Type 1 diabetes in a single-arm European multi-centre trial (16). After 2 weeks' baseline masked wear, participants used FSL for 8 weeks. HbA1c

For peer review only - http://bmjopen.bmj.com/site/about/guidelines.xhtml

significantly improved vs. baseline, -0.4±0.6%, p<0.0001. However, a subsequent 6-month randomised controlled parallel-arm trial of the FSL in 64 participants aged 13-20 years with type 1 diabetes and glycated haemoglobin (HbA_{1c}) ≥9% (≥75 mmol/mol) demonstrated no statistically significant difference between groups for changes in HbA_{1c} at 6 months (adjusted mean -0.2% greater improvement for FSL [95% CI -0.9 to 0.5] [-2.1 mmol/mol (95% CI -9.6 to 5.4)]; p= 0.58) (17). The discrepancy in findings between the observational and randomised controlled trials in the paediatric population highlights the importance of high-quality randomised trials to investigate the benefits of this technology. To date, although some are planned, there has been no published randomised controlled trial to demonstrate the impact of the FSL in adults with Type 1 diabetes and high HbA1c levels (18,19).

More recently, in 2020, the Freestyle Libre 2 (FSL2) launched in Europe. This is very similar to FSL except for an optional additional alarm to alert users when the glucose level falls outside their target range, in addition to improved senor accuracy (20). The FSL2 was launched in the United Kingdom in January 2021. To the best of our knowledge no randomised controlled trials to assess the efficacy of the FSL2 system has been conducted or in progress. No economic evaluation assessing relative costs and benefits has been carried out in this patient group, nor has there been an assessment of patient acceptability. The purpose of the Flash UK study is to address this gap in the evidence and determine whether use of flash glucose monitoring with the FSL2 device will improve HbA1c over a 24-week randomised period compared to SMBG in adults with type 1 diabetes and sub-optimal glycaemic control.

Methods and analysis

Trial design

Flash-UK is an open, multi-centre, randomised (1:1), parallel-group superiority trial, in adults and adolescents (16 years and older) with type 1 diabetes and sub-optimal glycaemic control (HbA1c 7.5% to 11% (59 to 97 mmol/mol), either on continuous subcutaneous insulin infusion (CSII) or multiple daily injections (MDI), contrasting flash glucose monitoring using the FSL2 device with traditional finger-stick SMBG for 24 weeks. The study flow chart is outlined in Figure 1.

Study setting

Eight (one primary and seven secondary) care diabetes services from across England, UK.

1. Diabetes Centres within Manchester University Foundation Trust

For peer review only - http://bmjopen.bmj.com/site/about/guidelines.xhtml

2. Diabetes Centres within University Hospitals of Derby and Burton NHS Foundation Trust
3. University Hospitals Birmingham NHS Foundation Trust
4. Wolfson Adult Diabetes Endocrine Clinic, Cambridge Universities Hospitals, Cambridge
5. Norfolk and Norwich University Hospital, Norwich
6. Queen Alexandra Hospital, Portsmouth
7. Ipswich Hospital, East Suffolk and North Essex NHS Foundation Trust, Ipswich
8. Wareham Surgery (Wareham) and The Adam Practice (Poole), NHS England Primary Care GP Practices

For peer review only

Eligibility criteria

The major eligibility criteria are age ≥16 years with Type 1 diabetes for at least 1 year and a HbA1c between 7.5 and 11%. The full criteria are available in Box 1.

Interventions

FreeStyle Libre 2

This intervention is the CE marked FreeStyle Libre 2 (FSL2) flash glucose monitoring device (Abbott Diabetes Care, Oxon, UK). The FSL2 glucose sensor is an arm worn sensor intended to last for 14 days. The component not directly attached to the patient is the handheld reader and/or mobile phone app which display current and historical glucose data. Education and training about insertion and initiation of the sensor as well as how to use flash-glucose monitoring data for treatment optimisation is also provided. Encouragement is also provided to download data at home to identify pattern recognition. This session, designed to meet the needs of the individual, is conducted by a professional diabetes educator or a member of the study team.

Finger-stick SMBG

This is continuation of usual treatment. Additionally, encouragement will be given to use finger-stick glucose levels to optimise therapy and education about insulin dose adjustments using finger-stick glucose levels will also be provided. Participants in both arms will also receive training on sick day rules and dealing with hypo and hyperglycaemia as required.

Participants will be provided with an information leaflet following the training session, although the information provided will be tailored to the respective intervention. The leaflet provided in conjunction with the FSL2 will include sign-posting to educational videos provided by the Association

For peer review only - http://bmjopen.bmj.com/site/about/guidelines.xhtml

of British Clinical Diabetologists (ABCD) (https://abcd.care/dtn/education) and Bertie online (www.bertieonline.org.uk) in conjunction with the finger-stick SMBG.

Outcomes

The FLASH-UK study is designed to assess an extensive array of clinical, psychosocial and usability outcomes (Table 1). An outline of a dedicated health economic evaluation is provided below. The primary end point is HbA1c level at 24 weeks post-randomisation. Sensor based outcomes comparing the FSL2 with SMBG will be assessed for the last 2 weeks of the intervention period. Prespecified sensor based outcomes include time in the target range (3.9 to 10mmol/l), duration of hypoglycaemia < 3.5 mmol/l (63 mg/dl); < 3.0 mmol/l (54mg/dl); < 2.8 mmol/l (50 mg/dl), duration of hyperglycaemia (>10 mmol/l (180 mg/dL), and >16.7mmol/l (>300 mg/dL), and glucose variability (Standard deviation & coefficient of variation). All the sensor based metrics will also be analysed separately for daytime (7:00-23:00 hours) and night-time (23:00-7:00 hours) in addition to 24 hour period. Insulin usage data will be compared between the two arms. Harms outcomes include the frequency of severe hypoglycaemic episodes as defined by American Diabetes Association, Frequency of significant ketosis events (plasma ketones >3mmol/l) and nature and severity of other adverse events. Information on any other anti-diabetes therapy will be collected.

Questionnaires (Table 1) will be employed at baseline and the end of the study to evaluate participants' responses in terms of quality of life, diabetes distress, needle burden, disordered eating, depression and diabetes treatment satisfaction using EQ-5DL-5L questionnaire, Type 1 Diabetes Distress Scale (DDS), Diabetes fear of injecting and self-testing (D-FISQ) questionnaire, Diabetes Eating Problem Survey (DEPS-R), Diabetes Treatment Satisfaction Questionnaire (DTSQ), Patient Health Questionnaire (PHQ-9) and The Glucose Monitoring Satisfaction Survey (GMSS). Hypoglycaemia burden will be assessed using Clarke questionnaire and Gold score.

Participant timeline & data collection

The study will consist of 6 visits for those in the FSL2 arm and 7 visits in the SMBG arm. Visits 1 and 2 can be conjoined. The study flow chart is shown in Figure 1. Key activities undertaken during each study visit are shown in Table 2.

Due to the COVID-19 pandemic all study visits can be conducted either face to face or virtually (supported by telephone and videoconferencing as appropriate) as indicated in the approved study advertisement and participant information sheet. At visit 1, following informed written consent (model consent form and patient information sheet is included in the supplementary file) by trained members of the research team, medical and diabetes history will be recorded including presence of

For peer review only - http://bmjopen.bmj.com/site/about/guidelines.xhtml

diabetes complications, hypoglycaemia burden including the use of Clarke and Gold questionnaires, use of concomitant diabetes medications, ethnicity, body weight and height measurement; demographic data, insulin therapy, occupation and educational attainment, any history of disordered eating or needle phobia, previous participation in structured education, carbohydrate counting status, use of bolus calculator and patient self-report psychosocial questionnaires will be completed. Blood samples will be taken to measure HbA1c.

At visit 2 the FreeStyle Libre Pro blinded CGM device will be inserted, to be worn for 2 weeks. For participants on the virtual pathway, participant will be taught to self insert the sensor. At Visit 3, participant adherence / tolerance of using the flash-CGM over the preceding 14 days will be assessed. To proceed to randomisation at least 10 of 14 days blinded CGM data must be available. Those who have <10 days data will be provided with a new FreeStyle Libre pro sensor and reader to obtain the minimum data requirements prior to randomisation or discontinue from trial participation if there is a continued adverse reaction to the sensor adhesive.

Participants will be followed up at 4, 12 and 24 weeks post randomisation (+/- 2 weeks). At these visits glucose data will be downloaded and participant diaries which collect information on insulin doses and carbohydrate intake in last 5 days will be collected. Participants in the virtual pathway will be encouraged to upload data from home. Blood tests for HbA1c will also be performed at 12 and 24 weeks after randomisation. Those in the SMBG group will attend an additional visit at 22 weeks to have the blinded CGM sensor fitted to be worn from 22-24 weeks. At 24 weeks, body weight measurement will be made where possible and the participant will be asked to complete questionnaires. In addition, participants in the FSL2 arm will be asked to complete a questionnaire exploring expectations and experience of using FSL2 during the study. Healthcare resource use in primary and secondary healthcare settings will also be collected at 24 weeks.

Adverse events will be routinely collated at study visits. The independent data monitoring committee (IDMC) will be informed of all serious adverse events and any unanticipated adverse device effects that occur during the study and will review compiled adverse event data at periodic intervals.

Sample size

The target effect size (minimally clinically important HbA1c difference) of 0.4% was chosen as this is consistent with other relevant trials (0.5% in REPOSE (21), 0.4% in DIAMOND (22), 0.3% in GOLD (23)). To achieve 90% power using an independent-sample t-test for 24-week HbA1c values (2-tail alpha=0.05, power=0.80, effect size=0.4%, SD=0.8% (23) i.e. Standardised Effect Size = 0.5), 128

For peer review only - http://bmjopen.bmj.com/site/about/guidelines.xhtml

participants with primary outcome data are required. This is inflated to a target of 150 randomised (assuming maximum 15% attrition) and up to 180 recruited (to allow for pre-randomisation losses). The use of ANCOVA with adjustment for baseline values of HbA1c and other minimisation factors should increase power.

Recruitment

Participants will be identified by treating clinicians in each centre and participant information sheet provided. The first study site was activated on the 20/12/2019 and first participant was recruited on the 09/01/2020. Each site will each aim to recruit between 14 and 30 participants up to a total of 180 participants. The study has been advertised via social media and through the official trial website (https://sites.manchester.ac.uk/flash/), providing those who do not usually attend study centres to have the opportunity to participate. As of 31/01/2021, 109 participants had been randomised. It is anticipated that recruitment will be completed by 30/04/2021. In the event that a participant meets withdrawal criteria, sites will engage with participants to seek their verbal agreement to volunteer their continued participation for HbA1c time-points (post randomisation) and exit questionnaires under the intention to treat analysis principle.

Allocation

Sequence generation

Allocation to one of the two intervention arms (24-weeks use of flash-glucose monitoring or 24 weeks use of conventional finger-stick SMBG) will use stochastic minimisation (factors: study centre, baseline HbA1c (7.5%-9.0%; >9.0%-11%), treatment modality (MDI;CSII), prior participation in structured education course (yes; no) and current use of bolus calculator (yes; no)).

Allocation concealment mechanism and its implementation

Allocation will be implemented using the web-based Sealed Envelope™ software (Sealed Envelope Ltd, London, UK) randomisation system. This will be independently managed by Manchester CTU (MCTU) staff. Participants will have their pseudonymised registration details entered into the randomisation system by delegated recruitment site research teams who will also confirm participant eligibility; only then will the system generate an email confirmation with their allocation.

Blinding (masking)

The trial is fully open. It is not possible to blind investigators or participants to the delivery or receipt of the intervention; data collectors and statisticians are also unblinded to treatment allocations.

For peer review only - http://bmjopen.bmj.com/site/about/guidelines.xhtml

Laboratory analysis

Blood samples for the measurement of HbA1c levels will be taken at three different time points: screening, 12 and 24 weeks. This can be completed using the local laboratory (face to face clinic) or at home where the participant will collect a capillary blood sample using a validated home self-test kit (TDL TINY™) and send in a pre-paid envelope to The Doctors Laboratory Ltd (London, UK).

Data management

Confidentiality of participant data shall be observed at all times during the study. Personal details for each participant taking part in the research study and linking them to a unique identification number will be held locally on a study screening log in the Investigator Site File at each of the investigation centres. The study identification number will be used on the case report forms,on all the blood samples that are collected throughout the study and FreeStyle Libre 2 and FreeStyle Libre Pro data submitted to study specific Libreview database (Libreview.com). Names and full addresses will not be used. Electronic data will be stored on password-protected computers. Only members of the research team and collaborating institutions will have password access to the anonymised electronic data. Paper copies of the data will be stored for 15 years. Direct access to the source data will be provided for monitoring, audits, REC review during and after the study. The fully anonymised data may be shared with third parties (EU or non-EU based) for the purposes of advancing management and treatment of diabetes. Standard procedures agreed by the MCTU, Chief Investigator and Clinical Principal Investigators are in place for data review, database cleaning and issuing and resolving data queries.

Analysis

Statistical Methods

All efficacy and safety analyses will be conducted following the intention to treat (ITT) principle in which all randomised participants are analysed in their allocated treatment group whether or not they receive their randomised treatment. All baseline, 12-week and 24-week outcome data will be presented descriptively, both overall and within treatment group, using mean (SD), median (IQR) or frequency (percentage), as appropriate. All statistical tests will use a 2-sided significance level of 5% (unless otherwise specified). All confidence intervals will be presented at a level of 95% and will be two sided. All statistical analyses will be performed using Stata (StataCorp, College Station TX, USA).

For peer review only - http://bmjopen.bmj.com/site/about/guidelines.xhtml

The primary outcome analysis will evaluate between group differences in HbA1c levels at the end of the 24-week treatment period. An analysis of covariance (ANCOVA) model will be used, with 24-week HbA1c as the outcome and trial arm effect as the focus, and with adjustment for baseline HbA1c, and the other baseline variables included in the minimisation allocation algorithm as covariates. If more than 10% HbA1c data are missing at 24 weeks (or a >10% difference between missing data percentages in the two arms) multiple imputation will be used in order to implement a more complete ITT analysis of the substantive ANCOVA model (otherwise this will be performed as a sensitivity analysis, with a complete-case analysis used as the primary analysis). The imputation model will include baseline and 12-week HbA1c, all the baseline variables used in the outcome model and any other recorded variables found to be predictive of missingness in the 24-week outcome in exploratory analyses (via a logistic regression model, with terms included using a 10% significance level). Sensitivity analyses will be performed to (i) examine robustness of how missing data are handled, (ii) examine efficacy of treatment through Complier-Adjusted Causal Effect modelling, (iii) examine impact of data collected outside the visit window, and (iv) examine potential impact of Covid-19 on the primary outcome.

Quantitative secondary outcomes will also be analysed using ANCOVA and binary secondary outcomes will be analysed using logistic regression. In each case, the analysis will be adjusted for the baseline value of the outcome and the adjustment factors used in the model for HbA1C. Harms data will be reported descriptively as frequencies and percentages (%), both overall and by intervention arm.

Subgroup analysis will be performed by the addition of interactions between intervention arm and the subgrouping factor. All subgroup analyses will be performed separately. Planned subgroups are: baseline HbA1c; treatment modality; prior participation in structured education course; age group; education level; hypoglycaemia unawareness; deprivation index quintile; sex; ethnic group.

Further sensitivity and/or subgroup analyses will be performed as appropriate. These together with fuller details of the analyses proposed above, and any additional analyses, will be included in a full Statistical Analysis Plan that will be approved by the Trial Steering Committee (TSC) prior to any analysis of the outcome data.

Economic analysis

The economic evaluation will determine the difference in costs and outcomes generated by the FSL2 device compared with SMBG. The economic evaluation will be conducted from the perspective of NHS/Personal Social Services (PSS) following standard quality design and reporting criteria (24).

For peer review only - http://bmjopen.bmj.com/site/about/guidelines.xhtml

A within-trial cost-utility analysis will compare differences in total costs and differences in quality of life using QALYs derived from the EQ-5D-5L. QALYs will be calculated by attaching available utility weights to the health states generated from the EQ-5D-5L, using area under the curve methods with an assumption of a linear change between time points, controlling for baseline. Person-level costs will be generated for each person in the FSL2 device and SMBG arms from a combination of trial-based resource use with published unit costs, allowing comparison in terms of costs to NHS and PSS (25). Costs will be compared between the two groups using non-parametric methods.

Modelling the potential effect of the intervention on costs and outcomes beyond the trial period will provide a better idea of overall impact as the benefits of controlling HbA1c are likely to be seen after the endpoint of the trial. Therefore, we will carry out an economic evaluation informed by modelling to estimate longer-term benefits and costs, from an NHS/PSS perspective.

A commercially available cost-effectiveness model, the IMS Centre for Outcomes Research and Effectiveness diabetes model version 8.5 (IMS Health, Danbury, CT, USA), will be used for this economic evaluation. The model consists of 15 submodels designed to simulate diabetes-related complications, non-specific mortality and costs over time. It also incorporates the costs and effects of hypoglycaemia, so is particularly well-suited to this study. Two major validation papers on the IMS CDM have been published to date. (26,27) The IMS Core Diabetes Model has also been used in a UK-based recent health technology assessment of CGM commissioned by NICE.(28)

Incremental cost-effectiveness ratios (ICERs) will be calculated in the event of the intervention having either higher costs and better outcomes or lower costs and worse outcomes (no scenarios of dominance, based on QALYs and trial primary outcome). The base case analysis will estimate the mean costs and QALYs across the treatment arms. The overall impact of uncertainty will be assessed by generating cost-effectiveness planes from bootstrapped resamples, and distributional assumptions about the transition probabilities, costs and utility values will be made. Cost-effectiveness acceptability curves (CEACs) will be constructed to show the probability that the intervention is cost-effective for different willingness to pay (WTP) thresholds. Incremental economic analysis using the IMS CORE model will require the model's time horizon to be set to 80 years, which approximates to a lifetime horizon. All costs and effects will be discounted by 3.5%, as per NICE guidance(25,29).

Process evaluation

The process evaluation will be undertaken to explain discrepancies between expected and observed outcomes, to understand how context influences outcomes, and to provide insights to aid

For peer review only - http://bmjopen.bmj.com/site/about/guidelines.xhtml

implementation. Specifically, we will investigate whether treatment is consistent with the behaviour change theories, which underpin it and contextual factors have affected implementation. Process evaluation will use a pipeline logic model, showing causal links between resources, activities and outcomes, integrating the National Institute for Health Behaviour Change Consortium's (NIHBCC's) approach to treatment fidelity (30) and a modified version of Linnan and Steckler's framework for process evaluation.(31) We will describe context qualitatively and take a mixed methods approach to characterising recruitment, reach, dose delivered/received and fidelity, with triangulation between data sources.(32) Free-text response questionnaires will be completed by intervention designers, health professionals and trial participants, and analyses combined with trial data, including FSL2 device utilization and SMBG data which will be analysed descriptively (including the use of appropriate graphical representation), within arms where appropriate, will be synthesised and findings triangulated appropriately.

Study Management

Trial Management Group (TMG)

TMG comprising the Chief Investigator, Co-Investigators, Trial Managers, Trial Statistician, Trial Health Economist, Monitor, and Data Manager will meet quarterly to discuss the operational aspects of the study.

Trial Steering Committee

TSC with an independent chair has been appointed. Independent membership of the TSC includes two clinical members (including the chair), two service users, health economist and statistician; non-independent members are Chief investigator (CI) and Statistician Co-Investigator (only Statistician voting). Other members of the study team including the Sponsor are invited to TSC meetings as observers only.

Independent Data Monitoring Committee

An IDMC comprising a clinical chair, another clinical expert and a statistician has been appointed. The IDMC will be informed of all serious adverse events and any unanticipated adverse device effects/events that occur during the study. The IDMC will review compiled adverse event data at periodic intervals. The IDMC will report to the TSC any safety concerns and recommendations for suspension or early termination of the investigation.

Study Monitoring

For peer review only - http://bmjopen.bmj.com/site/about/guidelines.xhtml

A detailed risk assessment was completed by MCTU and approved by the Sponsor. The procedures, source data transfer modalities and anticipated frequency for monitoring are documented in the Monitoring Plan. Copies of the risk assessment and the Monitoring Plan will be stored in the Trial Master File. The MCTU study monitor will be fully independent of both the Sponsor and Site Principal Investigators.

Authorised representatives of Sponsor, regulatory authority, or an Ethics Committee may perform audits or inspections at the recruiting centres, including source data verification.

Any substantial / non-substantial amendments will be managed by the clinical trials units following an approved quality standard operating procedure. Favourable outcomes will be conveyed to participating sites for implementation following local R&D.

Patient and public involvement

During the grant application process the study protocol received input from several patient groups from Manchester, Derby and Cambridge as well as the INPUT patient advocacy group. One of the study investigators also has Type 1 diabetes as do two members of the Trial Steering Committee.

Ethics and dissemination

The study will be conducted in accordance with the Declaration of Helsinki Ethical Principles for Medical Research involving Human Subjects (October 2000). It was approved by Greater Manchester West Research Ethics Committee on 21/03/2019REC reference 19/NW/0081. All participants will be provided with oral and written information about the trial, including the most common AEs, and the procedures involved in the study before obtaining written informed consent. The study results will be communicated to trial participants and disseminated in peer-review publications and through conference presentations. The data sharing plan is available in the supplementary file.

Conclusion

Flash UK is a multi-centre randomised controlled trial to assess the clinical efficacy of the FreeStyle Libre 2 system compared to SMBG in adults with suboptimal glucose control. The primary outcome is HbA1c with a wide range of secondary outcomes including glucometrcs, psychosocial and health economic outcomes. These results will inform and direct future clinical practice.

For peer review only - http://bmjopen.bmj.com/site/about/guidelines.xhtml

Box 1. Key Inclusion and Exclusion Criteria

**Inclusion Criteria**
1. The participant is ≥16 years old<br>2. The participant has type 1 diabetes, as defined by WHO for at least 1 year or is confirmed C-peptide negative if duration of diabetes is < 1 years<br>3. Participant is treated with insulin pump or multiple daily injection for at least 12 weeks and no plans to change treatment modality during next 28 weeks<br>4. The participant is literate in English for safe study conduct<br>5. Screening HbA1c ≥ 7.5% (58.5mmol/mol) and ≤ 11% (97 mmol/mol) based on analysis from local, central or third party external laboratory<br>6. The participant is willing to wear study glucose sensor and scan for glucose levels at regular intervals,<br>7. The participant is willing to follow study specific instructions and improve glucose control<br>8. Female participants of child bearing age should be on effective contraception or not sexually active / no plans for pregnancy
**Exclusion Criteria**
1. Non-type 1 diabetes mellitus including those secondary to chronic disease<br>2. Any other physical disease or people with known severe mental illness (psychotic disorder, bipolar disorder, dementia, substance and alcohol dependence, learning disabilities, depression with active suicidal ideation) which are likely to interfere with the normal conduct of the study and interpretation of the study results as judged by the investigator<br>3. Current users of real-time glucose monitoring sensors or flash-glucose monitoring for more than 4 weeks within last 12 weeks<br>4. Initiation of medications/treatments known to interfere with glucose metabolism (e.g: metformin, SGLT2 inhibitors, GLP-1 agonists, Pramlinatide) within the last 6 weeks or planning to start these medications within the next 6 months (patients on stable treatment is not an exclusion) or current or planned glucocorticoid use other than inhaled/ topical use<br>5. Known or suspected allergy against insulin<br>6. Severe visual impairment<br>7. Complete loss of hypoglycaemia awareness<br>8. Patient receiving dialysis / pre-dialysis based on history<br>9. More than one episode of severe hypoglycaemia as defined by American Diabetes Association (30) in preceding 24 weeks<br>10. Pregnancy, Planned Pregnancy in the next 8 months or breast feeding

For peer review only

Table 1: Secondary Outcomes

Category	Outcomes	Assessment Time-point
**HbA1c based**	HbA1c<br>HbA1c ≤ 53 mmol/mol (7.0%) [yes/no];<br><br>HbA1c ≤ 59 mmol/mol (7.5%) [yes/no];<br>Reduction in HbA1c ≥5.5 mmol/mol (0.5%) from baseline [yes/no];<br>Reduction in HbA1c ≥ 11 mmol/mol (1.0%) from baseline [yes/no];	Baseline and 12 weeks<br>Baseline, 12 weeks and 24 weeks<br>Baseline, 12 weeks and 24 weeks<br>Baseline, 12 weeks and 24 weeks<br>Baseline, 12 weeks and 24 weeks
**Sensor based (glucose)**	Time spent in the target glucose range 3.9 to 10.0 mmol/l (70 to 180mg/dl),<br>Time spent below target glucose (<3.9mmol/l) (<70mg/dl), < 3.5 mmol/l (63 mg/dl); < 3.0 mmol/l (54mg/dl); < 2.8 mmol/l (50 mg/dl)<br>Time spent above target glucose (10.0 mmol/l) (180 mg/dl), > 16.7 mmol/l) (300mg/dl).<br>Average glucose , Standard deviation, Coefficient of variation<br>AUC of glucose below 3.0mmol/l (54mg/dl)	Baseline and 24 weeks
**Non-sensor based (clinical)**	Daily average total insulin dose<br>Daily average basal insulin dose<br>Daily average bolus dose<br>Average number of boluses of rapid acting insulin per day<br>Frequency of severe hypoglycaemic episodes as defined by American Diabetes Association<br>Frequency of significant ketosis events (plasma ketones >3mmol/l)<br>Nature and severity of other adverse events	Baseline, 12 weeks and 24 weeks
**Psychosocial**	Type 1 Diabetes Distress Scale (T1-DDS)<br>Quality of Life (EQ-5D-5L)<br>Patient Health Questionnaire (PHQ-9)<br>Diabetes fear of injecting and self-testing questionnaire (D-FISQ)<br>The revised Diabetes Eating Problem Survey (DEPS-R)	Baseline and 24 weeks
**Process evaluation (utility and acceptability)**	FSL2 device utilization data, including: average number of scans per day (7:00-23:00 hours), per night (23:00-7:00 hours) and over the full 24-hour period; average number of days of usage per week for the full 24 week intervention<br><br>Number of finger-stick glucose level tests per day<br><br>Diabetes Treatment Satisfaction Questionnaire (DTSQ)<br>Glucose Monitoring Satisfaction Survey (GMSS)	Continuous (FSL arm only)<br><br><br>Continuous<br><br>Baseline and 24 weeks

For peer review only

For peer review only - http://bmjopen.bmj.com/site/about/guidelines.xhtml

Table 2: Schedule of study visits:

**Visit/ contact**	**Description**	**Time since randomisation**	**Start relative to previous / next Visit / Activity****
Visit 1	Recruitment & Screening visit: Consent<br>HbA1c, baseline bloods, baseline questionnaires	-2 to -3 weeks	-
Visit 2	Blinded flash glucose monitor insertion	-2 weeks	Within 1 to 2 weeks of Visit 1. Can coincide with Visit 1
Visit 3	Adherence assessment &<br>Randomisation<br>FSL2 / Self- monitoring of glucose initiation<br>- Education	0 weeks	After 2 weeks of Visit 2
Visit 4	Review data /optimisation<br>Collect participant diary	+4 weeks	After 4 weeks of Visit 3
Visit 5	Review data /optimisation. Data download<br>- HbA1c<br>- Collect participant diary	+12 weeks	After 8 weeks of Visit 5
Visit 6	Blinded flash glucose monitor insertion (Extra visit in SMBG arm)	+22 weeks	After 10 weeks of Visit 5
Visit 7	End of self-monitoring intervention arm<br>- HbA1c.<br>- Questionnaires<br>- Collect participant diary	+24 weeks	2 weeks after Visit 6

For peer review only - http://bmjopen.bmj.com/site/about/guidelines.xhtml

Authors' contributions

LL, EGW and ME conceptualised the study. LL, EGW, ME, IC, PN, SN, RE, CS, HT contributed to the grant application. MB is the Lead Clinical Trial Manager. CS, AK and VT are responsible for statistical analysis. All authors contributed to protocol development at various stages. LL, EGW, ME, IC, PN, SN, HT, GR, SL, NK, CK provides site oversight and are responsible for study conduct at each site. RE & GG are responsible for the health economic analysis. KB is responsible for the process evaluation. EGW & LL wrote the first draft of the manuscript and all authors reviewed and had the opportunity to comment on the content prior to submission. The corresponding author confirms that all co-authors are ICMJE recommendation compliant for the submission of this manuscript. No professional writers have been engaged for the preparation of this manuscript.

Competing interests statement

EGW has received personal fees from Abbott Diabetes Care, Dexcom, Eli Lilly, Insulet, Medtronic, Novo Nordisk, Sanofi Diabetes Care. LL has received personal fees from Abbott Diabetes Care, Dexcom, Insulet, Medtronic, Novo Nordisk, Sanofi Diabetes Care. ME has received personal fees from Abbott Diabetes Care, Eli Lilly, Medtronic, Novo Nordisk, Astra Zeneca, Zucara.SN has received personalised fees from QUIN,Roche. NK has received personal fees from Abbott,Eli Lilly,Novo Nordisk, Astra Zeneca, Napp, Sanofi. PN has acted as a clinical expert for NICE Medtech innovation briefing MIB110 relating to FreeStyle Libre system. GR has received lecture and consultancy fees from Abbott Diabetes UK. MB, KBK, GG, CS, CK, VT, AK, SN, RAE-No competing interests.

Funding statement

This work was supported by Diabetes UK grant number 18/0005836. The device manufacturer played no part in design, conduct or any other aspects of the study. The study devices were paid by the National Health Service (NHS) UK. Work was supported by the NIHR Cambridge Biomedical Research Centre. The University of Cambridge has received salary support for MLE from the National Health Service in the East of England through the Clinical Academic Reserve.

For peer review only

For peer review only - http://bmjopen.bmj.com/site/about/guidelines.xhtml

References

1. International Diabetes Federation, The Diabetes Atlas, Seventh Edition. Brussels:2015. http://www.diabetesatlas.org/, 2015
2. McKnight JA, Wild SH, Lamb MJ, Cooper MN, Jones TW, Davis EA, Hofer S, Fritsch M, Schober E, Svensson J, Almdal T, Young R, Warner JT, Delemer B, Souchon PF, Holl RW, Karges W, Kieninger DM, Tigas S, Bargiota A, Sampanis C, Cherubini V, Gesuita R, Strele I, Pildava S, Coppell KJ, Magee G, Cooper JG, Dinneen SF, Eeg-Olofsson K, Svensson AM, Gudbjornsdottir S, Veeze H, Aanstoot HJ, Khalangot M, Tamborlane WV, Miller KM: Glycaemic control of Type 1 diabetes in clinical practice early in the 21st century: an international comparison. Diabet Med 2015;32:1036-1050
3. Nathan DM, Cleary PA, Backlund JY, Genuth SM, Lachin JM, Orchard TJ, Raskin P, Zinman B, Diabetes Control and Complications Trial / Epidemiology of Diabetes Interventions and Complications (DCCT/EDIC) Study Research Group: Intensive diabetes treatment and cardiovascular disease in patients with type 1 diabetes. N Engl J Med 2005;353:2643-2653
4. NHS Digital: National Diabetes Audit - 2015-2016: Report 1, Care Processes and Treatment Targets. http://digitalnhsuk/catalogue/PUB23241 2017;
5. Miller KM, Beck RW, Bergenstal RM, Goland RS, Haller MJ, McGill JB, Rodriguez H, Simmons JH, Hirsch IB, Network TDEC: Evidence of a Strong Association Between Frequency of Self-Monitoring of Blood Glucose and Hemoglobin A1c Levels in T1D Exchange Clinic Registry Participants. Diabetes Care 2013;36:2009-2014
6. NICE guidance NG17. Type 1 diabetes in adults: diagnosis and management https://www.nice.org.uk/guidance/ng17 lst accessed 24/2/21
7. Danne T, de Valk HW, Kracht T, Walte K, Geldmacher R, Sölter L, von dem Berge W, Welsh ZK, Bugler JR, Lange K, Kordonouri O: Reducing glycaemic variability in type 1 diabetes self-management with a continuous glucose monitoring system based on wired enzyme technology. Diabetologia 2009;52:1496-1503 *FLASH-UK Protocol Version 4.0 29th June 2020: IRAS No.: 257593 Page 110 of 111*
8. Bolinder J, Antuna R, Geelhoed-Duijvestijn P, Kroger J, Weitgasser R: Novel glucose-sensing technology and hypoglycaemia in type 1 diabetes: a multicentre, non-masked, randomised controlled trial. Lancet 2016;388:2254-2263
9. Haak T, Hanaire H, Ajjan R, Hermanns N, Riveline JP, Rayman G: Use of Flash Glucose-Sensing Technology for 12 months as a Replacement for Blood Glucose Monitoring in Insulin-treated Type 2 Diabetes. Diabetes therapy : research, treatment and education of diabetes and related disorders 2017;8:573-586
10. Dover AR, Stimson RH, Zammitt NN, Gibb FW: Flash Glucose Monitoring Improves Outcomes in a Type 1 Diabetes Clinic. J Diabetes Sci Technol 2017;11:442-443
11. McKnight JA, Gibb FW: Flash Glucose Monitoring is associated with improved glycaemic control but use is largely limited to more affluent people in a UK diabetes centre. Diabet Med 2017;34:732
12. Ish-Shalom M, Wainstein J, Raz I, Mosenzon O: Improvement in Glucose Control in Difficult-to-Control Patients With Diabetes Using a Novel Flash Glucose Monitoring Device. J Diabetes Sci Technol 2016;10:1412-1413
13. Leelarathna L, Wilmot EG. Flash forward: a review of flash glucose monitoring. Diabet Med. 2018 Apr;35(4):472-482. doi: 10.1111/dme.13584. Epub 2018 Feb 27.
14. Deshmukh H, Wilmot EG, Gregory R, Barnes D, Narendran P, Saunders S, Furlong N, Kamaruddin S, Banatwalla R, Herring R, Kilvert A, Patmore J, Walton C, Ryder REJ, Sathyapalan T. Effect of Flash Glucose Monitoring on Glycemic Control, Hypoglycemia, Diabetes-Related Distress, and Resource Utilization in the Association of British Clinical Diabetologists (ABCD) Nationwide Audit. Diabetes Care. 2020 Sep;43(9):2153-2160.
15. Rose L, Klausmann G, Seibold A. Improving HbA1c Control in Type 1 or Type 2 Diabetes Using Flash Glucose Monitoring: A Retrospective Observational Analysis in Two German Centres. Diabetes Ther. 2020 Dec 11. doi: 10.1007/s13300-020-00978-9.
16. Campbell F, Kordonouri O, Murphy N, Stewart C: FreeStyle Libre Use for Self-Management of Diabetes in Children and Adolescents. Diabetes 2017;66 Suppl. 1A:LB28-LB28
17. Boucher SE, Gray AR, Wiltshire EJ, de Bock MI, Galland BC, Tomlinson PA, Rayns JA, MacKenzie KE, Chan H, Rose S, Wheeler BJ. Effect of 6 Months of Flash Glucose Monitoring in Youth With Type 1 Diabetes and High-Risk Glycemic Control: A Randomized Controlled Trial. Diabetes Care. 2020 Oct;43(10):2388-2395. doi: 10.2337/dc20-0613.
18. Secher AL, Pedersen-Bjergaard U, Svendsen OL, Gade-Rasmussen B, Almdal TP, Dørflinger L, Vistisen D, Nørgaard K. Study protocol for optimising glycaemic control in type 1 diabetes treated with multiple daily

For peer review only - http://bmjopen.bmj.com/site/about/guidelines.xhtml

insulin injections: intermittently scanned continuous glucose monitoring, carbohydrate counting with automated bolus calculation, or both? A randomised controlled trial.
BMJ Open. 2020 Apr 27;10(4):e036474.
19. Zhou Y, Deng H, Liu H, et al. Effects of novel flash glucose monitoring system on glycaemic control in adult patients with type 1 diabetes mellitus: protocol of a multicentre randomised controlled trialBMJ Open 2020;10:e039400. doi: 10.1136/bmjopen-2020-039400
20. Alva S, Bailey T, Brazg R, Budiman ES, Castorino K, Christiansen MP, Forlenza G, Kipnes M, Liljenquist DR, Liu H. Accuracy of a 14-Day Factory-Calibrated Continuous Glucose Monitoring System With Advanced Algorithm in Pediatric and Adult Population With Diabetes. J Diabetes Sci Technol. 2020 Sep 19:1932296820958754. doi: 10.1177/1932296820958754. Online ahead of print.#
21. REPOSE Study Group: Relative effectiveness of insulin pump treatment over multiple daily injections and structured education during flexible intensive insulin treatment for type 1 diabetes: cluster randomised trial (REPOSE). BMJ. 2017 Mar 30;356:j1285. doi: 10.1136/bmj.j1285.
22. Beck RW, Riddlesworth T, Ruedy K, Ahmann A, Bergenstal R, Haller S, Kollman C, Kruger D, McGill JB, Polonsky W, Toschi E, Wolpert H, Price D, Group DS: Effect of Continuous Glucose Monitoring on Glycemic Control in Adults With Type 1 Diabetes Using Insulin Injections: The DIAMOND Randomized Clinical Trial. JAMA 2017;317:371-378
23. Lind M, Polonsky W, Hirsch IB, Heise T, Bolinder J, Dahlqvist S, Schwarz E, Olafsdottir AF, Frid A, Wedel H, Ahlen E, Nystrom T, Hellman J: Continuous Glucose Monitoring vs Conventional Therapy for Glycemic Control in Adults With Type 1 Diabetes Treated With Multiple Daily Insulin Injections: The GOLD Randomized Clinical Trial. JAMA 2017;317:379-387
24. Husereau D, Drummond M, Petrou S, Carswell C, Moher D, Greenberg D, Augustovski F, Briggs AH,Mauskopf J, Loder E: Consolidated Health Economic Evaluation Reporting Standards (CHEERS)—Explanation and Elaboration: A Report of the ISPOR Health Economic Evaluation Publication Guidelines Good Reporting. Practices Task Force. Value in Health 2013;16:231-250
25. HM Treasury. The green book: appraisal and evaluation in central government. 2003. TSO: London. 2014.
26. Palmer AJ, Roze S, Valentine WJ, Minshall ME, Foos V, Lurati FM, Lammert M, Spinas GA: Validation of theCORE Diabetes Model against epidemiological and clinical studies. Curr Med Res Opin 2004;20 Suppl 1:S27-40
27. McEwan P, Foos V, Palmer JL, Lamotte M, Lloyd A, Grant D: Validation of the IMS CORE Diabetes Model.Value in Health 17:714-724
28. Riemsma R, Corro Ramos I, Birnie R, Büyükkaramikli N, Armstrong N, Ryder S, Duffy S, Worthy G, Al M, Severens J, Kleijnen J: Integrated sensor-augmented pump therapy systems [the MiniMed® Paradigm™ Veo system and the Vibe™ and G4® PLATINUM CGM (continuous glucose monitoring) system] for managing blood glucose levels in type 1 diabetes: a systematic review and economic evaluation. Health Technology Assessment (Winchester, England) 2016;20:1-252
29. National Institute for Health and Care Excellence. Guide to the Methods of Technology Appraisal. National Institute for Clinical Excellence; 2013.
30. Bellg AJ, Borrelli B, Resnick B, Hecht J, Minicucci DS, Ory M, et al. Enhancing treatment fidelity in health behavior change studies: best practices and recommendations from the NIH Behavior Change Consortium. Health Psychol [Internet]. 2004 [cited 2012 Mar 19];23(5):443–51
31. Linnan L, Steckler A. Process evaluation for public health interventions and research: an overview. In: Linnan L, Steckler A, editors. Process evaluation for public health interventions and research. 1st ed. San Francisco: Jossey-Bass; 2002. p. 1–23.
32. Farmer T, Robinson K, Elliott SJ, Eyles J. Developing and implementing a triangulation protocol for qualitative health research. Qual Health Res. 2006;16(3):377–94.

For peer review only - http://bmjopen.bmj.com/site/about/guidelines.xhtml

Figure 1. Flash UK study flow chart

For peer review only

For peer review only - http://bmjopen.bmj.com/site/about/guidelines.xhtml

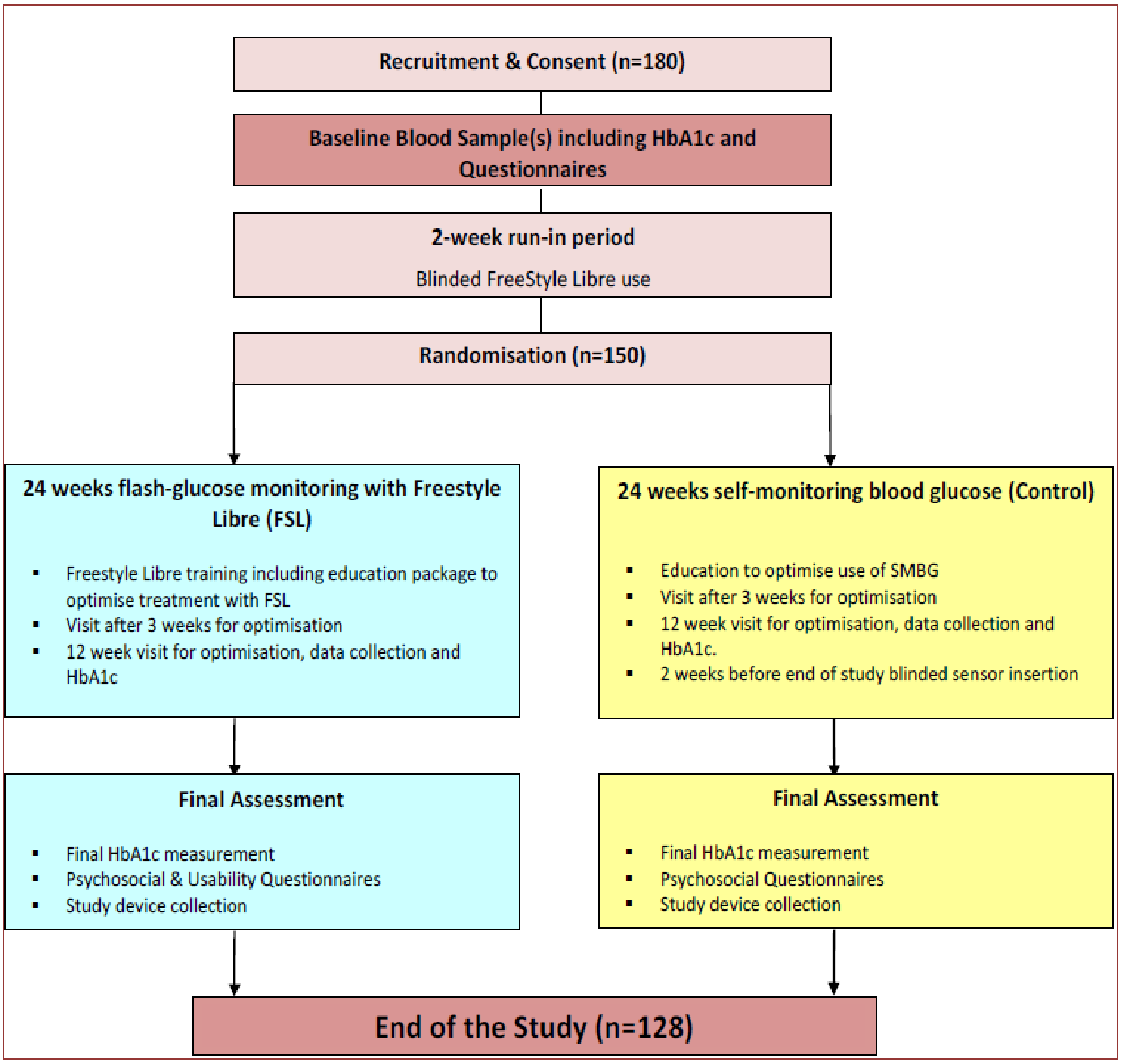
Recruitment & Consent (n=180)
Baseline Blood Sample(s) including HbA1c and Questionnaires
2-week run-in period
Blinded FreeStyle Libre use
Randomisation (n=150)
24 weeks flash-glucose monitoring with Freestyle Libre (FSL)
Freestyle Libre training including education package to optimise treatment with FSL
Visit after 3 weeks for optimisation
12 week visit for optimisation, data collection and HbA1c
24 weeks self-monitoring blood glucose (Control)
Education to optimise use of SMBG
Visit after 3 weeks for optimisation
12 week visit for optimisation, data collection and HbA1c.
2 weeks before end of study blinded sensor insertion
Final Assessment
Final HbA1c measurement
Psychosocial & Usability Questionnaires
Study device collection
Final Assessment
Final HbA1c measurement
Psychosocial Questionnaires
Study device collection
End of the Study (n=128)

only

Supplementary File

Flash-glucose monitoring with the FreeStyle Libre 2 compared to self-monitoring of blood glucose in sub-optimally controlled type 1 diabetes: the FLASH-UK randomised controlled trial protocol

Contents

1. Model Participant Information Sheet (Page 2)
2. Model Consent form (Page 18)
3. Data Sharing Plan (Page 20)

For peer review only

1. Model Participant Information Sheet

Flash-glucose monitoring in sub-optimally controlled Type 1 diabetes (FLASH-UK)

IRAS ID: 257593

For peer review only

Chief Investigator

Dr Lala Leelarathna

Lalantha.Leelarathna@mft.nhs.uk

Principal Investigators for local site

Flash-glucose monitoring in sub-optimally controlled Type 1 diabetes (FLASH-UK)

We would like to invite you to take part in a research study. Before you decide, it is important for you to understand why the research is being done and what it will involve. Please take time to read the following information carefully and discuss it with others if you wish.

- **Part 1 tells you the purpose of this study and what will happen to you if you take part.**
- **Part 2 gives you more detailed information about the conduct of the study.**

Ask us if there is anything that is not clear or if you would like more information. Take time to decide whether or not you wish to take part.

Part 1

Introduction

In type 1 diabetes controlling blood glucose levels is very important for good health. A number of research studies have shown that having high glucose levels in diabetes can lead to long-term problems. In order to lower the risk of these complications, maintaining glucose as close to normal range as possible is recommended. However, the achievement of near-normal glucose levels can be extremely difficult.

Currently the dose of prescribed insulin depends on the results of finger stick testing. The more frequent the testing, the easier it is to adjust the insulin dose. However many people struggle to perform frequent blood glucose tests due to pain and inconvenience.

For peer review only

The FreeStyle Libre 2 flash glucose monitor is a device which makes monitoring of glucose levels easier. It continuously monitors the glucose levels and you are able to see the level and trend by scanning the disc, worn on your arm, with a hand held device.

What is the purpose of the study?

The FreeStyle Libre device has been shown as a safe replacement for blood glucose monitoring in people with well controlled diabetes. However, there are no randomised controlled studies in adults with type 1 diabetes demonstrating improvements in HbA1c levels.

The purpose of this study is to assess the benefit of using FreeStyle Libre 2 flash glucose monitoring system, in terms of improving glucose levels as measured by HbA1c. The study will also assess the acceptability and the impact of the system on daily living with diabetes.

Why have I been invited?

You have been invited because you have Type 1 diabetes and have an HbA1c level between 58mmol/mol (7.5%) and 97mmol/mol (11%) and use either multiple daily insulin injections or use an insulin pump for controlling your blood glucose. Up to 180 participants aged 16 and above with type 1 diabetes will be involved in this project.

If you agree to take part in the study then you will be randomly allocated to either continue usual finger-stick blood glucose monitoring or Flash glucose monitoring with the FreeStyle Libre 2 device for 6 months. During this time you will either attend 5-7 research clinic visits or participate in 5-7 virtual consultations using video calls (or a combination of the two with telephone calls allowed for some visits) for data collection, device training and support to manage your diabetes. You can decide whether to attend clinic visits or virtual assessments but this will also depend on whether your hospital is operating normal clinics because of the COVID-19 pandemic.

Do I have to take part in this research?

No. It is up to you to decide whether or not to take part. If you do, you will be given more detailed information and will be asked to sign a consent form. You are still free to withdraw at any time and

For peer review only - http://bmjopen.bmj.com/site/about/guidelines.xhtml

without giving any reason. A decision to withdraw at any time, or a decision not to take part, will not affect the standard of care you receive.

What will happen to me if I take part?

The study consists of 5-7 visits with the diabetes research team (either in person or by virtual consultation) over a 6 month period. During these visits you will be supported to improve your diabetes control. Blood tests are done three times during this 6 month period. If you attend virtual consultations the information sheet / consent form (for visit 1), necessary questionnaires or blood collection kit will be provided by post before these take place and they can be returned by pre-paid postal delivery.

The following sections describe the study visits in detail.

Visit 1 - Recruitment visit (up to 2 hours)

Once you have had sufficient time to read this information sheet, if you would like to hear more about the study or would like to participate, you will be invited to attend the recruitment visit (either in person or by virtual consultation). You will be provided with detailed information about the study and any questions you have will be answered. If you decide to participate, you will be asked to sign a consent form. By signing the form you are telling us that you understand what you have read and discussed, consent to take part in the research project and consent to the use of your information as described.

If would like to consider joining the trial by virtual consultation, you will receive the consent form along with this information sheet by post before the recruitment visit is scheduled. Any questions you have can be answered by email, telephone or video consultation. If you decide to participate, you will need to provide initials to each declaration box on the consent form, sign it and return it to the research team in a pre-paid envelope. Once the research team receive your fully completed consent form, they will schedule your virtual recruitment consultation. At the start of the virtual recruitment consultation, the investigator will ask if you are happy to proceed and they will sign / date the consent form. A copy of the fully completed consent form will be returned to you. Once you receive this, you can let the research team know before or at your next visit.

For peer review only - http://bmjopen.bmj.com/site/about/guidelines.xhtml

At recruitment visit, if you consent to take part the following data will be captured:

History: medical and diabetes history, current medications and insulin therapy recorded.

Measurements: Height, weight (if possible).

Blood test : HbA1c, (less than 10 mls). If conducted as a virtual visit, we will send you a pre-paid pack to collect blood into a small tube from a finger-stick. This can be returned by post in the pre-paid envelop provided.

Questionnaires: to assess a range of measures which include diabetes treatment satisfaction, diabetes distress, quality of life, fear of injections/testing, eating behaviours, hypoglycaemia. If conducted as a virtual consultation, we will send you a pre-paid envelope to return any questionnaires that cannot be completed by video call.

Personal Data: Initials, Date of Birth, Gender & Full Postcode.

If you intend to become pregnant within the next six months then you should notify the research team. If you are pregnant at the recruitment visit, or should you become pregnant during the study, you will not be able to take any further part.

As with many research studies, in order to proceed with the study certain recruitment criteria need to be met. In the event you don't meet the recruitment criteria your participation in the study will end after visit 1.

Visit 2 (Or combined with visit 1) – Blinded flash glucose monitor insertion (30 minutes)

Within 1 to 2 weeks of visit 1 (or at the same time as visit 1 if easier and recruitment criteria are met), a glucose sensor will be inserted to collect information about glucose data. The sensor looks the same as the Freestyle Libre sensor and is placed in the same area. The purpose of this is to compare results at the end with the results at the beginning. In order to avoid any change in behaviour or insulin doses, glucose sensor data will not be visible to you (Blinded sensor) during these first 2 weeks. It is important that you do not try to obtain the blinded glucose data as this will affect the comparison of before and after data. You are asked to wear the sensor for 14 days. If this visit is conducted as a virtual consultation we will send you the sensor and reader for self-insertion and provide guidance on how to apply the sensor. After 14 days you will need to post the sensor and reader back to us in a pre-paid envelope provided.

For peer review only - http://bmjopen.bmj.com/site/about/guidelines.xhtml

Visit 3 – Randomisation, start of study treatment & education (up to 2 hours)

During Visit 3, we will check that you were comfortable wearing the flash glucose monitor for the 14 days. (To proceed with the study the blinded glucose sensor should have been worn for a minimum of 10 days'). If you are happy to proceed you will be randomly assigned to either flash glucose monitoring with the FreeStyle Libre 2 device for 6 months or continuing with finger-stick glucose tests for 6 months.

Visit 3:

Randomisation: to either flash glucose monitoring or finger-stick glucose tests for the next 6 months.

Education and support: If you are allocated to flash glucose monitoring, the research team will spend some time demonstrating how to insert the device, what the data means and how you safely use the device. This will include a discussion of how to use the device to review data and make adjustments to your insulin therapy. You will be shown how to download and review data at home. If you are continuing with finger-stick blood tests the study team will review your diabetes therapy and support you to make improvements. For those attending virtual consultations and who are assigned to the flash glucose monitoring arm, the research team will send you all the devices you need and easy to follow instructions and links to self-help videos. The Research team will conduct additional training through video consultation as required.

Given participant diary: to record information about insulin doses and carbohydrate intake in last 5 days before study visits 4, 5 and 7. If you are attending virtual consultations, the research team will send diaries for you to complete and these can be returned by pre-paid postal delivery or scanned and returned by email.

Downloading of devices: data from your glucose meter (and insulin pump if you are on one) will be downloaded where possible.

Visit 4 – Data review and education support (up to 1 hour)

For peer review only - http://bmjopen.bmj.com/site/about/guidelines.xhtml

Four weeks after visit 3 you will be asked to come to the clinic or attend a virtual clinic consultation, with your glucose meter (flash glucose reader or blood glucose meter) and the completed diary which captures your insulin doses and carbohydrate intake. The research team will work with you to review your insulin therapy, supporting you to adjust your insulin to improve your diabetes control. Data from your glucose meters (and insulin pump if you are on one) will be downloaded to secure web-based programmes similar to what is used in clinical practice.

Visit 5 - Data review and education support (up to 2 hours)

Eight weeks after visit 4 you will be seen in clinic or by virtual clinic consultation. The purpose of this visit is to provide ongoing support and training to help you improve your diabetes control. You are required to bring your glucose meters and diary with insulin doses. A HbA1c blood test will also be performed at this visit (blood sample collected at home and returned by pre-paid envelope for those attending virtual consultations). Data from your glucose meters (and insulin pump if you are on one) will be downloaded to secure web-based programmes similar to what is used in clinical practice.

Visit 6 – Finger-stick glucose testing group only: blinded sensor (<30 minutes)

If you are allocated to the finger-stick glucose testing group you will be asked to return to clinic or attend a virtual consultation 10 weeks after visit 5 to have a blinded sensor attached. If conducted as a virtual consultation, we will send you the devices you need and easy to follow instructions and self-help videos (like visit 1). The blinded FreeStyle Libre sensor will be worn for 2 weeks. This will allow the collection of glucose data to see how your diabetes control has changed compared to your glucose control when you entered the study. After 14 days you will need to post the sensor and reader back to us by pre-paid envelope if you attended a virtual consultation.

Visit 7 - End of study visit (upto 2 hours)

Approximately 12 weeks after Visit 5 (or 2 weeks after visit 6 for the finger-stick glucose testing group), you will be asked to attend the clinic or virtual consultation. This will be the final visit. Data from your glucose meters (and insulin pump if you are on one) will be downloaded to secure web-based programmes similar to what is used in clinical practice where possible. We will also collect paper diaries.

For peer review only - http://bmjopen.bmj.com/site/about/guidelines.xhtml

During visit 7 the following occurs:

Blood test: HbA1c (blood sample collected at home for virtual consultations).

Questionnaires: same as those completed at the start of the study. In addition if you were randomised to Flash glucose monitoring arm, you will be invited to complete two additional questionnaires to capture your experience and expectations during the study (If you attend a virtual consultation any questionnaires that cannot be completed will be provided for return in a pre-paid envelope).

Data download and review: to support you to improve glucose control.

Weight measured: where possible.

Communication with research team for advice and support by e-mail

If you choose to communicate with us by e-mail, we will use a dedicated e-mail account as a way to communicate with you. We will use reasonable means to protect the security and confidentiality of e-mail information sent and received. The research team will take every care to remove any identifying material from the responses you provide as early as possible. Likewise, individuals' responses will be kept confidential by the researcher and participants will not be identified in the reporting of the research. However, the researcher cannot guarantee the confidentiality or anonymity of material transferred by email or the internet. There are known and unknown risks that may affect privacy when using e-mail to communicate. These risks include, but are not limited, to:

- E-mail can be forwarded, printed, and stored in numerous paper and electronic forms and be received by many intended and unintended recipients without my knowledge or agreement.
- E-mail may be sent to the wrong address by any sender or receiver.
- E-mail is easier to forge than handwritten or signed papers.
- Copies of e-mail may exist even after the sender or the receiver has deleted his or her copy.
- E-mail service providers have a right to keep and inspect emails sent through their systems.
- E-mail may be intercepted and read during transmission without detection or authorisation.
- E-mail is not a secure way of corresponding and it is advisable not to transfer any sensitive information in this format.
- E-mail can spread computer viruses.

Choosing to participate in the trial by virtual consultation

For peer review only - http://bmjopen.bmj.com/site/about/guidelines.xhtml

If you choose to participate in the trial by use of audio / video communication, we will use a dedicated videoconferencing account as a way to communicate with you. We will use reasonable means to protect the security of the connection and virtual consultations will not be recorded. However, the researcher cannot guarantee the confidentiality or anonymity of material transferred over the internet and stored within devices. There is an increased security risk that your health information may be intercepted or disclosed to third parties when using video or audio communications tools. To help us keep your information safe and secure, you can:

- Understand that this method of communication is not secure in the same way as a private appointment in an exam room.
- Use a private computer / device (i.e., not an employer's or third party's computer / device) and a secure internet connection. For example, using a personal computer or tablet is more secure than at a library, and your access to the Internet on your home network will generally be more secure than an open guest Wi-Fi connections.

By providing your information, you agree to let us collect, use, or disclose your personal health information through video or audio communications (while following applicable privacy laws) in order to provide you with care. In particular, the following means of electronic communication may be used for videoconferencing, including Skype, Microsoft Teams and Zoom.

Will I be reimbursed?

There is no payment for taking part in the study. If you are randomised to the flash glucose monitoring arm then the sensors will be provided to you for 6 months, free of charge. The blinded sensors that will be provided at Visit 1 or 2 and at visit 6 (those assigned to finger-stick glucose testing) will also be provided free of charge. Reasonable travelling expenses will be reimbursed if you attend any visits in person at the study clinics.

What are the possible risks of taking part?

During this study, we will support you to improve your glucose control. Sometimes when improving glucose control you may experience a 'hypo', similar to the ones that happen normally in everyday life. We will ask you to treat it in the same way you normally treat a hypo. There is a risk of possible mild to moderate hyperglycaemia (high glucose) which is similar to the risk that a person with type 1 diabetes experiences on a daily basis. There is also a risk of hyperglycaemia leading to diabetic ketoacidosis (for example if the pump is blocked for a significant time or if you miss insulin doses or

For peer review only - http://bmjopen.bmj.com/site/about/guidelines.xhtml

become unwell); however this risk is low and similar to the risk that a person with type 1 diabetes experiences.

You will have to attach a small glucose sensor on the upper arm. The sensor needs to be changed every 14 days. Inserting the sensor has a low risk of developing a local skin reaction or infection. Some bruising, itchiness, redness and bleeding at the site of sensor insertion may occur.

In addition, when the blood samples are taken for the study, some bruising or minor discomfort may also occur at the site where the blood was taken.

What are the possible benefits of taking part?

You will have regular contact with the study team who will support you to improve your glucose control and this may have benefits beyond the study period. Results of the study may pave the way for better access for flash glucose monitoring for people living with type 1 diabetes.

Who should I talk to if I have any questions or concerns?

If you have any questions regarding this study, please contact one of the following people:

Contact details of the local clinical study team

On behalf of the Study Team, we would like to thank you for taking the time to consider participating in this important research programme.

This completes part 1 of the Information Sheet.

For peer review only

If the information in Part 1 has interested you and you are considering participation, please continue to read the additional information in Part 2 before making any decision.

For peer review only

For peer review only - http://bmjopen.bmj.com/site/about/guidelines.xhtml

Part 2

What if relevant new information becomes available?

You will be informed as soon as possible if any new information becomes available during the course of the study that may affect your willingness to participate in the study. If you decide not to continue, the study doctor will arrange for your care to continue.

What will happen if I don't want to carry on with the study?

If you decide not to participate in this study, it will not affect your future management in any way. If you agree to take part, please remember that you are free to withdraw at any time without explanation. All you need to do is tell us. If you withdraw from the study, we will destroy all your identifiable samples, but we will need to use the data collected up to your withdrawal.

What if there is a problem?

If you have a concern about any aspect of this study, please ask to speak to the researchers who will do their best to answer your questions (see contact details above). If you are unhappy about the conduct of the study and wish to complain, you can do this through (name and contact details of the local patient advice and liaison service – institution specific).

In the unlikely event that something does go wrong and you are harmed during the research study, appropriate healthcare arrangements will be made. Healthcare arrangements may include advice from clinical members of the study team or your local diabetes clinic team, or use of emergency health services. There are no special compensation arrangements unless this was due to the negligence of one of the doctors or nurses. In this case you may have grounds for legal action for compensation but you may have to pay your legal costs. The normal hospital complaints mechanism will still be available to you.

Under what circumstances might the study be stopped?

The study may be stopped, or you will be asked to withdraw from the study, under the following circumstances:

For peer review only - http://bmjopen.bmj.com/site/about/guidelines.xhtml

- Serious harm to any participant in the study
- You have significant difficulties using the study devices during the training period(s)
- You develop significant allergy to the plaster holding the sensor
- Failure to follow the instructions of the study team and misuse of the study devices
- Decision by the study team, or the sponsor, that stopping the study is in your best medical interest
- Pregnancy, planned pregnancy, or breast feeding
- Technical reasons (e.g. moving home outside the region)

Should the study be stopped for any reason, then you will be informed as soon as possible and your doctor will arrange for your care to continue.

Will my GP be informed?

We will inform your GP of your participation in this study after having gained your consent for this.

Will my taking part in the study be kept confidential?

Strict confidentiality will be maintained at all times. All collected written data will be kept in a locked cupboard, accessible only by the research team.

Only the researchers who are directly involved in the study, the study sponsor (Manchester University NHS Foundation Trust), the study monitor and regulatory authority who will be involved in monitoring and auditing of the project will have access to the identifiable data, as permitted by applicable laws and regulations. By signing the written informed consent form, you are authorising such access. Anonymised data collected during the study may also be sent to associated researchers in EU and USA where the laws do not protect your privacy to the same extent as the law in the UK. We will however take all reasonable steps to protect your privacy. Data will be stored for 15 years and will be disposed of securely thereafter.

For peer review only

Compliance with General Data Protection (GDPR) Rules

Manchester University NHS Foundation Trust is the sponsor for this study based in the United Kingdom. We will be using information from you and/or your medical records in order to undertake this study and will act as the data controller for this study. This means that we are responsible for looking after your information and using it properly. Manchester University NHS Foundation Trust will keep identifiable information about you [for three years after the study has finished].

Your rights to access, change or move your information are limited, as we need to manage your information in specific ways in order for the research to be reliable and accurate. If you withdraw from the study, we will keep the information about you that we have already obtained. To safeguard your rights, we will use the minimum personally-identifiable information possible.

You can find out more about how we use your information at https://research.cmft.nhs.uk/getting-involved/gdpr-and-research and by contacting our Data Protection Officer at dpo@mft.nhs.uk.

[Name of NHS hospital] will collect information from you and/or your medical records for this research study in accordance with our instructions.

[Name of NHS hospital] will keep your name, [NHS number] and contact details [add other identifiers] confidential and will not pass this information to Manchester University NHS Foundation Trust. [Name of NHS hospital] will use this information as needed, to contact you about the research study, and make sure that relevant information about the study is recorded for your care, and to oversee the quality of the study. Certain individuals from Manchester University NHS Foundation trust, Manchester Clinical Trials Unit and regulatory organisations may look at your medical and research records to check the accuracy of the research study. Manchester University NHS Foundation Trust will only receive information without any identifying information. The people who analyse the information

For peer review only - http://bmjopen.bmj.com/site/about/guidelines.xhtml

will not be able to identify you and will not be able to find out your name, [NHS number] or contact details.

[Name of NHS hospital] will keep identifiable information about you from this study for three years after the study has finished.

What will happen to any samples I give?

As above, blood samples collected during this study will be labelled and sent to the lab for processing, in line with standard accredited practice. Once the measurements have been completed the samples will be disposed of securely.

What will happen to the data collected?

After each visit fully anonymised data will be entered onto a computer and will be used to analyse the results once the study is finished. These study results may be presented at scientific meetings or published in a scientific journal. In addition, fully anonymised data may be shared with researchers and collaborating partners.

Your personal details will be kept for up to 3 years after the end of the study so that we can inform you the results of the study.

Who is organising and funding the research?

The study will be coordinated from the Manchester Clinical Trials Unit, University of Manchester . The research study has been funded by Diabetes UK charity. The sponsor of the study is the Manchester University NHS Foundation Trust.

Who has reviewed the study?

Before any research goes ahead it has to be checked by an Ethics Committee. This project has been reviewed by the Greater Manchester West Research Ethics Committee.

For peer review only

For peer review only - http://bmjopen.bmj.com/site/about/guidelines.xhtml

If I agree to join the study

The study will be explained to you in more detail during the recruitment visit. During this visit you will be able to ask questions and voice any queries. Once you have agreed to take part we will ask you to sign a consent form. The study team will then arrange the dates for your training and hospital visits.

What will happen at the end of the study?

At the end of this study you will go back to using your usual glucose monitoring device. Your diabetes care will then be looked after by your own diabetes team.

This completes part 2 of the Information Sheet. Thank you for your time and interest in our research.

(site/institution address)

For peer review only

2. Consent form

PARTICIPANT CONSENT FORM

Title of Project: Flash-glucose monitoring in sub-optimally controlled type 1 diabetes (FLASH-UK), IRAS ID 257593

Statement	Please Provide Initials in Each Box
Name of Principal Investigator: Dr …………………………………………….	
I confirm that I have read and understood the information sheet version …….. dated ………….. for the above study and have had the opportunity to consider the information and I am satisfied with the answers I have been given.	☐
I understand that my participation is voluntary and that I am free to withdraw at any time, without giving any reason, without my medical care or legal rights being affected in any way.	☐
I give consent for the taking of blood samples.	☐
If I want to change my mind in the future and withdraw my consent for these studies, then I understand that, if I request it, the identifiable data collected will be destroyed. Unidentifiable data would be retained.	☐
I give consent to my GP being informed of my participation in this study.	☐
I give consent to the sharing of anonymised research data arising from this study with other study partners in Europe and USA.	☐
If I choose to communicate with the research team via email / virtual consultation, I acknowledge the associated risks as outlined in the information sheet.	☐

For peer review only - http://bmjopen.bmj.com/site/about/guidelines.xhtml

I understand that relevant sections of my medical notes and data collected during the study may be looked at by individuals from the NHS Trust, the sponsor (or their delegate) and regulatory bodies for audit or monitoring purposes. ☐

I agree to return the study devices at the end of the study or earlier if consent is withdrawn. ☐

I agree to follow instructions from the study team and take part in the study. ☐

Name of patient	Date	Signature
Name of Researcher taking consent	Date	Signature

Copies: *1 for patient; 1 for researcher; 1 to be kept with hospital notes*

For peer review only

3. Data Sharing plan

Data Sharing Plan – FLASH-UK study	
Individual participant data availability (anonymised)	Yes
What data will be shared	Individual participant data that underlie the results reported in the primary study manuscript after deidentification (text, tables, figures and appendices)
What other documents will be available	The study protocol, Statistical analysis plan, Informed consent form, data dictionary and statistical code.
Data availability	Beginning 6 months and ending 3 years following article publication
With whom	Researchers who provide a methodologically sound proposal not overlapping with any planned secondary publications from the research team.
For what type of analyses	To achieve aims in the approved proposal
By what mechanism will data be available	Proposals should be directed to Chief Investigator who will discuss such requested with the Trial Management Group (TMG). Lalantha.leelarathna@mft.nhs.uk.<br><br>To gain access, data requesters will need to sign a data access agreement. Data will be available for 3 years.

For peer review only - http://bmjopen.bmj.com/site/about/guidelines.xhtml

STANDARD PROTOCOL ITEMS: RECOMMENDATIONS FOR INTERVENTIONAL TRIALS

SPIRIT 2013 Checklist: Recommended items to address in a clinical trial protocol and related documents*

Section/item	Item No	Description	Page number
**Administrative information**			
Title	1	Descriptive title identifying the study design, population, interventions, and, if applicable, trial acronym	1
Trial registration	2a	Trial identifier and registry name. If not yet registered, name of intended registry	2
	2b	All items from the World Health Organization Trial Registration Data Set	
Protocol version	3	Date and version identifier	3
Funding	4	Sources and types of financial, material, and other support	19
Roles and responsibilities	5a	Names, affiliations, and roles of protocol contributors	3, 19
	5b	Name and contact information for the trial sponsor	3
	5c	Role of study sponsor and funders, if any, in study design; collection, management, analysis, and interpretation of data; writing of the report; and the decision to submit the report for publication, including whether they will have ultimate authority over any of these activities	19
	5d	Composition, roles, and responsibilities of the coordinating centre, steering committee, endpoint adjudication committee, data management team, and other individuals or groups overseeing the trial, if applicable (see Item 21a for data monitoring committee)	14
**Introduction**			
Background and rationale	6a	Description of research question and justification for undertaking the trial, including summary of relevant studies (published and unpublished) examining benefits and harms for each intervention	5-6
	6b	Explanation for choice of comparators	5

For peer review only

Objectives	7	Specific objectives or hypotheses	6
Trial design	8	Description of trial design including type of trial (eg, parallel group, crossover, factorial, single group), allocation ratio, and framework (eg, superiority, equivalence, noninferiority, exploratory)	6
**Methods: Participants, interventions, and outcomes**			
Study setting	9	Description of study settings (eg, community clinic, academic hospital) and list of countries where data will be collected. Reference to where list of study sites can be obtained	6-7
Eligibility criteria	10	Inclusion and exclusion criteria for participants. If applicable, eligibility criteria for study centres and individuals who will perform the interventions (eg, surgeons, psychotherapists)	16
Interventions	11a	Interventions for each group with sufficient detail to allow replication, including how and when they will be administered	7
	11b	Criteria for discontinuing or modifying allocated interventions for a given trial participant (eg, drug dose change in response to harms, participant request, or improving/worsening disease)	9
	11c	Strategies to improve adherence to intervention protocols, and any procedures for monitoring adherence (eg, drug tablet return, laboratory tests)	8,18, table 2
	11d	Relevant concomitant care and interventions that are permitted or prohibited during the trial	16
Outcomes	12	Primary, secondary, and other outcomes, including the specific measurement variable (eg, systolic blood pressure), analysis metric (eg, change from baseline, final value, time to event), method of aggregation (eg, median, proportion), and time point for each outcome. Explanation of the clinical relevance of chosen efficacy and harm outcomes is strongly recommended	8,17, table 1
Participant timeline	13	Time schedule of enrolment, interventions (including any run-ins and washouts), assessments, and visits for participants. A schematic diagram is highly recommended (see Figure)	18, table 2

For peer review only

For peer review only - http://bmjopen.bmj.com/site/about/guidelines.xhtml

Sample size	14	Estimated number of participants needed to achieve study objectives and how it was determined, including clinical and statistical assumptions supporting any sample size calculations	9/10
Recruitment	15	Strategies for achieving adequate participant enrolment to reach target sample size	10
**Methods: Assignment of interventions (for controlled trials)**			
Allocation:			
Sequence generation	16a	Method of generating the allocation sequence (eg, computer-generated random numbers), and list of any factors for stratification. To reduce predictability of a random sequence, details of any planned restriction (eg, blocking) should be provided in a separate document that is unavailable to those who enrol participants or assign interventions	10
Allocation concealment mechanism	16b	Mechanism of implementing the allocation sequence (eg, central telephone; sequentially numbered, opaque, sealed envelopes), describing any steps to conceal the sequence until interventions are assigned	10
Implementation	16c	Who will generate the allocation sequence, who will enrol participants, and who will assign participants to interventions	10
Blinding (masking)	17a	Who will be blinded after assignment to interventions (eg, trial participants, care providers, outcome assessors, data analysts), and how	10
	17b	If blinded, circumstances under which unblinding is permissible, and procedure for revealing a participant's allocated intervention during the trial	na
**Methods: Data collection, management, and analysis**			
Data collection methods	18a	Plans for assessment and collection of outcome, baseline, and other trial data, including any related processes to promote data quality (eg, duplicate measurements, training of assessors) and a description of study instruments (eg, questionnaires, laboratory tests) along with their reliability and validity, if known. Reference to where data collection forms can be found, if not in the protocol	protocol
	18b	Plans to promote participant retention and complete follow-up, including list of any outcome data to be collected for participants who discontinue or deviate from intervention protocols	10

For peer review only

Data management	19	Plans for data entry, coding, security, and storage, including any related processes to promote data quality (eg, double data entry; range checks for data values). Reference to where details of data management procedures can be found, if not in the protocol	11
Statistical methods	20a	Statistical methods for analysing primary and secondary outcomes. Reference to where other details of the statistical analysis plan can be found, if not in the protocol	11/12
	20b	Methods for any additional analyses (eg, subgroup and adjusted analyses)	11/12/13
	20c	Definition of analysis population relating to protocol non-adherence (eg, as randomised analysis), and any statistical methods to handle missing data (eg, multiple imputation)	11/12
**Methods: Monitoring**			
Data monitoring	21a	Composition of data monitoring committee (DMC); summary of its role and reporting structure; statement of whether it is independent from the sponsor and competing interests; and reference to where further details about its charter can be found, if not in the protocol. Alternatively, an explanation of why a DMC is not needed	14
	21b	Description of any interim analyses and stopping guidelines, including who will have access to these interim results and make the final decision to terminate the trial	NA
Harms	22	Plans for collecting, assessing, reporting, and managing solicited and spontaneously reported adverse events and other unintended effects of trial interventions or trial conduct	9,14
Auditing	23	Frequency and procedures for auditing trial conduct, if any, and whether the process will be independent from investigators and the sponsor	14
**Ethics and dissemination**			
Research ethics approval	24	Plans for seeking research ethics committee/institutional review board (REC/IRB) approval	15
Protocol amendments	25	Plans for communicating important protocol modifications (eg, changes to eligibility criteria, outcomes, analyses) to relevant parties (eg, investigators, REC/IRBs, trial participants, trial registries, journals, regulators)	15
Consent or assent	26a	Who will obtain informed consent or assent from potential trial participants or authorised surrogates, and how (see Item 32)	8
	26b	Additional consent provisions for collection and use of participant data and biological specimens in ancillary studies, if applicable	NA

For peer review only - http://bmjopen.bmj.com/site/about/guidelines.xhtml

Confidentiality	27	How personal information about potential and enrolled participants will be collected, shared, and maintained in order to protect confidentiality before, during, and after the trial	11
Declaration of interests	28	Financial and other competing interests for principal investigators for the overall trial and each study site	19
Access to data	29	Statement of who will have access to the final trial dataset, and disclosure of contractual agreements that limit such access for investigators	11
Ancillary and post-trial care	30	Provisions, if any, for ancillary and post-trial care, and for compensation to those who suffer harm from trial participation	Main protocol
Dissemination policy	31a	Plans for investigators and sponsor to communicate trial results to participants, healthcare professionals, the public, and other relevant groups (eg, via publication, reporting in results databases, or other data sharing arrangements), including any publication restrictions	15
	31b	Authorship eligibility guidelines and any intended use of professional writers	19
	31c	Plans, if any, for granting public access to the full protocol, participant-level dataset, and statistical code	22
**Appendices**			
Informed consent materials	32	Model consent form and other related documentation given to participants and authorised surrogates	Submitted
Biological specimens	33	Plans for collection, laboratory evaluation, and storage of biological specimens for genetic or molecular analysis in the current trial and for future use in ancillary studies, if applicable	NA

*It is strongly recommended that this checklist be read in conjunction with the SPIRIT 2013 Explanation & Elaboration for important clarification on the items. Amendments to the protocol should be tracked and dated. The SPIRIT checklist is copyrighted by the SPIRIT Group under the Creative Commons "Attribution-NonCommercial-NoDerivs 3.0 Unported" license.

For peer review only

BMJ Open

Flash-glucose monitoring with the FreeStyle Libre 2 compared to self-monitoring of blood glucose in sub-optimally controlled type 1 diabetes: the FLASH-UK randomised controlled trial protocol

Journal:	*BMJ Open*
Manuscript ID	bmjopen-2021-050713.R2
Article Type:	Protocol
Date Submitted by the Author:	30-Jun-2021
Complete List of Authors:	Wilmot, Emma; University Hospitals of Derby and Burton NHS Foundation Trust; University of Nottingham Faculty of Medicine and Health Sciences<br>Evans, Mark; Cambridge University, Institute of Metabolic Science<br>Barnard-Kelly, Katharine; Bournemouth University<br>Burns, M; The University of Manchester<br>Cranston, Iain; Portsmouth Hospitals NHS Trust<br>Elliott, Rachel; Manchester Centre for Health Economics<br>Gkountouras, G; The University of Manchester<br>Kanumilli, N<br>Krishan, A; The University of Manchester<br>Kotonya, C; University Hospitals of Derby and Burton NHS Foundation Trust<br>Lumley, S<br>Narendran, P; University of Birmingham College of Medical and Dental Sciences<br>Neupane, Sankalpa; Norfolk and Norwich University Hospital NHS Trust, Elsie Bertram Diabetes Centre<br>Rayman, Gerry; Ipswich Hospital NHS Trust, The Ipswich Diabetes Centre and Research Unit<br>Sutton, Christopher; University of Manchester, Division of Population Health, Health Services Research & Primary Care<br>Taxiarchi, V P; The University of Manchester<br>Thabit, H; Manchester University NHS Foundation Trust; The University of Manchester<br>Leelarathna, L; Manchester University NHS Foundation Trust; The University of Manchester
**Primary Subject Heading**:	Diabetes and endocrinology
Secondary Subject Heading:	Diabetes and endocrinology
Keywords:	General diabetes < DIABETES & ENDOCRINOLOGY, HEALTH ECONOMICS, Clinical trials < THERAPEUTICS, Diabetes & endocrinology < INTERNAL MEDICINE

SCHOLARONE™
Manuscripts

I, the Submitting Author has the right to grant and does grant on behalf of all authors of the Work (as defined in the below author licence), an exclusive licence and/or a non-exclusive licence for contributions from authors who are: i) UK Crown employees; ii) where BMJ has agreed a CC-BY licence shall apply, and/or iii) in accordance with the terms applicable for US Federal Government officers or employees acting as part of their official duties; on a worldwide, perpetual, irrevocable, royalty-free basis to BMJ Publishing Group Ltd ("BMJ") its licensees and where the relevant Journal is co-owned by BMJ to the co-owners of the Journal, to publish the Work in this journal and any other BMJ products and to exploit all rights, as set out in our licence.

The Submitting Author accepts and understands that any supply made under these terms is made by BMJ to the Submitting Author unless you are acting as an employee on behalf of your employer or a postgraduate student of an affiliated institution which is paying any applicable article publishing charge ("APC") for Open Access articles. Where the Submitting Author wishes to make the Work available on an Open Access basis (and intends to pay the relevant APC), the terms of reuse of such Open Access shall be governed by a Creative Commons licence – details of these licences and which Creative Commons licence will apply to this Work are set out in our licence referred to above.

Other than as permitted in any relevant BMJ Author's Self Archiving Policies, I confirm this Work has not been accepted for publication elsewhere, is not being considered for publication elsewhere and does not duplicate material already published. I confirm all authors consent to publication of this Work and authorise the granting of this licence.

For peer review only

Flash-glucose monitoring with the FreeStyle Libre 2 compared to self-monitoring of blood glucose in sub-optimally controlled type 1 diabetes: the FLASH-UK randomised controlled trial protocol

Wilmot E G[1], Evans M L[2], Barnard-Kelly K[3], Burns M[4], Cranston I[5], Elliott R A[6] , Gkountouras G[6], Kanumilli N[7] Krishan A[6], Kotonya C[8], Lumley S[9] , Narendran P[10], Neupane S[11], Rayman G[12], Sutton C J[6], Taxiarchi V P[6], Thabit H[13, 14] , Leelarathna L[13, 14].

[1]University Hospitals of Derby and Burton NHS Foundation Trust, Royal Derby Hospital, Derby, UK.
[2]Wellcome Trust-MRC Institute of Metabolic Science, NIHR Cambridge Biomedical Research Centre, Cambridge University Hospitals and University of Cambridge, Cambridge, UK
[3]Barnard Health, BHR Limited, Hampshire, UK
[4]Manchester Clinical Trials Unit, University of Manchester, Manchester, UK
[5]Academic Department of Diabetes & Endocrinology, Queen Alexandra Hospital, Cosham, Portsmouth, UK
[6]Manchester Centre for Health Economics, Division of Population Health, Health Service Research & Primary Care, University of Manchester, Manchester, UK
[7]Northenden Group Practice, 489 Palatine Road, Northernden, Manchester, UK
[8]University Hospitals of Derby and Burton NHS Foundation Trust, Queen's Hospital Burton, Burton-on-Trent, UK
[9]The Adam Practice, Upton, Poole, Dorset, UK
[10]Institute of Immunology and Immunotherapy, College of Medical and Dental Sciences, University of Birmingham, Edgbaston, Birmingham, UK
[11] Elsie Bertram Diabtes Centre ,Norfolk and Norwich University Hospitals NHS Foundation Trust, Colney Lane, Norwich, UK
[12]Diabetes Research Office, East Suffolk and North Essex NHS Foundation Trust, Ipswich, UK
[13]Manchester Diabetes Centre, Manchester Royal Infirmary, Manchester University NHS Foundation Trust, Manchester Academic Health Science Centre, Manchester, UK
[14]Division of Diabetes, Endocrinology and Gastroentrology, Faculty of Biology, Medicine and Health, University of Manchester, Manchester, UK

Corresponding authors:

Dr Lalantha Leelarathna

Email: Lalantha.Leelarathna@mft.nhs.uk

And

Dr Emma Wilmot

Email: emma.g.wilmot@gmail.com

Word count: 4323 excluding Abstract, Box and Tables (max 4000)
Abstract: 300
Figures: 1, Boxes: 1, Tables 2

For peer review only - http://bmjopen.bmj.com/site/about/guidelines.xhtml

Abstract (max 300 words)

Introduction Optimising glycaemic control in Type 1 diabetes (T1D) remains challenging. Flash glucose monitoring with FreeStyle Libre 2 (FSL2) is a novel alternative to the current standard of care self-monitoring of blood glucose (SMBG). No randomised controlled trials to date have explored the potential benefits of FSL2 in T1D. We aim to assess the impact of FSL2 in people with sub-optimal glycaemic control T1D in comparison with SMBG.

Methods This open-label, multi-centre, randomised (via stochastic minimisation), parallel design study conducted at 8 UK secondary and primary care centres will aim to recruit 180 people age ≥16 years with T1D for >1 year and HbA1c 7.5%-11%. Eligible participants will be randomised to 24 weeks of FSL2 (intervention) or SMBG (control) periods, after two week of blinded sensor wear. Participants will be assessed virtually or in-person owing to the COVID-19 pandemic. HbA1c will be measured at baseline, 12 and 24 weeks (primary outcome). Participants will be contacted at 4 and 12 weeks for glucose optimisation. Control participants will wear a blinded sensor during the last 2 weeks. Psychosocial outcomes will be measured at baseline and 24 weeks. Secondary outcomes include sensor-based metrics, insulin doses, adverse events and self-report psychosocial measures. Utility, acceptability, expectations and experience of using FSL2 will be explored. Data on health service resource utilisation will be collected.

Analysis Efficacy analyses will follow intention-to-treat principle. Outcomes will be analysed using Analysis of Covariance, adjusted for the baseline value of the corresponding outcome, minimisation factors and other known prognostic factors. Both within-trial and life-time economic evaluations, informed by modelling from the perspective of the NHS setting, will be performed.

Ethics and trial registration The study was approved by Greater Manchester West Research Ethics Committee (reference 19/NW/0081). Informed consent will be sought from all participants. The study is registered at ClinicalTrials.gov (NCT03815006).

For peer review only - http://bmjopen.bmj.com/site/about/guidelines.xhtml

Trial registration number: ClinicalTrials.gov NCT03815006 (23/01/2019)

The study website: https://sites.manchester.ac.uk/flash/

Protocol version: 4.0 dated 29/06/2020

Trial Sponsor:

Manchester University NHS Foundation Trust
Division of Research & Innovation
Manchester Royal Infirmary,
Oxford Road
Manchester
M13 9WL

For peer review only

For peer review only - http://bmjopen.bmj.com/site/about/guidelines.xhtml

Strengths and limitations of this study

- Flash-UK is a multi-centre randomised controlled trial of the novel FreeStyle Libre 2 flash glucose monitor over a 6 month follow up period assessing the impact on people living with type 1 diabetes and sub-optimal glucose control, in comparison to self-monitoring of blood glucose, with a primary outcome of HbA1c.

- It is the first randomised study to assess the clinical efficacy & health economic benefits of the Libre 2 device providing high quality data for UK policy makers.

- The integration of a virtual assessment pathway into the trial design ensures eligible candidates are not restricted from participation owing to the COVID-19 pandemic.

- A wide range of secondary outcomes including continuous glucose monitoring data and psychosocial outcomes will provide detailed insight into the impact of this technology on people living with Type 1 diabetes.

- The study is open (unblinded) and conducted in UK National Health Service only. Some findings from the study may only be applicable to UK NHS setting.

For peer review only

Introduction

Type 1 diabetes mellitus (T1D) is one of the most common endocrine conditions. It is estimated that approximately 415 million adults (5-15% type 1 diabetes) and 520 thousand children (95% type 1 diabetes) worldwide suffer from diabetes (1). Despite the availability of therapeutic options such as self-monitoring of blood glucose (SMBG), structured education, rapid-acting insulin analogues and insulin pump therapy, glycaemic control in the majority of people with Type 1 diabetes remains suboptimal (2) and they therefore remain prone to complications associated with high glucose levels, such as kidney failure and blindness (3). In England less than one third of people with type 1 diabetes achieve a HbA1c level <7.5% (4). Studies have shown a strong relationship between the frequency of SMBG and HbA1c, with the National Institute of Clinical Excellence recommending 4-10 checks per day (5, 6). However due to pain, inconvenience and the limited information a moment-in-time glucose value provides, finger-stick glucose monitoring remains a key barrier in achieving near normal glucose levels.

In 2014, the FreeStyle Libre Flash Glucose Monitoring System (FSL) (Abbott Diabetes Care, Oxon, UK) became available as a potential alternative to SMBG. The sensor utilises wired enzyme technology (7) to continuously measure interstitial glucose levels. The arm-worn sensor is scanned using a reader or mobile phone app and provides information on current and previous glucose levels and trends. The IMPACT randomised controlled multicentre European trial was the largest study to evaluate the FSL (8) in 328 participants with well-controlled (HbA1c <= 7.5%, 59 mmol/mol) Type 1 diabetes, a third of whom used CSII therapy. FSL use was associated with improvement in a range of glucose-related outcomes: a 38% reduction in time spent in hypoglycaemia (<3.9 mmol/l). There was an increase in glucose time in range but HbA1c was unchanged. The impact of FSL was also assessed in those with Type 2 diabetes on intensive insulin therapy in a large multi-centre randomised European study of 224 participants (9). Time in hypoglycaemia (<3.9mmol/l) reduced compared with controls by 43% but HbA1c was unchanged. In both randomised controlled trials treatment satisfaction was higher in FSL users and no device-related serious adverse events were reported, suggesting that flash glucose monitoring also offers a safe replacement to SMBG in those with diabetes on intensive insulin therapy.

Subsequently a range of observational studies have demonstrated benefits of FSL use for HbA1c and hypoglycaemia (10-15). Campbell et al. evaluated the use of FSL as a replacement for SMBG in young people (4-17 years) (n=76, 58% CSII users, mean (SD) age 10.3(4.0) years, baseline HbA1c 7.9 (1.0)% (63 mmol/mol), T1D duration 5.4(3.7) years) with Type 1 diabetes in a single-arm European multi-centre trial (16). After 2 weeks' baseline masked wear, participants used FSL for 8 weeks. HbA1c

For peer review only - http://bmjopen.bmj.com/site/about/guidelines.xhtml

significantly improved vs. baseline, -0.4±0.6%, p<0.0001. However, a subsequent 6-month randomised controlled parallel-arm trial of the FSL in 64 participants aged 13-20 years with type 1 diabetes and glycated haemoglobin (HbA_{1c}) ≥9% (≥75 mmol/mol) demonstrated no statistically significant difference between groups for changes in HbA_{1c} at 6 months (adjusted mean -0.2% greater improvement for FSL [95% CI -0.9 to 0.5] [-2.1 mmol/mol (95% CI -9.6 to 5.4)]; p= 0.58) (17). The discrepancy in findings between the observational and randomised controlled trials in the paediatric population highlights the importance of high-quality randomised trials to investigate the benefits of this technology. To date, although some are planned, there has been no published randomised controlled trial to demonstrate the impact of the FSL in adults with Type 1 diabetes and high HbA1c levels (18,19).

More recently, in 2020, the Freestyle Libre 2 (FSL2) launched in Europe. This is very similar to FSL except for an optional additional alarm to alert users when the glucose level falls outside their target range, in addition to improved senor accuracy (20). The FSL2 was launched in the United Kingdom in January 2021. To the best of our knowledge no randomised controlled trials to assess the efficacy of the FSL2 system has been conducted or in progress. No economic evaluation assessing relative costs and benefits has been carried out in this patient group, nor has there been an assessment of patient acceptability. The purpose of the Flash UK study is to address this gap in the evidence and determine whether use of flash glucose monitoring with the FSL2 device will improve HbA1c over a 24-week randomised period compared to SMBG in adults with type 1 diabetes and sub-optimal glycaemic control.

Methods and analysis

Trial design

Flash-UK is an open, multi-centre, randomised (1:1), parallel-group superiority trial, in adults and adolescents (16 years and older) with type 1 diabetes and sub-optimal glycaemic control (HbA1c 7.5% to 11% (59 to 97 mmol/mol), either on continuous subcutaneous insulin infusion (CSII) or multiple daily injections (MDI), contrasting flash glucose monitoring using the FSL2 device with traditional finger-stick SMBG for 24 weeks. The study flow chart is outlined in Figure 1.

Study setting

Eight (one primary and seven secondary) care diabetes services from across England, UK.

1. Diabetes Centres within Manchester University Foundation Trust

For peer review only - http://bmjopen.bmj.com/site/about/guidelines.xhtml

2. Diabetes Centres within University Hospitals of Derby and Burton NHS Foundation Trust
3. University Hospitals Birmingham NHS Foundation Trust
4. Wolfson Adult Diabetes Endocrine Clinic, Cambridge Universities Hospitals, Cambridge
5. Norfolk and Norwich University Hospital, Norwich
6. Queen Alexandra Hospital, Portsmouth
7. Ipswich Hospital, East Suffolk and North Essex NHS Foundation Trust, Ipswich
8. Wareham Surgery (Wareham) and The Adam Practice (Poole), NHS England Primary Care GP Practices

Eligibility criteria

The major eligibility criteria are age ≥16 years with Type 1 diabetes for at least 1 year and a HbA1c between 7.5 and 11%. The full criteria are available in Box 1.

Interventions

FreeStyle Libre 2

This intervention is the CE marked FreeStyle Libre 2 (FSL2) flash glucose monitoring device (Abbott Diabetes Care, Oxon, UK). The FSL2 glucose sensor is an arm worn sensor intended to last for 14 days. The component not directly attached to the patient is the handheld reader and/or mobile phone app which display current and historical glucose data. Education and training about insertion and initiation of the sensor as well as how to use flash-glucose monitoring data for treatment optimisation is also provided. Encouragement is also provided to download data at home to identify pattern recognition. This session, designed to meet the needs of the individual, is conducted by a professional diabetes educator or a member of the study team.

Finger-stick SMBG

This is continuation of usual treatment. Additionally, encouragement will be given to use finger-stick glucose levels to optimise therapy and education about insulin dose adjustments using finger-stick glucose levels will also be provided. Participants in both arms will also receive training on sick day rules and dealing with hypo and hyperglycaemia as required.

Participants will be provided with an information leaflet following the training session, although the information provided will be tailored to the respective intervention. The leaflet provided in conjunction with the FSL2 will include sign-posting to educational videos provided by the Association

For peer review only - http://bmjopen.bmj.com/site/about/guidelines.xhtml

of British Clinical Diabetologists (ABCD) (https://abcd.care/dtn/education) and Bertie online (www.bertieonline.org.uk) in conjunction with the finger-stick SMBG.

Outcomes

The FLASH-UK study is designed to assess an extensive array of clinical, psychosocial and usability outcomes (Table 1). An outline of a dedicated health economic evaluation is provided below. The primary end point is HbA1c level at 24 weeks post-randomisation. Sensor based outcomes comparing the FSL2 with SMBG will be assessed for the last 2 weeks of the intervention period. Prespecified sensor based outcomes include time in the target range (3.9 to 10mmol/l), duration of hypoglycaemia < 3.5 mmol/l (63 mg/dl); < 3.0 mmol/l (54mg/dl); < 2.8 mmol/l (50 mg/dl), duration of hyperglycaemia (>10 mmol/l (180 mg/dL), and >16.7mmol/l (>300 mg/dL), and glucose variability (Standard deviation & coefficient of variation). All the sensor based metrics will also be analysed separately for daytime (7:00-23:00 hours) and night-time (23:00-7:00 hours) in addition to 24 hour period. Insulin usage data will be compared between the two arms. Harms outcomes include the frequency of severe hypoglycaemic episodes as defined by American Diabetes Association, Frequency of significant ketosis events (plasma ketones >3mmol/l) and nature and severity of other adverse events. Information on any other anti-diabetes therapy will be collected.

Questionnaires (Table 1) will be employed at baseline and the end of the study to evaluate participants' responses in terms of quality of life, diabetes distress, needle burden, disordered eating, depression and diabetes treatment satisfaction using EQ-5DL-5L questionnaire, Type 1 Diabetes Distress Scale (DDS), Diabetes fear of injecting and self-testing (D-FISQ) questionnaire, Diabetes Eating Problem Survey (DEPS-R), Diabetes Treatment Satisfaction Questionnaire (DTSQ), Patient Health Questionnaire (PHQ-9) and The Glucose Monitoring Satisfaction Survey (GMSS). Hypoglycaemia burden will be assessed using Clarke questionnaire and Gold score.

Participant timeline & data collection

The study will consist of 6 visits for those in the FSL2 arm and 7 visits in the SMBG arm. Visits 1 and 2 can be conjoined. The study flow chart is shown in Figure 1. Key activities undertaken during each study visit are shown in Table 2.

Due to the COVID-19 pandemic all study visits can be conducted either face to face or virtually (supported by telephone and videoconferencing as appropriate) as indicated in the approved study advertisement and participant information sheet. At visit 1, following informed written consent (model consent form and patient information sheet in supplementary material) by trained members of the research team, medical and diabetes history will be recorded including presence of diabetes

For peer review only - http://bmjopen.bmj.com/site/about/guidelines.xhtml

complications, hypoglycaemia burden including the use of Clarke and Gold questionnaires, use of concomitant diabetes medications, ethnicity, body weight and height measurement; demographic data, insulin therapy, occupation and educational attainment, any history of disordered eating or needle phobia, previous participation in structured education, carbohydrate counting status, use of bolus calculator and patient self-report psychosocial questionnaires will be completed. Blood samples will be taken to measure HbA1c.

At visit 2 the FreeStyle Libre Pro blinded CGM device will be inserted, to be worn for 2 weeks. For participants on the virtual pathway, participant will be taught to self insert the sensor. At Visit 3, participant adherence / tolerance of using the flash-CGM over the preceding 14 days will be assessed. To proceed to randomisation at least 10 of 14 days blinded CGM data must be available. Those who have <10 days data will be provided with a new FreeStyle Libre pro sensor and reader to obtain the minimum data requirements prior to randomisation or discontinue from trial participation if there is a continued adverse reaction to the sensor adhesive.

Participants will be followed up at 4, 12 and 24 weeks post randomisation (+/- 2 weeks). At these visits glucose data will be downloaded and participant diaries which collect information on insulin doses and carbohydrate intake in last 5 days will be collected. Participants in the virtual pathway will be encouraged to upload data from home. Blood tests for HbA1c will also be performed at 12 and 24 weeks after randomisation. Those in the SMBG group will attend an additional visit at 22 weeks to have the blinded CGM sensor fitted to be worn from 22-24 weeks. At 24 weeks, body weight measurement will be made where possible and the participant will be asked to complete questionnaires. In addition, participants in the FSL2 arm will be asked to complete a questionnaire exploring expectations and experience of using FSL2 during the study. Healthcare resource use in primary and secondary healthcare settings will also be collected at 24 weeks.

Adverse events will be routinely collated at study visits. The independent data monitoring committee (IDMC) will be informed of all serious adverse events and any unanticipated adverse device effects that occur during the study and will review compiled adverse event data at periodic intervals.

Sample size

The target effect size (minimally clinically important HbA1c difference) of 0.4% was chosen as this is consistent with other relevant trials (0.5% in REPOSE (21), 0.4% in DIAMOND (22), 0.3% in GOLD (23)). To achieve 90% power using an independent-sample t-test for 24-week HbA1c values (2-tail alpha=0.05, power=0.80, effect size=0.4%, SD=0.8% (23) i.e. Standardised Effect Size = 0.5), 128

For peer review only - http://bmjopen.bmj.com/site/about/guidelines.xhtml

participants with primary outcome data are required. This is inflated to a target of 150 randomised (assuming maximum 15% attrition) and up to 180 recruited (to allow for pre-randomisation losses). The use of ANCOVA with adjustment for baseline values of HbA1c and other minimisation factors should increase power.

Recruitment

Participants will be identified by treating clinicians in each centre and participant information sheet provided. The first study site was activated on the 20/12/2019 and first participant was recruited on the 09/01/2020. Each site will each aim to recruit between 14 and 30 participants up to a total of 180 participants. The study has been advertised via social media and through the official trial website (https://sites.manchester.ac.uk/flash/), providing those who do not usually attend study centres to have the opportunity to participate. As of 31/01/2021, 109 participants had been randomised. It is anticipated that recruitment will be completed by 30/04/2021. In the event that a participant meets withdrawal criteria, sites will engage with participants to seek their verbal agreement to volunteer their continued participation for HbA1c time-points (post randomisation) and exit questionnaires under the intention to treat analysis principle.

Allocation

Sequence generation

Allocation to one of the two intervention arms (24-weeks use of flash-glucose monitoring or 24 weeks use of conventional finger-stick SMBG) will use stochastic minimisation (factors: study centre, baseline HbA1c (7.5%-9.0%; >9.0%-11%), treatment modality (MDI;CSII), prior participation in structured education course (yes; no) and current use of bolus calculator (yes; no)).

Allocation concealment mechanism and its implementation

Allocation will be implemented using the web-based Sealed Envelope™ software (Sealed Envelope Ltd, London, UK) randomisation system. This will be independently managed by Manchester CTU (MCTU) staff. Participants will have their pseudonymised registration details entered into the randomisation system by delegated recruitment site research teams who will also confirm participant eligibility; only then will the system generate an email confirmation with their allocation.

Blinding (masking)

The trial is fully open. It is not possible to blind investigators or participants to the delivery or receipt of the intervention; data collectors and statisticians are also unblinded to treatment allocations.

For peer review only - http://bmjopen.bmj.com/site/about/guidelines.xhtml

Laboratory analysis

Blood samples for the measurement of HbA1c levels will be taken at three different time points: screening, 12 and 24 weeks. This can be completed using the local laboratory (face to face clinic) or at home where the participant will collect a capillary blood sample using a validated home self-test kit (TDL TINY™) and send in a pre-paid envelope to The Doctors Laboratory Ltd (London, UK).

Data management

Confidentiality of participant data shall be observed at all times during the study. Personal details for each participant taking part in the research study and linking them to a unique identification number will be held locally on a study screening log in the Investigator Site File at each of the investigation centres. The study identification number will be used on the case report forms,on all the blood samples that are collected throughout the study and FreeStyle Libre 2 and FreeStyle Libre Pro data submitted to study specific Libreview database (Libreview.com). Names and full addresses will not be used. Electronic data will be stored on password-protected computers. Only members of the research team and collaborating institutions will have password access to the anonymised electronic data. Paper copies of the data will be stored for 15 years. Direct access to the source data will be provided for monitoring, audits, REC review during and after the study. The fully anonymised data may be shared with third parties (EU or non-EU based) for the purposes of advancing management and treatment of diabetes. Standard procedures agreed by the MCTU, Chief Investigator and Clinical Principal Investigators are in place for data review, database cleaning and issuing and resolving data queries.

Analysis

Statistical Methods

All efficacy and safety analyses will be conducted following the intention to treat (ITT) principle in which all randomised participants are analysed in their allocated treatment group whether or not they receive their randomised treatment. All baseline, 12-week and 24-week outcome data will be presented descriptively, both overall and within treatment group, using mean (SD), median (IQR) or frequency (percentage), as appropriate. All statistical tests will use a 2-sided significance level of 5% (unless otherwise specified). All confidence intervals will be presented at a level of 95% and will be two sided. All statistical analyses will be performed using Stata (StataCorp, College Station TX, USA).

For peer review only - http://bmjopen.bmj.com/site/about/guidelines.xhtml

The primary outcome analysis will evaluate between group differences in HbA1c levels at the end of the 24-week treatment period. An analysis of covariance (ANCOVA) model will be used, with 24-week HbA1c as the outcome and trial arm effect as the focus, and with adjustment for baseline HbA1c, and the other baseline variables included in the minimisation allocation algorithm as covariates. If more than 10% HbA1c data are missing at 24 weeks (or a >10% difference between missing data percentages in the two arms) multiple imputation will be used in order to implement a more complete ITT analysis of the substantive ANCOVA model (otherwise this will be performed as a sensitivity analysis, with a complete-case analysis used as the primary analysis). The imputation model will include baseline and 12-week HbA1c, all the baseline variables used in the outcome model and any other recorded variables found to be predictive of missingness in the 24-week outcome in exploratory analyses (via a logistic regression model, with terms included using a 10% significance level). Sensitivity analyses will be performed to (i) examine robustness of how missing data are handled, (ii) examine efficacy of treatment through Complier-Adjusted Causal Effect modelling, (iii) examine impact of data collected outside the visit window, and (iv) examine potential impact of Covid-19 on the primary outcome.

Quantitative secondary outcomes will also be analysed using ANCOVA and binary secondary outcomes will be analysed using logistic regression. In each case, the analysis will be adjusted for the baseline value of the outcome and the adjustment factors used in the model for HbA1C. Harms data will be reported descriptively as frequencies and percentages (%), both overall and by intervention arm.

Subgroup analysis will be performed by the addition of interactions between intervention arm and the subgrouping factor. All subgroup analyses will be performed separately. Planned subgroups are: baseline HbA1c; treatment modality; prior participation in structured education course; age group; education level; hypoglycaemia unawareness; deprivation index quintile; sex; ethnic group.

Further sensitivity and/or subgroup analyses will be performed as appropriate. These together with fuller details of the analyses proposed above, and any additional analyses, will be included in a full Statistical Analysis Plan that will be approved by the Trial Steering Committee (TSC) prior to any analysis of the outcome data.

<u>Economic analysis</u>

The economic evaluation will determine the difference in costs and outcomes generated by the FSL2 device compared with SMBG. The economic evaluation will be conducted from the perspective of NHS/Personal Social Services (PSS) following standard quality design and reporting criteria (24).

For peer review only - http://bmjopen.bmj.com/site/about/guidelines.xhtml

A within-trial cost-utility analysis will compare differences in total costs and differences in quality of life using QALYs derived from the EQ-5D-5L. QALYs will be calculated by attaching available utility weights to the health states generated from the EQ-5D-5L, using area under the curve methods with an assumption of a linear change between time points, controlling for baseline. Person-level costs will be generated for each person in the FSL2 device and SMBG arms from a combination of trial-based resource use with published unit costs, allowing comparison in terms of costs to NHS and PSS (25). Costs will be compared between the two groups using non-parametric methods.

Modelling the potential effect of the intervention on costs and outcomes beyond the trial period will provide a better idea of overall impact as the benefits of controlling HbA1c are likely to be seen after the endpoint of the trial. Therefore, we will carry out an economic evaluation informed by modelling to estimate longer-term benefits and costs, from an NHS/PSS perspective.

A commercially available cost-effectiveness model, the IMS Centre for Outcomes Research and Effectiveness diabetes model version 8.5 (IMS Health, Danbury, CT, USA), will be used for this economic evaluation. The model consists of 15 submodels designed to simulate diabetes-related complications, non-specific mortality and costs over time. It also incorporates the costs and effects of hypoglycaemia, so is particularly well-suited to this study. Two major validation papers on the IMS CDM have been published to date. (26,27) The IMS Core Diabetes Model has also been used in a UK-based recent health technology assessment of CGM commissioned by NICE.(28)

Incremental cost-effectiveness ratios (ICERs) will be calculated in the event of the intervention having either higher costs and better outcomes or lower costs and worse outcomes (no scenarios of dominance, based on QALYs and trial primary outcome). The base case analysis will estimate the mean costs and QALYs across the treatment arms. The overall impact of uncertainty will be assessed by generating cost-effectiveness planes from bootstrapped resamples, and distributional assumptions about the transition probabilities, costs and utility values will be made. Cost-effectiveness acceptability curves (CEACs) will be constructed to show the probability that the intervention is cost-effective for different willingness to pay (WTP) thresholds. Incremental economic analysis using the IMS CORE model will require the model's time horizon to be set to 80 years, which approximates to a lifetime horizon. All costs and effects will be discounted by 3.5%, as per NICE guidance(25,29).

Process evaluation

The process evaluation will be undertaken to explain discrepancies between expected and observed outcomes, to understand how context influences outcomes, and to provide insights to aid

For peer review only - http://bmjopen.bmj.com/site/about/guidelines.xhtml

implementation. Specifically, we will investigate whether treatment is consistent with the behaviour change theories, which underpin it and contextual factors have affected implementation. Process evaluation will use a pipeline logic model, showing causal links between resources, activities and outcomes, integrating the National Institute for Health Behaviour Change Consortium's (NIHBCC's) approach to treatment fidelity (30) and a modified version of Linnan and Steckler's framework for process evaluation.(31) We will describe context qualitatively and take a mixed methods approach to characterising recruitment, reach, dose delivered/received and fidelity, with triangulation between data sources.(32) Free-text response questionnaires will be completed by intervention designers, health professionals and trial participants, and analyses combined with trial data, including FSL2 device utilization and SMBG data which will be analysed descriptively (including the use of appropriate graphical representation), within arms where appropriate, will be synthesised and findings triangulated appropriately.

Study Management

Trial Management Group (TMG)

TMG comprising the Chief Investigator, Co-Investigators, Trial Managers, Trial Statistician, Trial Health Economist, Monitor, and Data Manager will meet quarterly to discuss the operational aspects of the study.

Trial Steering Committee

TSC with an independent chair has been appointed. Independent membership of the TSC includes two clinical members (including the chair), two service users, health economist and statistician; non-independent members are Chief investigator (CI) and Statistician Co-Investigator (only Statistician voting). Other members of the study team including the Sponsor are invited to TSC meetings as observers only.

Independent Data Monitoring Committee

An IDMC comprising a clinical chair, another clinical expert and a statistician has been appointed. The IDMC will be informed of all serious adverse events and any unanticipated adverse device effects/events that occur during the study. The IDMC will review compiled adverse event data at periodic intervals. The IDMC will report to the TSC any safety concerns and recommendations for suspension or early termination of the investigation.

Study Monitoring

For peer review only - http://bmjopen.bmj.com/site/about/guidelines.xhtml

A detailed risk assessment was completed by MCTU and approved by the Sponsor. The procedures, source data transfer modalities and anticipated frequency for monitoring are documented in the Monitoring Plan. Copies of the risk assessment and the Monitoring Plan will be stored in the Trial Master File. The MCTU study monitor will be fully independent of both the Sponsor and Site Principal Investigators.

Authorised representatives of Sponsor, regulatory authority, or an Ethics Committee may perform audits or inspections at the recruiting centres, including source data verification.

Any substantial / non-substantial amendments will be managed by the clinical trials units following an approved quality standard operating procedure. Favourable outcomes will be conveyed to participating sites for implementation following local R&D.

Patient and public involvement

During the grant application process the study protocol received input from several patient groups from Manchester, Derby and Cambridge as well as the INPUT patient advocacy group. One of the study investigators also has Type 1 diabetes as do two members of the Trial Steering Committee.

Ethics and dissemination

The study will be conducted in accordance with the Declaration of Helsinki Ethical Principles for Medical Research involving Human Subjects (October 2000). It was approved by Greater Manchester West Research Ethics Committee on 21/03/2019REC reference 19/NW/0081. All participants will be provided with oral and written information about the trial, including the most common AEs, and the procedures involved in the study before obtaining written informed consent. The study results will be communicated to trial participants and disseminated in peer-review publications and through conference presentations. The data sharing plan is available in Appendix 1.

For peer review only - http://bmjopen.bmj.com/site/about/guidelines.xhtml

Box 1. Key Inclusion and Exclusion Criteria

**Inclusion Criteria**
1. The participant is ≥16 years old<br>2. The participant has type 1 diabetes, as defined by WHO for at least 1 year or is confirmed C-peptide negative if duration of diabetes is < 1 years<br>3. Participant is treated with insulin pump or multiple daily injection for at least 12 weeks and no plans to change treatment modality during next 28 weeks<br>4. The participant is literate in English for safe study conduct<br>5. Screening HbA1c ≥ 7.5% (58.5mmol/mol) and ≤ 11% (97 mmol/mol) based on analysis from local, central or third party external laboratory<br>6. The participant is willing to wear study glucose sensor and scan for glucose levels at regular intervals,<br>7. The participant is willing to follow study specific instructions and improve glucose control<br>8. Female participants of child bearing age should be on effective contraception or not sexually active / no plans for pregnancy
**Exclusion Criteria**
1. Non-type 1 diabetes mellitus including those secondary to chronic disease<br>2. Any other physical disease or people with known severe mental illness (psychotic disorder, bipolar disorder, dementia, substance and alcohol dependence, learning disabilities, depression with active suicidal ideation) which are likely to interfere with the normal conduct of the study and interpretation of the study results as judged by the investigator<br>3. Current users of real-time glucose monitoring sensors or flash-glucose monitoring for more than 4 weeks within last 12 weeks<br>4. Initiation of medications/treatments known to interfere with glucose metabolism (e.g: metformin, SGLT2 inhibitors, GLP-1 agonists, Pramlinatide) within the last 6 weeks or planning to start these medications within the next 6 months (patients on stable treatment is not an exclusion) or current or planned glucocorticoid use other than inhaled/ topical use<br>5. Known or suspected allergy against insulin<br>6. Severe visual impairment<br>7. Complete loss of hypoglycaemia awareness<br>8. Patient receiving dialysis / pre-dialysis based on history<br>9. More than one episode of severe hypoglycaemia as defined by American Diabetes Association (30) in preceding 24 weeks<br>10. Pregnancy, Planned Pregnancy in the next 8 months or breast feeding

For peer review only - http://bmjopen.bmj.com/site/about/guidelines.xhtml

Table 1: Secondary Outcomes

Category	Outcomes	Assessment Time-point
**HbA1c based**	HbA1c<br>HbA1c ≤ 53 mmol/mol (7.0%) [yes/no];<br><br>HbA1c ≤ 59 mmol/mol (7.5%) [yes/no];<br>Reduction in HbA1c ≥5.5 mmol/mol (0.5%) from baseline [yes/no];<br>Reduction in HbA1c ≥ 11 mmol/mol (1.0%) from baseline [yes/no];	Baseline and 12 weeks<br>Baseline, 12 weeks and 24 weeks<br>Baseline, 12 weeks and 24 weeks<br>Baseline, 12 weeks and 24 weeks<br>Baseline, 12 weeks and 24 weeks
**Sensor based (glucose)**	Time spent in the target glucose range 3.9 to 10.0 mmol/l (70 to 180mg/dl),<br>Time spent below target glucose (<3.9mmol/l) (<70mg/dl), < 3.5 mmol/l (63 mg/dl); < 3.0 mmol/l (54mg/dl); < 2.8 mmol/l (50 mg/dl)<br>Time spent above target glucose (10.0 mmol/l) (180 mg/dl), > 16.7 mmol/l) (300mg/dl).<br>Average glucose , Standard deviation, Coefficient of variation<br>AUC of glucose below 3.0mmol/l (54mg/dl)	Baseline and 24 weeks
**Non-sensor based (clinical)**	Daily average total insulin dose<br>Daily average basal insulin dose<br>Daily average bolus dose<br>Average number of boluses of rapid acting insulin per day<br>Frequency of severe hypoglycaemic episodes as defined by American Diabetes Association<br>Frequency of significant ketosis events (plasma ketones >3mmol/l)<br>Nature and severity of other adverse events	Baseline, 12 weeks and 24 weeks
**Psychosocial**	Type 1 Diabetes Distress Scale (T1-DDS)<br>Quality of Life (EQ-5D-5L)<br>Patient Health Questionnaire (PHQ-9)<br>Diabetes fear of injecting and self-testing questionnaire (D-FISQ)<br>The revised Diabetes Eating Problem Survey (DEPS-R)	Baseline and 24 weeks
**Process evaluation (utility and acceptability)**	FSL2 device utilization data, including: average number of scans per day (7:00-23:00 hours), per night (23:00-7:00 hours) and over the full 24-hour period; average number of days of usage per week for the full 24 week intervention<br><br>Number of finger-stick glucose level tests per day<br><br>Diabetes Treatment Satisfaction Questionnaire (DTSQ)<br>Glucose Monitoring Satisfaction Survey (GMSS)	Continuous (FSL arm only)<br><br>Continuous<br><br>Baseline and 24 weeks

For peer review only

For peer review only - http://bmjopen.bmj.com/site/about/guidelines.xhtml

Table 2: Schedule of study visits:

Visit/ contact	Description	Time since randomisation	Start relative to previous / next Visit / Activity**
Visit 1	Recruitment & Screening visit: Consent<br>HbA1c, baseline bloods, baseline questionnaires	-2 to -3 weeks	-
Visit 2	Blinded flash glucose monitor insertion	-2 weeks	Within 1 to 2 weeks of Visit 1. Can coincide with Visit 1
Visit 3	Adherence assessment & Randomisation<br>FSL2 / Self- monitoring of glucose initiation<br>- Education	0 weeks	After 2 weeks of Visit 2
Visit 4	Review data /optimisation<br>Collect participant diary	+4 weeks	After 4 weeks of Visit 3
Visit 5	Review data /optimisation. Data download<br>- HbA1c<br>- Collect participant diary	+12 weeks	After 8 weeks of Visit 5
Visit 6	Blinded flash glucose monitor insertion (Extra visit in SMBG arm)	+22 weeks	After 10 weeks of Visit 5
Visit 7	End of self-monitoring intervention arm<br>- HbA1c.<br>- Questionnaires<br>- Collect participant diary	+24 weeks	2 weeks after Visit 6

For peer review only - http://bmjopen.bmj.com/site/about/guidelines.xhtml

Authors' contributions

LL, EGW and ME conceptualised the study. LL, EGW, ME, IC, PN, SN, RE, CS, HT contributed to the grant application. MB is the Lead Clinical Trial Manager. CS, AK and VT are responsible for statistical analysis. All authors contributed to protocol development at various stages. LL, EGW, ME, IC, PN, SN, HT, GR, SL, NK, CK provides site oversight and are responsible for study conduct at each site. RE & GG are responsible for the health economic analysis. KB is responsible for the process evaluation. EGW & LL wrote the first draft of the manuscript and all authors reviewed and had the opportunity to comment on the content prior to submission. The corresponding author confirms that all co-authors are ICMJE recommendation compliant for the submission of this manuscript. No professional writers have been engaged for the preparation of this manuscript.

Competing interests statement

EGW has received personal fees from Abbott Diabetes Care, Dexcom, Eli Lilly, Insulet, Medtronic, Novo Nordisk, Sanofi Diabetes Care. LL has received personal fees from Abbott Diabetes Care, Dexcom, Insulet, Medtronic, Novo Nordisk, Sanofi Diabetes Care. ME has received personal fees from Abbott Diabetes Care, Eli Lilly, Medtronic, Novo Nordisk, Astra Zeneca, Zucara.SN has received personalised fees from QUIN,Roche. NK has received personal fees from Abbott,Eli Lilly,Novo Nordisk, Astra Zeneca, Napp, Sanofi. PN has acted as a clinical expert for NICE Medtech innovation briefing MIB110 relating to FreeStyle Libre system. GR has received lecture and consultancy fees from Abbott Diabetes UK. MB, KBK, GG, CS, CK, VT, AK, SN, RAE-No competing interests.

Funding statement

This work was supported by Diabetes UK grant number 18/0005836. The device manufacturer played no part in design, conduct or any other aspects of the study. The study devices were paid by the National Health Service (NHS) UK. Work was supported by the NIHR Cambridge Biomedical Research Centre. The University of Cambridge has received salary support for MLE from the National Health Service in the East of England through the Clinical Academic Reserve.

For peer review only

For peer review only - http://bmjopen.bmj.com/site/about/guidelines.xhtml

References

1. International Diabetes Federation, The Diabetes Atlas, Seventh Edition. Brussels:2015. http://www.diabetesatlas.org/, 2015
2. McKnight JA, Wild SH, Lamb MJ, Cooper MN, Jones TW, Davis EA, Hofer S, Fritsch M, Schober E, Svensson J, Almdal T, Young R, Warner JT, Delemer B, Souchon PF, Holl RW, Karges W, Kieninger DM, Tigas S, Bargiota A, Sampanis C, Cherubini V, Gesuita R, Strele I, Pildava S, Coppell KJ, Magee G, Cooper JG, Dinneen SF, Eeg-Olofsson K, Svensson AM, Gudbjornsdottir S, Veeze H, Aanstoot HJ, Khalangot M, Tamborlane WV, Miller KM: Glycaemic control of Type 1 diabetes in clinical practice early in the 21st century: an international comparison. Diabet Med 2015;32:1036-1050
3. Nathan DM, Cleary PA, Backlund JY, Genuth SM, Lachin JM, Orchard TJ, Raskin P, Zinman B, Diabetes Control and Complications Trial / Epidemiology of Diabetes Interventions and Complications (DCCT/EDIC) Study Research Group: Intensive diabetes treatment and cardiovascular disease in patients with type 1 diabetes. N Engl J Med 2005;353:2643-2653
4. NHS Digital: National Diabetes Audit - 2015-2016: Report 1, Care Processes and Treatment Targets. http://digitalnhsuk/catalogue/PUB23241 2017;
5. Miller KM, Beck RW, Bergenstal RM, Goland RS, Haller MJ, McGill JB, Rodriguez H, Simmons JH, Hirsch IB, Network TDEC: Evidence of a Strong Association Between Frequency of Self-Monitoring of Blood Glucose and Hemoglobin A1c Levels in T1D Exchange Clinic Registry Participants. Diabetes Care 2013;36:2009-2014
6. NICE guidance NG17. Type 1 diabetes in adults: diagnosis and management https://www.nice.org.uk/guidance/ng17 lst accessed 24/2/21
7. Danne T, de Valk HW, Kracht T, Walte K, Geldmacher R, Sölter L, von dem Berge W, Welsh ZK, Bugler JR, Lange K, Kordonouri O: Reducing glycaemic variability in type 1 diabetes self-management with a continuous glucose monitoring system based on wired enzyme technology. Diabetologia 2009;52:1496-1503 *FLASH-UK Protocol Version 4.0 29th June 2020: IRAS No.: 257593 Page 110 of 111*
8. Bolinder J, Antuna R, Geelhoed-Duijvestijn P, Kroger J, Weitgasser R: Novel glucose-sensing technology and hypoglycaemia in type 1 diabetes: a multicentre, non-masked, randomised controlled trial. Lancet 2016;388:2254-2263
9. Haak T, Hanaire H, Ajjan R, Hermanns N, Riveline JP, Rayman G: Use of Flash Glucose-Sensing Technology for 12 months as a Replacement for Blood Glucose Monitoring in Insulin-treated Type 2 Diabetes. Diabetes therapy : research, treatment and education of diabetes and related disorders 2017;8:573-586
10. Dover AR, Stimson RH, Zammitt NN, Gibb FW: Flash Glucose Monitoring Improves Outcomes in a Type 1 Diabetes Clinic. J Diabetes Sci Technol 2017;11:442-443
11. McKnight JA, Gibb FW: Flash Glucose Monitoring is associated with improved glycaemic control but use is largely limited to more affluent people in a UK diabetes centre. Diabet Med 2017;34:732
12. Ish-Shalom M, Wainstein J, Raz I, Mosenzon O: Improvement in Glucose Control in Difficult-to-Control Patients With Diabetes Using a Novel Flash Glucose Monitoring Device. J Diabetes Sci Technol 2016;10:1412-1413
13. Leelarathna L, Wilmot EG. Flash forward: a review of flash glucose monitoring. Diabet Med. 2018 Apr;35(4):472-482. doi: 10.1111/dme.13584. Epub 2018 Feb 27.
14. Deshmukh H, Wilmot EG, Gregory R, Barnes D, Narendran P, Saunders S, Furlong N, Kamaruddin S, Banatwalla R, Herring R, Kilvert A, Patmore J, Walton C, Ryder REJ, Sathyapalan T. Effect of Flash Glucose Monitoring on Glycemic Control, Hypoglycemia, Diabetes-Related Distress, and Resource Utilization in the Association of British Clinical Diabetologists (ABCD) Nationwide Audit. Diabetes Care. 2020 Sep;43(9):2153-2160.
15. Rose L, Klausmann G, Seibold A. Improving HbA1c Control in Type 1 or Type 2 Diabetes Using Flash Glucose Monitoring: A Retrospective Observational Analysis in Two German Centres. Diabetes Ther. 2020 Dec 11. doi: 10.1007/s13300-020-00978-9.
16. Campbell F, Kordonouri O, Murphy N, Stewart C: FreeStyle Libre Use for Self-Management of Diabetes in Children and Adolescents. Diabetes 2017;66 Suppl. 1A:LB28-LB28
17. Boucher SE, Gray AR, Wiltshire EJ, de Bock MI, Galland BC, Tomlinson PA, Rayns JA, MacKenzie KE, Chan H, Rose S, Wheeler BJ. Effect of 6 Months of Flash Glucose Monitoring in Youth With Type 1 Diabetes and High-Risk Glycemic Control: A Randomized Controlled Trial. Diabetes Care. 2020 Oct;43(10):2388-2395. doi: 10.2337/dc20-0613.
18. Secher AL, Pedersen-Bjergaard U, Svendsen OL, Gade-Rasmussen B, Almdal TP, Dørflinger L, Vistisen D, Nørgaard K. Study protocol for optimising glycaemic control in type 1 diabetes treated with multiple daily

For peer review only - http://bmjopen.bmj.com/site/about/guidelines.xhtml

insulin injections: intermittently scanned continuous glucose monitoring, carbohydrate counting with automated bolus calculation, or both? A randomised controlled trial.
BMJ Open. 2020 Apr 27;10(4):e036474.
19. Zhou Y, Deng H, Liu H, et al. Effects of novel flash glucose monitoring system on glycaemic control in adult patients with type 1 diabetes mellitus: protocol of a multicentre randomised controlled trialBMJ Open 2020;10:e039400. doi: 10.1136/bmjopen-2020-039400
20. Alva S, Bailey T, Brazg R, Budiman ES, Castorino K, Christiansen MP, Forlenza G, Kipnes M, Liljenquist DR, Liu H. Accuracy of a 14-Day Factory-Calibrated Continuous Glucose Monitoring System With Advanced Algorithm in Pediatric and Adult Population With Diabetes. J Diabetes Sci Technol. 2020 Sep 19:1932296820958754. doi: 10.1177/1932296820958754. Online ahead of print.#
21. REPOSE Study Group: Relative effectiveness of insulin pump treatment over multiple daily injections and structured education during flexible intensive insulin treatment for type 1 diabetes: cluster randomised trial (REPOSE). BMJ. 2017 Mar 30;356:j1285. doi: 10.1136/bmj.j1285.
22. Beck RW, Riddlesworth T, Ruedy K, Ahmann A, Bergenstal R, Haller S, Kollman C, Kruger D, McGill JB, Polonsky W, Toschi E, Wolpert H, Price D, Group DS: Effect of Continuous Glucose Monitoring on Glycemic Control in Adults With Type 1 Diabetes Using Insulin Injections: The DIAMOND Randomized Clinical Trial. JAMA 2017;317:371-378
23. Lind M, Polonsky W, Hirsch IB, Heise T, Bolinder J, Dahlqvist S, Schwarz E, Olafsdottir AF, Frid A, Wedel H, Ahlen E, Nystrom T, Hellman J: Continuous Glucose Monitoring vs Conventional Therapy for Glycemic Control in Adults With Type 1 Diabetes Treated With Multiple Daily Insulin Injections: The GOLD Randomized Clinical Trial. JAMA 2017;317:379-387
24. Husereau D, Drummond M, Petrou S, Carswell C, Moher D, Greenberg D, Augustovski F, Briggs AH,Mauskopf J, Loder E: Consolidated Health Economic Evaluation Reporting Standards (CHEERS)—Explanation and Elaboration: A Report of the ISPOR Health Economic Evaluation Publication Guidelines Good Reporting. Practices Task Force. Value in Health 2013;16:231-250
25. HM Treasury. The green book: appraisal and evaluation in central government. 2003. TSO: London. 2014.
26. Palmer AJ, Roze S, Valentine WJ, Minshall ME, Foos V, Lurati FM, Lammert M, Spinas GA: Validation of theCORE Diabetes Model against epidemiological and clinical studies. Curr Med Res Opin 2004;20 Suppl 1:S27-40
27. McEwan P, Foos V, Palmer JL, Lamotte M, Lloyd A, Grant D: Validation of the IMS CORE Diabetes Model.Value in Health 17:714-724
28. Riemsma R, Corro Ramos I, Birnie R, Büyükkaramikli N, Armstrong N, Ryder S, Duffy S, Worthy G, Al M, Severens J, Kleijnen J: Integrated sensor-augmented pump therapy systems [the MiniMed® Paradigm™ Veo system and the Vibe™ and G4® PLATINUM CGM (continuous glucose monitoring) system] for managing blood glucose levels in type 1 diabetes: a systematic review and economic evaluation. Health Technology Assessment (Winchester, England) 2016;20:1-252
29. National Institute for Health and Care Excellence. Guide to the Methods of Technology Appraisal. National Institute for Clinical Excellence; 2013.
30. Bellg AJ, Borrelli B, Resnick B, Hecht J, Minicucci DS, Ory M, et al. Enhancing treatment fidelity in health behavior change studies: best practices and recommendations from the NIH Behavior Change Consortium. Health Psychol [Internet]. 2004 [cited 2012 Mar 19];23(5):443–51
31. Linnan L, Steckler A. Process evaluation for public health interventions and research: an overview. In: Linnan L, Steckler A, editors. Process evaluation for public health interventions and research. 1st ed. San Francisco: Jossey-Bass; 2002. p. 1–23.
32. Farmer T, Robinson K, Elliott SJ, Eyles J. Developing and implementing a triangulation protocol for qualitative health research. Qual Health Res. 2006;16(3):377–94.

For peer review only

For peer review only - http://bmjopen.bmj.com/site/about/guidelines.xhtml

Figure 1. Flash UK study flow chart

For peer review only

For peer review only - http://bmjopen.bmj.com/site/about/guidelines.xhtml

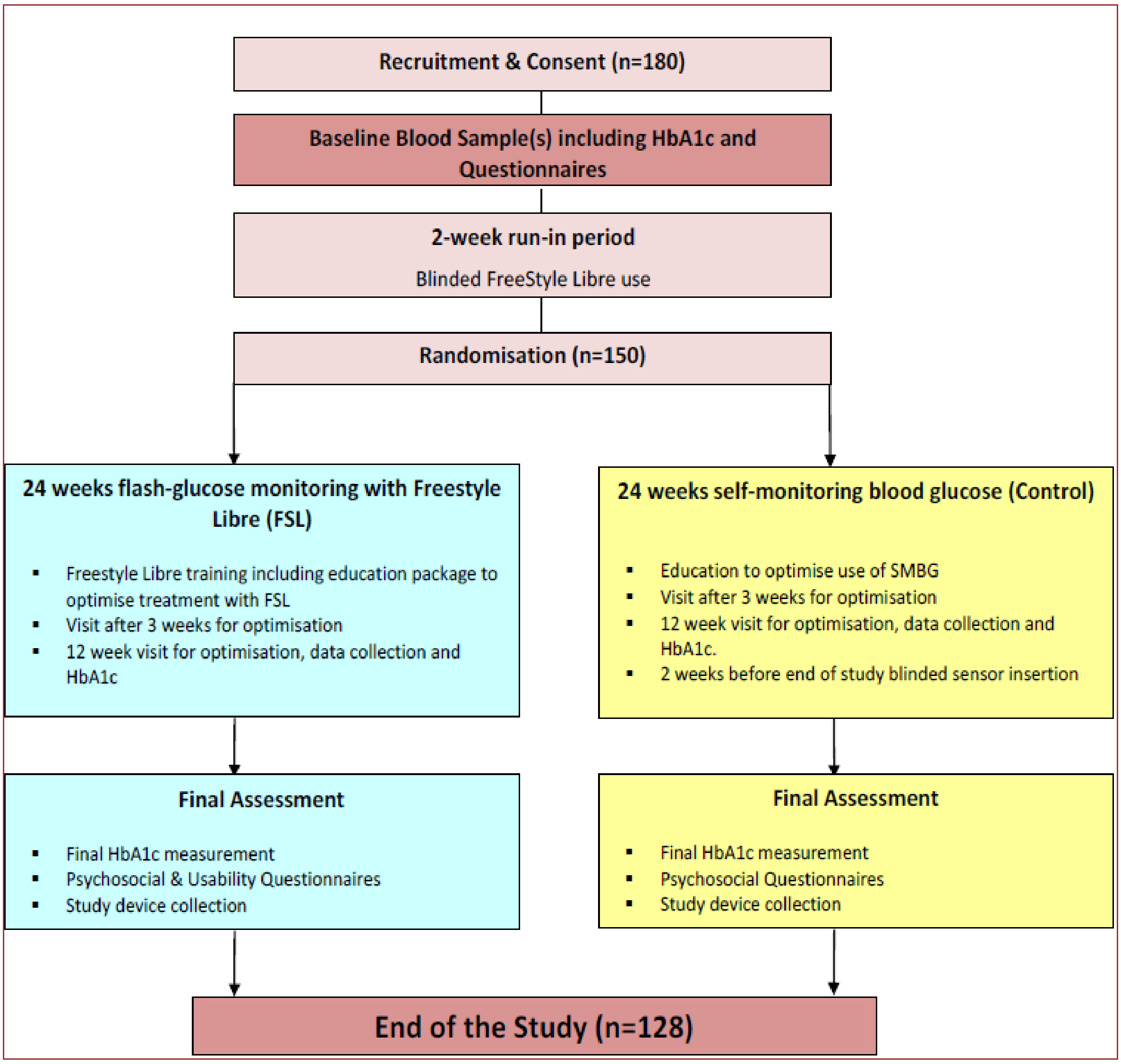
Recruitment & Consent (n=180)
Baseline Blood Sample(s) including HbA1c and Questionnaires
2-week run-in period
Blinded FreeStyle Libre use
Randomisation (n=150)
24 weeks flash-glucose monitoring with Freestyle Libre (FSL)
Freestyle Libre training including education package to optimise treatment with FSL
Visit after 3 weeks for optimisation
12 week visit for optimisation, data collection and HbA1c
24 weeks self-monitoring blood glucose (Control)
Education to optimise use of SMBG
Visit after 3 weeks for optimisation
12 week visit for optimisation, data collection and HbA1c.
2 weeks before end of study blinded sensor insertion
Final Assessment
Final HbA1c measurement
Psychosocial & Usability Questionnaires
Study device collection
Final Assessment
Final HbA1c measurement
Psychosocial Questionnaires
Study device collection
End of the Study (n=128)

Appendix 1: Data sharing plan. Clinicaltrials.gov record also updated.

Data Sharing Plan – FLASH-UK study	
Individual participant data availability (anonymised)	Yes
What data will be shared	Individual participant data that underlie the results reported in the primary study manuscript after deidentification (text, tables, figures and appendices)
What other documents will be available	The study protocol, Statistical analysis plan, informed consent form, data dictionary and statistical code.
Data availability	Beginning 6 months and ending 3 years following article publication
With whom	Researchers who provide a methodologically sound proposal not overlapping with any planned secondary publications from the research team.
For what type of analyses	To achieve aims in the approved proposal
By what mechanism will data be available	Proposals should be directed to Chief Investigator who will discuss such requested with the Trial Management Group (TMG). Lalantha.leelarathna@mft.nhs.uk.<br><br>To gain access, data requesters will need to sign a data access agreement. Data will be available for 3 years at University Of Manchester website.

For peer review only

For peer review only - http://bmjopen.bmj.com/site/about/guidelines.xhtml

Supplementary File

Flash-glucose monitoring with the FreeStyle Libre 2 compared to self-monitoring of blood glucose in sub-optimally controlled type 1 diabetes: the FLASH-UK randomised controlled trial protocol

Contents

1. Model Participant Information Sheet (Page 2)
2. Model Consent form (Page 18)
3. Data Sharing Plan (Page 20)

For peer review only

1. Model Participant Information Sheet

Flash-glucose monitoring in sub-optimally controlled Type 1 diabetes (FLASH-UK)

IRAS ID: 257593

Chief Investigator

Dr Lala Leelarathna

Lalantha.Leelarathna@mft.nhs.uk

Principal Investigators for local site

For peer review only - http://bmjopen.bmj.com/site/about/guidelines.xhtml

Flash-glucose monitoring in sub-optimally controlled Type 1 diabetes (FLASH-UK)

We would like to invite you to take part in a research study. Before you decide, it is important for you to understand why the research is being done and what it will involve. Please take time to read the following information carefully and discuss it with others if you wish.

- **Part 1 tells you the purpose of this study and what will happen to you if you take part.**
- **Part 2 gives you more detailed information about the conduct of the study.**

Ask us if there is anything that is not clear or if you would like more information. Take time to decide whether or not you wish to take part.

Part 1

Introduction

In type 1 diabetes controlling blood glucose levels is very important for good health. A number of research studies have shown that having high glucose levels in diabetes can lead to long-term problems. In order to lower the risk of these complications, maintaining glucose as close to normal range as possible is recommended. However, the achievement of near-normal glucose levels can be extremely difficult.

Currently the dose of prescribed insulin depends on the results of finger stick testing. The more frequent the testing, the easier it is to adjust the insulin dose. However many people struggle to perform frequent blood glucose tests due to pain and inconvenience.

For peer review only

The FreeStyle Libre 2 flash glucose monitor is a device which makes monitoring of glucose levels easier. It continuously monitors the glucose levels and you are able to see the level and trend by scanning the disc, worn on your arm, with a hand held device.

What is the purpose of the study?

The FreeStyle Libre device has been shown as a safe replacement for blood glucose monitoring in people with well controlled diabetes. However, there are no randomised controlled studies in adults with type 1 diabetes demonstrating improvements in HbA1c levels.

The purpose of this study is to assess the benefit of using FreeStyle Libre 2 flash glucose monitoring system, in terms of improving glucose levels as measured by HbA1c. The study will also assess the acceptability and the impact of the system on daily living with diabetes.

Why have I been invited?

You have been invited because you have Type 1 diabetes and have an HbA1c level between 58mmol/mol (7.5%) and 97mmol/mol (11%) and use either multiple daily insulin injections or use an insulin pump for controlling your blood glucose. Up to 180 participants aged 16 and above with type 1 diabetes will be involved in this project.

If you agree to take part in the study then you will be randomly allocated to either continue usual finger-stick blood glucose monitoring or Flash glucose monitoring with the FreeStyle Libre 2 device for 6 months. During this time you will either attend 5-7 research clinic visits or participate in 5-7 virtual consultations using video calls (or a combination of the two with telephone calls allowed for some visits) for data collection, device training and support to manage your diabetes. You can decide whether to attend clinic visits or virtual assessments but this will also depend on whether your hospital is operating normal clinics because of the COVID-19 pandemic.

Do I have to take part in this research?

No. It is up to you to decide whether or not to take part. If you do, you will be given more detailed information and will be asked to sign a consent form. You are still free to withdraw at any time and

For peer review only - http://bmjopen.bmj.com/site/about/guidelines.xhtml

without giving any reason. A decision to withdraw at any time, or a decision not to take part, will not affect the standard of care you receive.

What will happen to me if I take part?

The study consists of 5-7 visits with the diabetes research team (either in person or by virtual consultation) over a 6 month period. During these visits you will be supported to improve your diabetes control. Blood tests are done three times during this 6 month period. If you attend virtual consultations the information sheet / consent form (for visit 1), necessary questionnaires or blood collection kit will be provided by post before these take place and they can be returned by pre-paid postal delivery.

The following sections describe the study visits in detail.

Visit 1 - Recruitment visit (up to 2 hours)

Once you have had sufficient time to read this information sheet, if you would like to hear more about the study or would like to participate, you will be invited to attend the recruitment visit (either in person or by virtual consultation). You will be provided with detailed information about the study and any questions you have will be answered. If you decide to participate, you will be asked to sign a consent form. By signing the form you are telling us that you understand what you have read and discussed, consent to take part in the research project and consent to the use of your information as described.

If would like to consider joining the trial by virtual consultation, you will receive the consent form along with this information sheet by post before the recruitment visit is scheduled. Any questions you have can be answered by email, telephone or video consultation. If you decide to participate, you will need to provide initials to each declaration box on the consent form, sign it and return it to the research team in a pre-paid envelope. Once the research team receive your fully completed consent form, they will schedule your virtual recruitment consultation. At the start of the virtual recruitment consultation, the investigator will ask if you are happy to proceed and they will sign / date the consent form. A copy of the fully completed consent form will be returned to you. Once you receive this, you can let the research team know before or at your next visit.

For peer review only - http://bmjopen.bmj.com/site/about/guidelines.xhtml

At recruitment visit, if you consent to take part the following data will be captured:

History: medical and diabetes history, current medications and insulin therapy recorded.

Measurements: Height, weight (if possible).

Blood test : HbA1c, (less than 10 mls). If conducted as a virtual visit, we will send you a pre-paid pack to collect blood into a small tube from a finger-stick. This can be returned by post in the pre-paid envelop provided.

Questionnaires: to assess a range of measures which include diabetes treatment satisfaction, diabetes distress, quality of life, fear of injections/testing, eating behaviours, hypoglycaemia. If conducted as a virtual consultation, we will send you a pre-paid envelope to return any questionnaires that cannot be completed by video call.

Personal Data: Initials, Date of Birth, Gender & Full Postcode.

If you intend to become pregnant within the next six months then you should notify the research team. If you are pregnant at the recruitment visit, or should you become pregnant during the study, you will not be able to take any further part.

As with many research studies, in order to proceed with the study certain recruitment criteria need to be met. In the event you don't meet the recruitment criteria your participation in the study will end after visit 1.

Visit 2 (Or combined with visit 1) – Blinded flash glucose monitor insertion (30 minutes)

Within 1 to 2 weeks of visit 1 (or at the same time as visit 1 if easier and recruitment criteria are met), a glucose sensor will be inserted to collect information about glucose data. The sensor looks the same as the Freestyle Libre sensor and is placed in the same area. The purpose of this is to compare results at the end with the results at the beginning. In order to avoid any change in behaviour or insulin doses, glucose sensor data will not be visible to you (Blinded sensor) during these first 2 weeks. It is important that you do not try to obtain the blinded glucose data as this will affect the comparison of before and after data. You are asked to wear the sensor for 14 days. If this visit is conducted as a virtual consultation we will send you the sensor and reader for self-insertion and provide guidance on how to apply the sensor. After 14 days you will need to post the sensor and reader back to us in a pre-paid envelope provided.

For peer review only - http://bmjopen.bmj.com/site/about/guidelines.xhtml

Visit 3 – Randomisation, start of study treatment & education (up to 2 hours)

During Visit 3, we will check that you were comfortable wearing the flash glucose monitor for the 14 days. (To proceed with the study the blinded glucose sensor should have been worn for a minimum of 10 days'). If you are happy to proceed you will be randomly assigned to either flash glucose monitoring with the FreeStyle Libre 2 device for 6 months or continuing with finger-stick glucose tests for 6 months.

Visit 3:

Randomisation: to either flash glucose monitoring or finger-stick glucose tests for the next 6 months.

Education and support: If you are allocated to flash glucose monitoring, the research team will spend some time demonstrating how to insert the device, what the data means and how you safely use the device. This will include a discussion of how to use the device to review data and make adjustments to your insulin therapy. You will be shown how to download and review data at home. If you are continuing with finger-stick blood tests the study team will review your diabetes therapy and support you to make improvements. For those attending virtual consultations and who are assigned to the flash glucose monitoring arm, the research team will send you all the devices you need and easy to follow instructions and links to self-help videos. The Research team will conduct additional training through video consultation as required.

Given participant diary: to record information about insulin doses and carbohydrate intake in last 5 days before study visits 4, 5 and 7. If you are attending virtual consultations, the research team will send diaries for you to complete and these can be returned by pre-paid postal delivery or scanned and returned by email.

Downloading of devices: data from your glucose meter (and insulin pump if you are on one) will be downloaded where possible.

Visit 4 – Data review and education support (up to 1 hour)

For peer review only - http://bmjopen.bmj.com/site/about/guidelines.xhtml

Four weeks after visit 3 you will be asked to come to the clinic or attend a virtual clinic consultation, with your glucose meter (flash glucose reader or blood glucose meter) and the completed diary which captures your insulin doses and carbohydrate intake. The research team will work with you to review your insulin therapy, supporting you to adjust your insulin to improve your diabetes control. Data from your glucose meters (and insulin pump if you are on one) will be downloaded to secure web-based programmes similar to what is used in clinical practice.

Visit 5 - Data review and education support (up to 2 hours)

Eight weeks after visit 4 you will be seen in clinic or by virtual clinic consultation. The purpose of this visit is to provide ongoing support and training to help you improve your diabetes control. You are required to bring your glucose meters and diary with insulin doses. A HbA1c blood test will also be performed at this visit (blood sample collected at home and returned by pre-paid envelope for those attending virtual consultations). Data from your glucose meters (and insulin pump if you are on one) will be downloaded to secure web-based programmes similar to what is used in clinical practice.

Visit 6 – Finger-stick glucose testing group only: blinded sensor (<30 minutes)

If you are allocated to the finger-stick glucose testing group you will be asked to return to clinic or attend a virtual consultation 10 weeks after visit 5 to have a blinded sensor attached. If conducted as a virtual consultation, we will send you the devices you need and easy to follow instructions and self-help videos (like visit 1). The blinded FreeStyle Libre sensor will be worn for 2 weeks. This will allow the collection of glucose data to see how your diabetes control has changed compared to your glucose control when you entered the study. After 14 days you will need to post the sensor and reader back to us by pre-paid envelope if you attended a virtual consultation.

Visit 7 - End of study visit (upto 2 hours)

Approximately 12 weeks after Visit 5 (or 2 weeks after visit 6 for the finger-stick glucose testing group), you will be asked to attend the clinic or virtual consultation. This will be the final visit. Data from your glucose meters (and insulin pump if you are on one) will be downloaded to secure web-based programmes similar to what is used in clinical practice where possible. We will also collect paper diaries.

For peer review only - http://bmjopen.bmj.com/site/about/guidelines.xhtml

During visit 7 the following occurs:

Blood test: HbA1c (blood sample collected at home for virtual consultations).

Questionnaires: same as those completed at the start of the study. In addition if you were randomised to Flash glucose monitoring arm, you will be invited to complete two additional questionnaires to capture your experience and expectations during the study (If you attend a virtual consultation any questionnaires that cannot be completed will be provided for return in a pre-paid envelope).

Data download and review: to support you to improve glucose control.

Weight measured: where possible.

Communication with research team for advice and support by e-mail

If you choose to communicate with us by e-mail, we will use a dedicated e-mail account as a way to communicate with you. We will use reasonable means to protect the security and confidentiality of e-mail information sent and received. The research team will take every care to remove any identifying material from the responses you provide as early as possible. Likewise, individuals' responses will be kept confidential by the researcher and participants will not be identified in the reporting of the research. However, the researcher cannot guarantee the confidentiality or anonymity of material transferred by email or the internet. There are known and unknown risks that may affect privacy when using e-mail to communicate. These risks include, but are not limited, to:

- E-mail can be forwarded, printed, and stored in numerous paper and electronic forms and be received by many intended and unintended recipients without my knowledge or agreement.
- E-mail may be sent to the wrong address by any sender or receiver.
- E-mail is easier to forge than handwritten or signed papers.
- Copies of e-mail may exist even after the sender or the receiver has deleted his or her copy.
- E-mail service providers have a right to keep and inspect emails sent through their systems.
- E-mail may be intercepted and read during transmission without detection or authorisation.
- E-mail is not a secure way of corresponding and it is advisable not to transfer any sensitive information in this format.
- E-mail can spread computer viruses.

Choosing to participate in the trial by virtual consultation

For peer review only - http://bmjopen.bmj.com/site/about/guidelines.xhtml

If you choose to participate in the trial by use of audio / video communication, we will use a dedicated videoconferencing account as a way to communicate with you. We will use reasonable means to protect the security of the connection and virtual consultations will not be recorded. However, the researcher cannot guarantee the confidentiality or anonymity of material transferred over the internet and stored within devices. There is an increased security risk that your health information may be intercepted or disclosed to third parties when using video or audio communications tools. To help us keep your information safe and secure, you can:

- Understand that this method of communication is not secure in the same way as a private appointment in an exam room.
- Use a private computer / device (i.e., not an employer's or third party's computer / device) and a secure internet connection. For example, using a personal computer or tablet is more secure than at a library, and your access to the Internet on your home network will generally be more secure than an open guest Wi-Fi connections.

By providing your information, you agree to let us collect, use, or disclose your personal health information through video or audio communications (while following applicable privacy laws) in order to provide you with care. In particular, the following means of electronic communication may be used for videoconferencing, including Skype, Microsoft Teams and Zoom.

Will I be reimbursed?

There is no payment for taking part in the study. If you are randomised to the flash glucose monitoring arm then the sensors will be provided to you for 6 months, free of charge. The blinded sensors that will be provided at Visit 1 or 2 and at visit 6 (those assigned to finger-stick glucose testing) will also be provided free of charge. Reasonable travelling expenses will be reimbursed if you attend any visits in person at the study clinics.

What are the possible risks of taking part?

During this study, we will support you to improve your glucose control. Sometimes when improving glucose control you may experience a 'hypo', similar to the ones that happen normally in everyday life. We will ask you to treat it in the same way you normally treat a hypo. There is a risk of possible mild to moderate hyperglycaemia (high glucose) which is similar to the risk that a person with type 1 diabetes experiences on a daily basis. There is also a risk of hyperglycaemia leading to diabetic ketoacidosis (for example if the pump is blocked for a significant time or if you miss insulin doses or

For peer review only - http://bmjopen.bmj.com/site/about/guidelines.xhtml

become unwell); however this risk is low and similar to the risk that a person with type 1 diabetes experiences.

You will have to attach a small glucose sensor on the upper arm. The sensor needs to be changed every 14 days. Inserting the sensor has a low risk of developing a local skin reaction or infection. Some bruising, itchiness, redness and bleeding at the site of sensor insertion may occur.

In addition, when the blood samples are taken for the study, some bruising or minor discomfort may also occur at the site where the blood was taken.

What are the possible benefits of taking part?

You will have regular contact with the study team who will support you to improve your glucose control and this may have benefits beyond the study period. Results of the study may pave the way for better access for flash glucose monitoring for people living with type 1 diabetes.

Who should I talk to if I have any questions or concerns?

If you have any questions regarding this study, please contact one of the following people:

Contact details of the local clinical study team

On behalf of the Study Team, we would like to thank you for taking the time to consider participating in this important research programme.

This completes part 1 of the Information Sheet.

For peer review only - http://bmjopen.bmj.com/site/about/guidelines.xhtml

If the information in Part 1 has interested you and you are considering participation, please continue to read the additional information in Part 2 before making any decision.

For peer review only

For peer review only - http://bmjopen.bmj.com/site/about/guidelines.xhtml

Part 2

What if relevant new information becomes available?

You will be informed as soon as possible if any new information becomes available during the course of the study that may affect your willingness to participate in the study. If you decide not to continue, the study doctor will arrange for your care to continue.

What will happen if I don't want to carry on with the study?

If you decide not to participate in this study, it will not affect your future management in any way. If you agree to take part, please remember that you are free to withdraw at any time without explanation. All you need to do is tell us. If you withdraw from the study, we will destroy all your identifiable samples, but we will need to use the data collected up to your withdrawal.

What if there is a problem?

If you have a concern about any aspect of this study, please ask to speak to the researchers who will do their best to answer your questions (see contact details above). If you are unhappy about the conduct of the study and wish to complain, you can do this through (name and contact details of the local patient advice and liaison service – institution specific).

In the unlikely event that something does go wrong and you are harmed during the research study, appropriate healthcare arrangements will be made. Healthcare arrangements may include advice from clinical members of the study team or your local diabetes clinic team, or use of emergency health services. There are no special compensation arrangements unless this was due to the negligence of one of the doctors or nurses. In this case you may have grounds for legal action for compensation but you may have to pay your legal costs. The normal hospital complaints mechanism will still be available to you.

Under what circumstances might the study be stopped?

The study may be stopped, or you will be asked to withdraw from the study, under the following circumstances:

For peer review only - http://bmjopen.bmj.com/site/about/guidelines.xhtml

- Serious harm to any participant in the study
- You have significant difficulties using the study devices during the training period(s)
- You develop significant allergy to the plaster holding the sensor
- Failure to follow the instructions of the study team and misuse of the study devices
- Decision by the study team, or the sponsor, that stopping the study is in your best medical interest
- Pregnancy, planned pregnancy, or breast feeding
- Technical reasons (e.g. moving home outside the region)

Should the study be stopped for any reason, then you will be informed as soon as possible and your doctor will arrange for your care to continue.

Will my GP be informed?

We will inform your GP of your participation in this study after having gained your consent for this.

Will my taking part in the study be kept confidential?

Strict confidentiality will be maintained at all times. All collected written data will be kept in a locked cupboard, accessible only by the research team.

Only the researchers who are directly involved in the study, the study sponsor (Manchester University NHS Foundation Trust), the study monitor and regulatory authority who will be involved in monitoring and auditing of the project will have access to the identifiable data, as permitted by applicable laws and regulations. By signing the written informed consent form, you are authorising such access. Anonymised data collected during the study may also be sent to associated researchers in EU and USA where the laws do not protect your privacy to the same extent as the law in the UK. We will however take all reasonable steps to protect your privacy. Data will be stored for 15 years and will be disposed of securely thereafter.

For peer review only - http://bmjopen.bmj.com/site/about/guidelines.xhtml

Compliance with General Data Protection (GDPR) Rules

Manchester University NHS Foundation Trust is the sponsor for this study based in the United Kingdom. We will be using information from you and/or your medical records in order to undertake this study and will act as the data controller for this study. This means that we are responsible for looking after your information and using it properly. Manchester University NHS Foundation Trust will keep identifiable information about you [for three years after the study has finished].

Your rights to access, change or move your information are limited, as we need to manage your information in specific ways in order for the research to be reliable and accurate. If you withdraw from the study, we will keep the information about you that we have already obtained. To safeguard your rights, we will use the minimum personally-identifiable information possible.

You can find out more about how we use your information at https://research.cmft.nhs.uk/getting-involved/gdpr-and-research and by contacting our Data Protection Officer at dpo@mft.nhs.uk.

[Name of NHS hospital] will collect information from you and/or your medical records for this research study in accordance with our instructions.

[Name of NHS hospital] will keep your name, [NHS number] and contact details [add other identifiers] confidential and will not pass this information to Manchester University NHS Foundation Trust. [Name of NHS hospital] will use this information as needed, to contact you about the research study, and make sure that relevant information about the study is recorded for your care, and to oversee the quality of the study. Certain individuals from Manchester University NHS Foundation trust, Manchester Clinical Trials Unit and regulatory organisations may look at your medical and research records to check the accuracy of the research study. Manchester University NHS Foundation Trust will only receive information without any identifying information. The people who analyse the information

For peer review only - http://bmjopen.bmj.com/site/about/guidelines.xhtml

will not be able to identify you and will not be able to find out your name, [NHS number] or contact details.

[Name of NHS hospital] will keep identifiable information about you from this study for three years after the study has finished.

What will happen to any samples I give?

As above, blood samples collected during this study will be labelled and sent to the lab for processing, in line with standard accredited practice. Once the measurements have been completed the samples will be disposed of securely.

What will happen to the data collected?

After each visit fully anonymised data will be entered onto a computer and will be used to analyse the results once the study is finished. These study results may be presented at scientific meetings or published in a scientific journal. In addition, fully anonymised data may be shared with researchers and collaborating partners.

Your personal details will be kept for up to 3 years after the end of the study so that we can inform you the results of the study.

Who is organising and funding the research?

The study will be coordinated from the Manchester Clinical Trials Unit, University of Manchester . The research study has been funded by Diabetes UK charity. The sponsor of the study is the Manchester University NHS Foundation Trust.

Who has reviewed the study?

Before any research goes ahead it has to be checked by an Ethics Committee. This project has been reviewed by the Greater Manchester West Research Ethics Committee.

For peer review only

If I agree to join the study

The study will be explained to you in more detail during the recruitment visit. During this visit you will be able to ask questions and voice any queries. Once you have agreed to take part we will ask you to sign a consent form. The study team will then arrange the dates for your training and hospital visits.

What will happen at the end of the study?

At the end of this study you will go back to using your usual glucose monitoring device. Your diabetes care will then be looked after by your own diabetes team.

For peer review only

This completes part 2 of the Information Sheet. Thank you for your time and interest in our research.

(site/institution address)

For peer review only - http://bmjopen.bmj.com/site/about/guidelines.xhtml

2. Consent form

PARTICIPANT CONSENT FORM

Title of Project: Flash-glucose monitoring in sub-optimally controlled type 1 diabetes (FLASH-UK), IRAS ID 257593

	Please Provide Initials in Each Box
Name of Principal Investigator: Dr	
I confirm that I have read and understood the information sheet version .... dated .... for the above study and have had the opportunity to consider the information and I am satisfied with the answers I have been given.	☐
I understand that my participation is voluntary and that I am free to withdraw at any time, without giving any reason, without my medical care or legal rights being affected in any way.	☐
I give consent for the taking of blood samples.	☐
If I want to change my mind in the future and withdraw my consent for these studies, then I understand that, if I request it, the identifiable data collected will be destroyed. Unidentifiable data would be retained.	☐
I give consent to my GP being informed of my participation in this study.	☐
I give consent to the sharing of anonymised research data arising from this study with other study partners in Europe and USA.	☐
If I choose to communicate with the research team via email / virtual consultation, I acknowledge the associated risks as outlined in the information sheet.	☐

For peer review only - http://bmjopen.bmj.com/site/about/guidelines.xhtml

I understand that relevant sections of my medical notes and data collected during the study may be looked at by individuals from the NHS Trust, the sponsor (or their delegate) and regulatory bodies for audit or monitoring purposes. ☐

I agree to return the study devices at the end of the study or earlier if consent is withdrawn. ☐

I agree to follow instructions from the study team and take part in the study. ☐

___________________________	__________________	______________________
Name of patient	Date	Signature
___________________________	__________________	______________________
Name of Researcher taking consent	Date	Signature

Copies: *1 for patient; 1 for researcher; 1 to be kept with hospital notes*

For peer review only

For peer review only - http://bmjopen.bmj.com/site/about/guidelines.xhtml

3. Data Sharing plan

Data Sharing Plan – FLASH-UK study	
Individual participant data availability (anonymised)	Yes
What data will be shared	Individual participant data that underlie the results reported in the primary study manuscript after deidentification (text, tables, figures and appendices)
What other documents will be available	The study protocol, Statistical analysis plan, Informed consent form, data dictionary and statistical code.
Data availability	Beginning 6 months and ending 3 years following article publication
With whom	Researchers who provide a methodologically sound proposal not overlapping with any planned secondary publications from the research team.
For what type of analyses	To achieve aims in the approved proposal
By what mechanism will data be available	Proposals should be directed to Chief Investigator who will discuss such requested with the Trial Management Group (TMG). Lalantha.leelarathna@mft.nhs.uk.<br><br>To gain access, data requesters will need to sign a data access agreement. Data will be available for 3 years.

For peer review only - http://bmjopen.bmj.com/site/about/guidelines.xhtml

STANDARD PROTOCOL ITEMS: RECOMMENDATIONS FOR INTERVENTIONAL TRIALS

SPIRIT 2013 Checklist: Recommended items to address in a clinical trial protocol and related documents*

Section/item	Item No	Description	Page number
**Administrative information**			
Title	1	Descriptive title identifying the study design, population, interventions, and, if applicable, trial acronym	1
Trial registration	2a	Trial identifier and registry name. If not yet registered, name of intended registry	2
	2b	All items from the World Health Organization Trial Registration Data Set	
Protocol version	3	Date and version identifier	3
Funding	4	Sources and types of financial, material, and other support	19
Roles and responsibilities	5a	Names, affiliations, and roles of protocol contributors	3, 19
	5b	Name and contact information for the trial sponsor	3
	5c	Role of study sponsor and funders, if any, in study design; collection, management, analysis, and interpretation of data; writing of the report; and the decision to submit the report for publication, including whether they will have ultimate authority over any of these activities	19
	5d	Composition, roles, and responsibilities of the coordinating centre, steering committee, endpoint adjudication committee, data management team, and other individuals or groups overseeing the trial, if applicable (see Item 21a for data monitoring committee)	14
**Introduction**			
Background and rationale	6a	Description of research question and justification for undertaking the trial, including summary of relevant studies (published and unpublished) examining benefits and harms for each intervention	5-6
	6b	Explanation for choice of comparators	5

For peer review only

Objectives	7	Specific objectives or hypotheses	6
Trial design	8	Description of trial design including type of trial (eg, parallel group, crossover, factorial, single group), allocation ratio, and framework (eg, superiority, equivalence, noninferiority, exploratory)	6
**Methods: Participants, interventions, and outcomes**			
Study setting	9	Description of study settings (eg, community clinic, academic hospital) and list of countries where data will be collected. Reference to where list of study sites can be obtained	6-7
Eligibility criteria	10	Inclusion and exclusion criteria for participants. If applicable, eligibility criteria for study centres and individuals who will perform the interventions (eg, surgeons, psychotherapists)	16
Interventions	11a	Interventions for each group with sufficient detail to allow replication, including how and when they will be administered	7
	11b	Criteria for discontinuing or modifying allocated interventions for a given trial participant (eg, drug dose change in response to harms, participant request, or improving/worsening disease)	9
	11c	Strategies to improve adherence to intervention protocols, and any procedures for monitoring adherence (eg, drug tablet return, laboratory tests)	8,18, table 2
	11d	Relevant concomitant care and interventions that are permitted or prohibited during the trial	16
Outcomes	12	Primary, secondary, and other outcomes, including the specific measurement variable (eg, systolic blood pressure), analysis metric (eg, change from baseline, final value, time to event), method of aggregation (eg, median, proportion), and time point for each outcome. Explanation of the clinical relevance of chosen efficacy and harm outcomes is strongly recommended	8,17, table 1
Participant timeline	13	Time schedule of enrolment, interventions (including any run-ins and washouts), assessments, and visits for participants. A schematic diagram is highly recommended (see Figure)	18, table 2

For peer review only - http://bmjopen.bmj.com/site/about/guidelines.xhtml

Sample size	14	Estimated number of participants needed to achieve study objectives and how it was determined, including clinical and statistical assumptions supporting any sample size calculations	9/10
Recruitment	15	Strategies for achieving adequate participant enrolment to reach target sample size	10
**Methods: Assignment of interventions (for controlled trials)**			
Allocation:			
Sequence generation	16a	Method of generating the allocation sequence (eg, computer-generated random numbers), and list of any factors for stratification. To reduce predictability of a random sequence, details of any planned restriction (eg, blocking) should be provided in a separate document that is unavailable to those who enrol participants or assign interventions	10
Allocation concealment mechanism	16b	Mechanism of implementing the allocation sequence (eg, central telephone; sequentially numbered, opaque, sealed envelopes), describing any steps to conceal the sequence until interventions are assigned	10
Implementation	16c	Who will generate the allocation sequence, who will enrol participants, and who will assign participants to interventions	10
Blinding (masking)	17a	Who will be blinded after assignment to interventions (eg, trial participants, care providers, outcome assessors, data analysts), and how	10
	17b	If blinded, circumstances under which unblinding is permissible, and procedure for revealing a participant's allocated intervention during the trial	na
**Methods: Data collection, management, and analysis**			
Data collection methods	18a	Plans for assessment and collection of outcome, baseline, and other trial data, including any related processes to promote data quality (eg, duplicate measurements, training of assessors) and a description of study instruments (eg, questionnaires, laboratory tests) along with their reliability and validity, if known. Reference to where data collection forms can be found, if not in the protocol	protocol
	18b	Plans to promote participant retention and complete follow-up, including list of any outcome data to be collected for participants who discontinue or deviate from intervention protocols	10

For peer review only

Data management	19	Plans for data entry, coding, security, and storage, including any related processes to promote data quality (eg, double data entry; range checks for data values). Reference to where details of data management procedures can be found, if not in the protocol	11
Statistical methods	20a	Statistical methods for analysing primary and secondary outcomes. Reference to where other details of the statistical analysis plan can be found, if not in the protocol	11/12
	20b	Methods for any additional analyses (eg, subgroup and adjusted analyses)	11/12/13
	20c	Definition of analysis population relating to protocol non-adherence (eg, as randomised analysis), and any statistical methods to handle missing data (eg, multiple imputation)	11/12
**Methods: Monitoring**			
Data monitoring	21a	Composition of data monitoring committee (DMC); summary of its role and reporting structure; statement of whether it is independent from the sponsor and competing interests; and reference to where further details about its charter can be found, if not in the protocol. Alternatively, an explanation of why a DMC is not needed	14
	21b	Description of any interim analyses and stopping guidelines, including who will have access to these interim results and make the final decision to terminate the trial	NA
Harms	22	Plans for collecting, assessing, reporting, and managing solicited and spontaneously reported adverse events and other unintended effects of trial interventions or trial conduct	9,14
Auditing	23	Frequency and procedures for auditing trial conduct, if any, and whether the process will be independent from investigators and the sponsor	14
**Ethics and dissemination**			
Research ethics approval	24	Plans for seeking research ethics committee/institutional review board (REC/IRB) approval	15
Protocol amendments	25	Plans for communicating important protocol modifications (eg, changes to eligibility criteria, outcomes, analyses) to relevant parties (eg, investigators, REC/IRBs, trial participants, trial registries, journals, regulators)	15
Consent or assent	26a	Who will obtain informed consent or assent from potential trial participants or authorised surrogates, and how (see Item 32)	8
	26b	Additional consent provisions for collection and use of participant data and biological specimens in ancillary studies, if applicable	NA

For peer review only

For peer review only - http://bmjopen.bmj.com/site/about/guidelines.xhtml

Confidentiality	27	How personal information about potential and enrolled participants will be collected, shared, and maintained in order to protect confidentiality before, during, and after the trial	11
Declaration of interests	28	Financial and other competing interests for principal investigators for the overall trial and each study site	19
Access to data	29	Statement of who will have access to the final trial dataset, and disclosure of contractual agreements that limit such access for investigators	11
Ancillary and post-trial care	30	Provisions, if any, for ancillary and post-trial care, and for compensation to those who suffer harm from trial participation	Main protocol
Dissemination policy	31a	Plans for investigators and sponsor to communicate trial results to participants, healthcare professionals, the public, and other relevant groups (eg, via publication, reporting in results databases, or other data sharing arrangements), including any publication restrictions	15
	31b	Authorship eligibility guidelines and any intended use of professional writers	19
	31c	Plans, if any, for granting public access to the full protocol, participant-level dataset, and statistical code	22
**Appendices**			
Informed consent materials	32	Model consent form and other related documentation given to participants and authorised surrogates	Submitted
Biological specimens	33	Plans for collection, laboratory evaluation, and storage of biological specimens for genetic or molecular analysis in the current trial and for future use in ancillary studies, if applicable	NA

*It is strongly recommended that this checklist be read in conjunction with the SPIRIT 2013 Explanation & Elaboration for important clarification on the items. Amendments to the protocol should be tracked and dated. The SPIRIT checklist is copyrighted by the SPIRIT Group under the Creative Commons "Attribution-NonCommercial-NoDerivs 3.0 Unported" license.
